# Scaling relations for auxin waves

**DOI:** 10.1007/s00285-022-01793-5

**Published:** 2022-09-26

**Authors:** Bente Hilde Bakker, Timothy E. Faver, Hermen Jan Hupkes, Roeland M. H. Merks, Jelle van der Voort

**Affiliations:** 1 Mathematical Institute, Universiteit Leiden, P.O. Box 9512, 2300 RA Leiden, The Netherlands; 2 Department of Mathematics, Kennesaw State University, 850 Polytechnic Lane, MD #9085, Marietta, GA 30060 USA; 3 Mathematical Institute and Institute of Biology Leiden, Universiteit Leiden, P.O. Box 9512, 2300 RA Leiden, The Netherlands

**Keywords:** Travelling waves, Polar auxin transport, Up-the-gradient models, Scaling limits, Cross-diffusion, Lattice differential equations, Primary 34A33, 92C37, Secondary 34K26

## Abstract

We analyze an ‘up-the-gradient’ model for the formation of transport channels of the phytohormone auxin, through auxin-mediated polarization of the PIN1 auxin transporter. We show that this model admits a family of travelling wave solutions that is parameterized by the height of the auxin-pulse. We uncover scaling relations for the speed and width of these waves and verify these rigorous results with numerical computations. In addition, we provide explicit expressions for the leading-order wave profiles, which allows the influence of the biological parameters in the problem to be readily identified. Our proofs are based on a generalization of the scaling principle developed by Friesecke and Pego to construct pulse solutions to the classic Fermi–Pasta–Ulam–Tsingou model, which describes a one-dimensional chain of coupled nonlinear springs.

**Supplementary Information:**

The online version contains supplementary material available at 10.1007/s00285-022-01793-5.

## Introduction

### Polar auxin transport

The phytohormone auxin is a central player in practically all aspects of the development and growth of plants, for example in phyllotaxis, root development and the initiation of lateral roots, the formation of vascular tissues in stems, the patterning of leaf veins, and flower development (Paque and Weijers 2016). The pattern formation principles underlying these developmental mechanisms have been uncovered to a large part through an intensive cross-talk between experimental approaches and mathematical modeling (Shi and Vernoux 2018; Autran et al. 2021; Cieslak et al. 2021). Auxin is transported between cells and between cells and the cell walls both through diffusion and through transport proteins that are localized at the plasma membrane (PM). Some of these transport proteins, mostly notably several members of the PIN-FORMED family including PIN1 (Adamowski and Friml 2015) are distributed in a polarised manner inside the cells. Such polarised localisation of PINs is coordinated in plant tissue, leading to a directed transport of auxin through plant tissues in a mechanism called polar auxin transport (PAT) (Adamowski and Friml 2015). For example, in fully developed seed plants, auxin is synthesized in leaves, then is transported through the central tissues of the stem and the root towards the root tips, where it redirected along the superficial tissues of the root back to towards the stem and recycled towards the internal tissues of the root (Adamowski and Friml 2015).

Despite new details being uncovered incessantly (see e.g. Verna et al. 2019; Hajný et al. 2020), it is still incompletely understood what mechanisms drive the polarization of PINs inside cells and the coordinated polarization among adjacent cells. In a series of classical experiments, Sachs applied artificial auxin to bean plants, and observed that these become the source of new vascular tissue that then joins the existing vasculature; see e.g. Sachs (1975) and the review (Hajný et al. 2022). These initial observations, together with the discovery of PIN1 and subsequently discovered members of the PIN-FORMED protein family suggested that auxin drives the polarization of its own transporters, and hence the direction of its own transport (reviewed in Merks et al. 2007; Hajný et al. 2022). Initial models aimed to explain the formation of transport channels as observed in Sachs’ experiments. These models therefore assumed that the rate of auxin flux from cell to cell further polarised auxin transport. This positive feedback led to the self-organised formation of auxin transport channels in a process called auxin canalisation. When it was realised that auxin accumulations mark the formation of new leaves at the shoot apex, an alternative model was proposed, in which cells polarised towards the locally increased concentrations of auxin, thus forming self-organised accumulation of auxin (Reinhardt et al. 2003). Mathematical models of the self-organisation of polar auxin transport therefore follow these two broad categories. ‘With-the-gradient’ models formalise the canalisation hypothesis and assume that the rate of cell polarisation depends on the auxin *flux* towards the relevant neighbour (Mitchison 1980, 1981; Rolland-Lagan and Prusinkiewicz 2005; Rolland-Lagan 2008). ‘Up-the-gradient’ models assume that PIN polarizes in the direction of neighbouring cells at a rate that positively depends on the auxin *concentration* in that neighbour (Jönsson et al. 2006; Smith et al. 2006). Attempts to reconcile these two seemingly contradicting ideas have followed two broad approaches. The first approach proposed that with-the-gradient and up-the-gradient models act at different positions of the plant or at different stages during development. For example Bayer et al. (2009) proposed that the up-the-gradient model act at superficial tissue layers of the shoot apical meristem where it forms auxin accumulation points leading to the initial of new leaves. The deeper tissue layers could follow the with-the-gradient model channeling auxin away from the auxin accumulation point towards the vascular tissues (Bayer et al. 2009). A similar approach was recently taken to explain the leaf venation patterning in combination with auxin convergence at the edge of the leaf primordium (Holloway and Wenzel 2021). The second approach looked for variants of the with-the-gradient or up-the-gradient models that could explain both auxin canalisation and auxin accumulation depending on the parameter settings. In this line of reasoning Walker et al. have proposed a with-the-gradient hypothesis for phyllotaxis (Walke et al. 2013), whereas one of us has proposed an up-the-gradient hypothesis for canalisation (Merks et al. 2007).

More recent analyses of the role of auxin and PINs in the formation of leaf veins (Verna et al. 2019) put the key role of a feedback between auxin signaling and the polar localisation of PINs into question, and therefore the validity of canalisation hypothesis or its alternatives including the traveling-wave hypothesis (Merks et al. 2007) for formation of vascular tissues. In particular, quadruple mutants strongly reducing functionality of all plasma-membrane-localised PINs, i.e., of all PINs that are responsible for PAT in the leaf veins, show relatively mild venation pattern phenotypes. Further knock-out of PIN6 and PIN8, expressed in the leaf veins but not localised in the PM, thus excluding a role of these PINs in PAT, led to further defects in leaf venation patterning (Verna et al. 2019) identical to those due to a chemical block of auxin transport. Nevertheless, in these mutants the polar ordening of the cells in the vasculature stays intact and supernumerary veins are induced by exogenous application of auxin, showing that auxin can induce veins in absence of polar transport. Using further mutations of auxin sensing proteins, it was found that this PAT-independent vein formation requires auxin sensing and the activity of GNOM, a protein regulating the constitutive recycling of PM-localised proteins, including PINs. How the available mathematical models of auxin-regulated patterning in plants will need to be updated or rejected is a topic of ongoing investigation, but what seems clear at this moment is that such models must involve auxin sensing and coordination of cell polarisation possibly through polar transport of other small chemicals besides auxine [e.g., acidification of the cell walls (Fendrych et al. 2016)], facilitated diffusion (Mitchison 1980) or coordination of polarity through other means such as mechanical signaling as studied in mathematical models of phyllotaxis (Julien et al. 2019) and leaf venation patterning (Kneuper et al. 2020).

In this paper we formally analyse an existing up-the-gradient model for establishment of polar auxin transport during leaf venation patterning (Jönsson et al. 2006; Heisler and Jonsson 2006; Merks et al. 2007). Although this model is a strong oversimplification of the experimental state-of-the-art, which in part invalidates it, it includes (1) auxin sensing, (2) polar transport, and (3) constitutive recycling, and thus likely contains key elements of updated, future models while still retaining the simplicity required for mathematical analysis. Thus, despite clear discrepancies of recent experimental insights with both the up-the-gradient and with-the-gradient models, the insights obtained in a formal analysis as well as the mathematical approaches developed in this work will likely apply to future, updated models of auxin-regulated patterning in plants.

### Mathematical motivation

In order to distinguish between the available phenomenological models of auxin-driven pattern formation and the general developmental principles that they represent, mathematical insight into the models’ structure and the models’ solutions will be crucial. This will help pinpoint key differences between the model structures and may uncover potential structural instabilities in the models upon which evolution may have acted, so as to produce new developmental patterning modules (Benítez et al. 2018). From the mathematical side, almost all previous studies have focused on the types of patterns that can be generated by different models once the transitory dynamics have died out. An important example is the study by Van Berkel and coworkers (van Berkel et al. 2013), where a number of models for polar auxin transport are recast into a common mathematical framework that allows them to be compared. A steady state analysis for a general class of active transport models can be found in Draelants et al. (2015), using advanced tools such as snaking from the field of bifurcation theory. Both periodic and stationary patterns are examined in Allen and Ptashnyk (2020), where the authors consider an extended with-the-gradient model. Haskovec and his coworkers derive local and global existence results together with an appropriate continuum limit for their graph-based diffusion model in Haskovec et al. (2019).

**Fig. 1:**
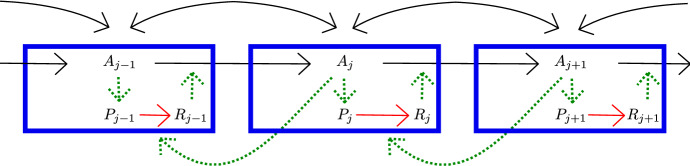
Schematic representation of the model (1.1). Black arrows represent transport, red arrows describe polarization and the green dashed arrows indication promotion. In particular, the PIN1 polarization rate correlates positively with the neighbouring auxin concentration, making this a model of ‘up-the-gradient’ type

Important qualitative examples of the up-the-gradient model are the formation of regularly spaced auxin maximums that lead to the growth of new leaves, as well as the formation of auxin channels that have been hypothesized to precede the formation of veins. Our goal here is to move beyond the well-studied equilibrium settings above and focus instead on understanding the dynamical behavior that leads to these patterns. In particular, we provide a rigorous framework to study a class of wave solutions that underpin the dynamical behaviour associated to up-the-gradient models. Ultimately, we hope that this analytic approach will provide an additional lens through which models of PAT can be examined and compared.

### The model

Inspired by Jönsson et al. (2006), Heisler and Jonsson (2006) and Merks et al. (2007), the system we will study is given by

1.1 $$\begin{aligned} {\left\{ \begin{array}{ll} {\dot{A}}_j = T_{{{\,\mathrm{act}\,}}}\left( R_{j-1}\frac{A_{j-1}}{k_a+A_{j-1}} - R_j\frac{A_j}{k_a+A_j}\right) + T_{{{\,\mathrm{diff}\,}}}(A_{j+1}-2A_j+A_{j-1}), \\ \\ {\dot{P}}_j = -k_1 \frac{A_{j+1}}{k_r + A_{j+1}}\left( \frac{P_j}{k_m+P_j}\right) + \alpha {A}_j , \\ \\ {\dot{R}}_j = k_1\frac{A_{j+1}}{k_r+A_{j+1}}\left( \frac{P_j}{k_m+P_j}\right) , \end{array}\right. } \end{aligned}$$

posed on the one-dimensional lattice $j \in \mathbb {Z}$; see Fig. 1. The variable $A_j(t)$ denotes the auxin concentration in cell $j \in \mathbb {Z}$, while $P_j(t)$ and $R_j(t)$ represent the unpolarized respectively right-polarized PIN1 in this cell. PIN1 is the PIN-variant that is believed to play a central role during auxin-based pattern formation in the shoot apical meristem and during leaf venation patterning (Reinhardt et al. 2003; Jönsson et al. 2006; Smith et al. 2006; Scarpella et al. 2006; Verna et al. 2019), and we therefore consider PIN1 here. However, note that the general structure of this model would apply to other polarised transporter proteins with similar behavior.

The parameters appearing in the problem are all strictly positive and labelled in the same manner as in Merks et al. (2007).^1^ In particular, $T_{\mathrm {act}}$ and $T_{\mathrm {diff}}$ denote the strengths of the active PIN1-induced rightward auxin transport and its diffusive counterpart, respectively. Unpolarized PIN1 is formed in the presence of auxin at a rate $\alpha$, while $k_1$ denotes the polarization rate. Finally, $k_a$, $k_r$, and $k_m$ are the Michaelis constants associated to the active transport of auxin and the polarization of PIN1, which depends on the auxin-concentration in the right-hand neighbouring cell. In particular, this model is of ‘up-the-gradient’ type.

The main difference compared to Merks et al. (2007) is that we are neglecting the presence of left-polarized PIN1 and have set the decay and depolarization rates of PIN1 to zero. Although this step of course imposes a pre-existing polarity on the system, we need to do this for technical reasons that we explain in the sequel. For now we simply point out that we wish to focus our attention on the dynamics of rightward auxin propagation, which takes place on timescales that are much faster than these decay and depolarization processes, and that the results will give novel insight into the full problem.

**Fig. 2:**
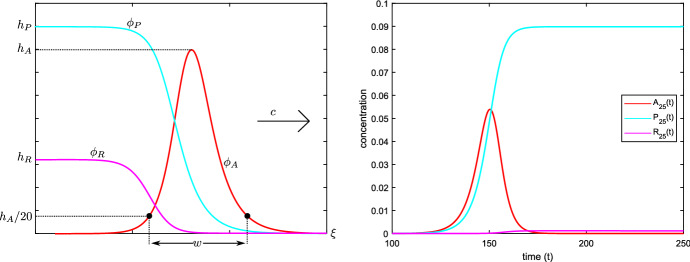
Left: cartoon of the waveprofiles $(\phi _A, \phi _P, \phi _R)$, illustrating the definition of the width *w* of the auxin-pulse and the limits (1.3). Right: numerical simulation of an auxin pulse passing through cell 25, leaving a residue of (polarized) PIN1. We used the procedure described in Sect. 1.4, with $A_1(0) = A_{\diamond } = 0.15$. The remaining parameters were fixed as $T_{\mathrm {act}} = 800$, $T_{\mathrm {diff}} = 0.15$, $k_a = 1$, $k_m = k_r = 100$, $k_1 = 200$ and $\alpha = 0.1$

We will look for solutions of the special type

1.2 $$\begin{aligned} (A_j, P_j, R_j)(t) = (\phi _A, \phi _P, \phi _R)(j - c t), \end{aligned}$$

with $c > 0$, in which we impose the limits

1.3 $$\begin{aligned} \lim _{\xi \rightarrow - \infty } \phi _A(\xi ) = 0, \qquad \qquad \lim _{\xi \rightarrow \infty } (\phi _A, \phi _P, \phi _R)(\xi ) = 0; \end{aligned}$$

see Fig. 2. From a modelling perspective, such solutions represent a pulse of auxin that moves to the right through a one-dimensional row of cells. Ahead of the wave the cells are clear of both polarized and unpolarized PIN, but behind the wavefront a residual amount of PIN is left in the cells, representing the coordinated polarisation of the tissue.

In reality these residues start to depolarize and decay, which can be included by adding linear decay terms to (1.1). This leads to the expanded system

1.4 $$\begin{aligned} {\left\{ \begin{array}{ll} {\dot{A}}_j = T_{{{\,\mathrm{act}\,}}}\left( R_{j-1}\frac{A_{j-1}}{k_a+A_{j-1}} - R_j\frac{A_j}{k_a+A_j}\right) + T_{{{\,\mathrm{diff}\,}}}(A_{j+1}-2A_j+A_{j-1}), \\ \\ {\dot{P}}_j = -k_1 \frac{A_{j+1}}{k_r + A_{j+1}}\left( \frac{P_j}{k_m+P_j}\right) +\alpha {A}_j + k_2 R_j - \delta P_j , \\ \\ {\dot{R}}_j = k_1\frac{A_{j+1}}{k_r+A_{j+1}}\left( \frac{P_j}{k_m+P_j}\right) - k_2 R_j, \end{array}\right. } \end{aligned}$$

in which the positive parameters $\delta$ and $k_2$ represent the decay and depolarization rate of PIN1, respectively. Mathematically, these terms can be included into our framework provided that the parameters $\delta$ and $k_2$ are small compared to the amplitude of the pulses, but we do not pursue this level of generality in the current paper for presentational clarity. Note in any case that in Merks et al. (2007) these parameters were chosen to be orders of magnitude smaller than $\alpha$ and $k_1$.

**Fig. 3:**
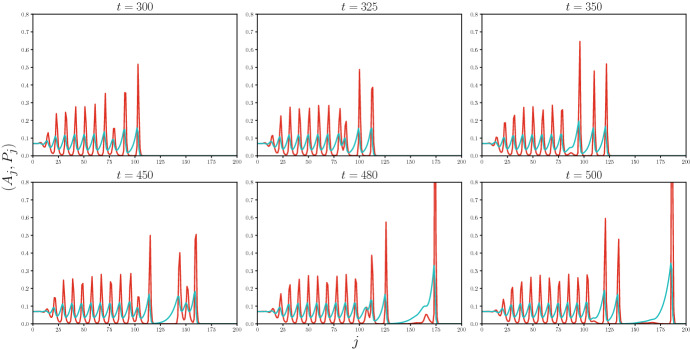
Six snapshots of a wavetrain simulation for the expanded system (1.4). Higher pulses travel faster than lower pulses, in correspondence with the scaling relations (1.7). These speed differences lead to merge events where even higher pulses are formed, which detach from the bulk. We used the procedure described in Sect. 1.4, taking $A_1(0) = A_{\diamond } = 0.0$ but adding 0.025 to ${\dot{A}}_1(t)$ to simulate a constant auxin influx at the left boundary. We picked $\delta = 0.1$ and $k_2 = 0.2$, leaving the remaining parameters from Fig. 2 unchanged. The full simulation can be found in supplementary video S1

Travelling waves have played a fundamental role in the analysis of many spatially discrete systems (Kevrekidis 2011; Mallet-Paret 1999; Chen et al. 2008; Hupkes and Sandstede 2010; Keener 1987). They can be seen as a lossless mechanism to transport matter or energy over arbitrary distances. As such, they are interesting in their own right, but they can also be viewed as building blocks to describe more complicated behaviour of nonlinear systems (Aronson and Weinberger 1975, 1978). In the present case for example, one can construct wavetrain solutions to (1.4) by adding a persistent auxin source; see Fig. 3 and Supplementary Video S1. Initially, these solutions can be seen in an approximate sense as a concatenation of the individual auxin pulses that we consider here (Moser 2021). As a consequence of the amplitude variations, small speed differences occur between these pulses which leads to highly interesting collision processes. Due to this type of versatility, travelling waves play an important role in many applications and have been extensively studied in a variety of settings (Sandstede 2002; Kevrekidis 2011; Hochstrasser et al. 1989; Jones et al. 1991).

### Main results

Our goal will be to obtain quantitative scaling information concerning the speed and shape of these waves. In particular, we will show rigorously that (1.1) admits a family of travelling wave solutions that are parameterized by the amplitude of the auxin-pulse. In addition, we show that the speed and width of these waves scale with this amplitude via a fractional power law. We state our results in full technical detail in Theorem 6.3 below.

**Fig. 4:**
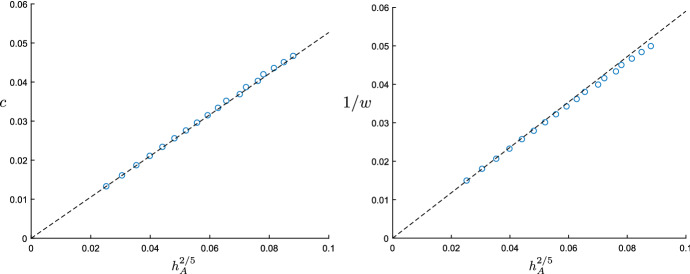
Scaling behaviour of the wavespeed *c* (left) and the auxin width *w* (right) against the height $h_A$ of the auxin pulse. The dashed lines represent the explicit predictions (1.7). The circles arise from numerical simulations, following the procedure described in Sect. 1.4 with several different values for $A_\diamond$. The other parameters were chosen as in Fig. 2

More precisely, we provide an explicit triplet of functions $(\phi _A^*, \phi _P^*, \phi _R^*)$ that satisfy the limits (1.3) and construct solutions to (1.1) of the form

1.5 $$\begin{aligned} \begin{array}{lcl} \big ( A_j, P_j, R_j \big )(t) &{}=&{} \Big ( \epsilon \phi _A^*, \epsilon ^{1/5} \phi _P^*, \epsilon ^{2/5} \phi _R^* \Big ) \Big ( \epsilon ^{2/5}( j - c_* \epsilon ^{2/5} t ) \Big ) \\ &{}&{} + \Big ( \mathcal {O}( \epsilon ^{17/15}), \mathcal {O}( \epsilon ^{1/3} ), \mathcal {O}( \epsilon ^{3/5} ) \Big ), \end{array} \end{aligned}$$

for a constant $c_*$, which we state exactly in (1.8). Here the limiting profile $\phi _A^*$ is scaled in such a way that $\Vert \phi _A^* \Vert _{L^{\infty }} = 1$. Upon introducing the heights^2^

1.6 $$\begin{aligned} (h_A, h_P, h_R) = \big ( \Vert A\Vert _\infty , \Vert P\Vert _\infty , \Vert R\Vert _\infty \big ) \end{aligned}$$

associated to the three components of our waves, this choice ensures that the auxin-height $h_A$ is equal to the parameter $\epsilon >0$ at leading order. In particular, comparing this to (1.2) we uncover the leading order scaling relations

1.7 $$\begin{aligned} c \sim c_* h_A^{2/5}, \qquad \qquad w \sim w_* h_A^{-2/5}, \qquad \qquad h_P \sim h_P^* h_A^{1/5}, \qquad \qquad h_R \sim h_R^* h_A^{2/5} \nonumber \\ \end{aligned}$$

for the speed *c*, width^3^*w* and heights of the wave. Here the constant $w_*$ denotes the width of the limiting profile $\phi _A^*$, while the other constants are given explicitly by

1.8 $$\begin{aligned} \begin{array}{lcl} c_* &{} = &{} \left( \frac{9\alpha {k}_1T_{{{\,\mathrm{act}\,}}}T_{{{\,\mathrm{diff}\,}}}^2}{8k_ak_mk_r}\right) ^{1/5}, \\ h_P^* &{} = &{} \sqrt{6} \left( \frac{9\alpha ^6 {k}_a^4 k_m^4 k_r^4 T_{{{\,\mathrm{diff}\,}}}^2}{8 k_1^4T_{{{\,\mathrm{act}\,}}}^4}\right) ^{1/10}, \\ h_R^* &{}= &{} 3\left( \frac{9\alpha k_a^4 {k}_1T_{{{\,\mathrm{diff}\,}}}^2}{8k_r k_m T_{{{\,\mathrm{act}\,}}}^4}\right) ^{1/5}. \end{array}\nonumber \\ \end{aligned}$$

In particular, for a fixed height of the auxin-pulse our results state that the speed and residual PIN1 will increase as the PIN1-production parameter $\alpha > 0$ is increased.

**Fig. 5:**
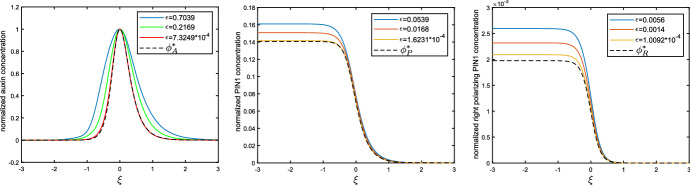
Convergence of the (scaled) profiles $\phi _A$ (left), $\phi _P$ (center) and $\phi _R$ (right) to their limits $(\phi _A^*, \phi _P^*, \phi _R^*)$. To perform the scalings, we wrote $h_A = \Vert \phi _A\Vert _{L^\infty }$, compressed space by a factor of $h_A^{2/5}$ and divided the three profiles by the respective factors $(h_A, h_A^{1/5}, h_A^{2/5})$, in line with the relations (1.7)

Although our proof requires the parameter $\epsilon > 0$ and hence the amplitude of the auxin-pulses to be small, this branch of solutions continues to exist well beyond this asymptotic regime. Indeed, we numerically confirmed the existence (and stability) of these waves by a direct simulation of (1.1) on a row of cells $j \in \{1 , \ldots 500 \}$, initialized with $A_j(0) = P_j(0) = R_j(0) =0$ for $2 \le j\le 500$, together with $P_1(0) = R_1(0)=0$ and $A_1(0) = A_{\diamond }$ for some $A_{\diamond } > 0$ that we varied between simulations. In order to close the system, we used the Neumann-type condition $A_0(t) = A_1(t)$ on the left-boundary, together with $R_0(t) = 0$ and a sink condition $A_{501}(t) = 0$ on the right. An example of such a simulation can be found in Fig. 2 (right). By varying the initial auxin concentration $A_{\diamond }$, we were able to generate waves with a range of amplitudes. We subsequently numerically computed the speed and width of these waves, which allowed us to confirm the leading order behaviour (1.7); see Fig. 4. In addition, we verified the convergence to the limiting profiles $(\phi _A^*,\phi _P^*, \phi _R^*)$ by comparing the appropriately rescaled numerical waveprofiles; see Fig. 5.

### Cross-diffusion

From a mathematical perspective, the problem (1.1) is interesting due to its interpretation as a so-called cross-diffusion problem, where the transport coefficient of one component is influenced by one of the other components. Work in this area was stimulated by developments in the modeling of bacterial cell membranes (Shih et al. 2019) and biofilms (Emerenini et al. 2015), where self-organization of biological molecules plays an important role. In the continuum regime, such problems are tough to analyze on account of potential degeneracies in the coefficients. The well-posedness of the underlying problem was analyzed in Sonner et al. (2011), while a numerical method for such problems was developed in Ghasemi et al. (2018).

The key phenomenological assumption behind such models is that particles behave differently when they are isolated compared to when they are part of a cluster. A simplified agent-based approach to capture this mechanism can be found in Johnston et al. (2017), which reduces naturally to a scalar PDE with nonlinear diffusion in the continuum limit. After adding a small regularization term, it is possible to use geometric singular perturbation theory to show that this PDE admits travelling wave solutions (Li et al. 2021). In this setting, the steepness of the wavefronts provides the necessary scale-separation required for rigorous results.

Our approach in this paper proceeds along entirely different lines, using the amplitude of the auxin pulse as a small continuation parameter to construct a family of travelling wave solutions to (1.1). The key insight is that one can extract an effective limiting system by scaling the width and speed of the wave in an appropriate fashion and sending the amplitude to zero. By means of a fixed-point analysis one can show in a rigorous fashion that solutions to this limiting system can be continued to form a family of solutions to the full system.

### Relation to FPUT pulses

Our technique is a generalization of the approach developed by Friesecke and Pego (1999) to construct small-amplitude travelling pulse solutions to the Fermi–Pasta–Ulam–Tsingou (FPUT) problem (Fermi et al. 1955; Dauxois 2008)

1.9 $$\begin{aligned} \ddot{x}_j = F(x_{j+1} - x_j) -F(x_{j} - x_{j-1}), \qquad \qquad j \in \mathbb {Z}. \end{aligned}$$

This models an infinite, one-dimensional chain of particles that can only move horizontally and are connected to their nearest neighbours by springs. These springs transmit a force

1.10 $$\begin{aligned} F(r) = r + r^2 \end{aligned}$$

that hence depends nonlinearly on the relative distance *r* between neighbouring particles; see Friesecke and Pego (1999), Herrmann and Matthies (2015) and Pankov (2005) for the impact of other choices. The FPUT system is well-established as a fundamental model to study the propagation of disturbances through spatially discrete systems, such as granular media, artificial metamaterials, DNA strands, and electrical transmission lines (Brillouin 1953; Kevrekidis 2011).

Looking for a travelling wave in the relative displacement coordinates, one introduces an Ansatz of the form

1.11 $$\begin{aligned} x_{j+1}(t) - x_j(t) = \phi (j - \sigma t ), \end{aligned}$$

which leads to the scalar functional differential equation of mixed type (MFDE)

1.12 $$\begin{aligned} \sigma ^2 \phi ''(\xi ) = F\big (\phi (\xi +1)\big ) - 2 F\big (\phi (\xi )\big ) + F\big (\phi (\xi - 1) \big ) . \end{aligned}$$

Following the classic papers by Friesecke in combination with Wattis (Friesecke and Wattis 1994) and Pego (Friesecke and Pego 1999, 2002, 2004a, b), we introduce the scaling

1.13 $$\begin{aligned} \phi (\xi ) = \epsilon ^2 \varphi _{\epsilon }(\epsilon \xi ) \end{aligned}$$

and write $\sigma = \sigma _{\epsilon }$, which transforms (1.12) into the MFDE

1.14 $$\begin{aligned} \sigma _{\epsilon }^2 \epsilon ^2 \varphi _{\epsilon }'' = \big ( S^{\epsilon } + S^{-\epsilon } - 2 \big ) \big [ \varphi _{\epsilon } + \epsilon ^2 \varphi _{\epsilon }^2 \big ] . \end{aligned}$$

Here the shift operator $S^d$ acts as

1.15 $$\begin{aligned} (S^d f)(\xi ) = f(\xi + d) \end{aligned}$$

for any $d \in \mathbb {R}$. Since the symbol $S^{\epsilon } + S^{-\epsilon }- 2$ represents a discrete Laplacian, we can interpret (1.14) as a wave equation with a nonlinear diffusion term. To some extent, this clarifies the link with our original problem (1.1) and the discussion above.

Applying the Fourier transform to (1.14) with *k* as the frequency variable, we arrive at

1.16 $$\begin{aligned} -\sigma _{\epsilon }^2 \epsilon ^2 k^2 {\widehat{\varphi }}_{\epsilon }(k)= & {} 2(\cos (\epsilon k) -1) \big [ \widehat{\varphi _{\epsilon }} + \epsilon ^2\widehat{\varphi _{\epsilon }^2} \big ](k) \nonumber \\= & {} - 4 \sin ^2(\epsilon k/2) \big [ \widehat{\varphi _{\epsilon }} + \epsilon ^2 \widehat{\varphi _{\epsilon }^2} \big ](k). \end{aligned}$$

Upon introducing the symbol

1.17 $$\begin{aligned} \widetilde{\mathcal {M}}_{\mathrm {FPUT}}^{(\epsilon )}(k) = \frac{4 \epsilon ^2 \sin ^2(\epsilon k/2)}{ \sigma _{\epsilon }^2 \epsilon ^2 k^2 - 4 \sin ^2(\epsilon k/2)}, \end{aligned}$$

this can be recast into the compact form

1.18 $$\begin{aligned} \widehat{\varphi _{\epsilon }}(k) = \widetilde{\mathcal {M}}_{\mathrm {FPUT}}^{(\epsilon )}(k) \widehat{\varphi _{\epsilon }^2}(k). \end{aligned}$$

Upon choosing the speed

1.19 $$\begin{aligned} \sigma _{\epsilon } = 1 + \frac{\epsilon ^2}{3}, \end{aligned}$$

we can exploit the expansion $\sin ^2(z/2) = \frac{1}{4} z^2 - \frac{1}{48} z^4 + O(z^6)$ to obtain the pointwise limit

1.20 $$\begin{aligned} \widetilde{\mathcal {M}}_{\mathrm {FPUT}}^{(\epsilon )}(k) \rightarrow \frac{12}{8 + k^2}, \qquad \qquad \epsilon \rightarrow 0 . \end{aligned}$$

Using the fact that $(8 + k^2)$ is the Fourier symbol for $8 - \partial ^2_{\xi }$, this suggests that the relevant system for $\varphi _{\epsilon }$ in the formal $\epsilon \rightarrow 0$ limit is given by

1.21 $$\begin{aligned} 8 \varphi _* - \varphi _*'' = 12 \varphi _*^2, \end{aligned}$$

which has the nontrivial even solution

1.22 $$\begin{aligned} \varphi _*(\xi ) = {{\,\mathrm{sech}\,}}^2(\sqrt{2} \xi ). \end{aligned}$$

By casting the problem in an appropriate functional analytic framework, one can show that this explicit solution $\varphi _*$ can be continued to yield solutions $\varphi _{\epsilon }$ to (1.14) for small $\epsilon > 0$. In this fashion, one establishes the existence of a family of pulse solutions (Friesecke and Pego 1999)

1.23 $$\begin{aligned} x_{j+1}(t) - x_j(t) = \epsilon ^2 {{\,\mathrm{sech}\,}}^2\Big (\sqrt{2} \epsilon (j - \sigma _{\epsilon } t) \Big ) + \mathcal {O} (\epsilon ^4). \end{aligned}$$

Roughly speaking, the main mathematical contribution in this paper is that we show how this analysis can be generalized to the setting of (1.1). The first main obstacle is that this is a multi-component system, which requires us to explicitly reduce the order before a tractable limit can be obtained. The second main obstacle is that the analysis of our Fourier symbol is considerably more delicate, since in our setting the wavespeed *c* converges to zero instead of one as $\epsilon \rightarrow 0$. Indeed, the denominator of $\widetilde{\mathcal {M}}_{\mathrm {FPUT}}^{(\epsilon )}$ above depends only on the product $\epsilon k$, while in our case there is a separate dependence on $\epsilon ^2 k$. This introduces a quasi-periodicity into the problem that requires our convergence analysis to carefully distinguish between ‘small’ values of *k* and several separate regions of ‘large’ *k*.

The third main difference is that we cannot use formal spectral arguments to analyze the limiting linear operator, which in our case is related to the Bernoulli equation. Instead, we apply a direct solution technique using variation-of-constants formulas. On the one hand this is much more explicit, but on the other hand the resulting estimates are rather delicate on account of the custom function spaces involved.

### Discussion

Due to the important organizing role that wave solutions often play in complex systems, scaling information such as (1.7) can be used as the starting point to uncover more general dynamical information concerning models such as (1.1) and related models of polar auxin trasnport. As such, we hope that the ideas we present here will provide a robust analytical tool to analyze different types of models as well. The resulting insights and predictions could help to prioritize competing models on the basis of dynamical experimental observations. Indeed, scaling laws appear to play a role in many aspects of biological systems, such as the structural properties of vascular systems (Razavi et al. 2018), the mass dependence of metabolic rates (West and Brown 2004) and the functional constraints imposed by size (Schmidt-Nielsen and Knut 1984).

Although we have included only right-polarizing PIN in our system, we believe that our techniques can be adapted to cover the full case where also left-polarizing PIN is included. However, the computations rapidly become unwieldy and the limiting system is expected to differ qualitatively. For this reason, we have not chosen to pursue this level of generality in the present paper, as it would only obscure the main ideas behind our framework. One of the main generalizations that we intend to pursue in the future is to study the model in two spatial dimensions. This is motivated by recent numerical observations concerning the formation of auxin channels and their associated PIN polarization under the influence of travelling patterns that are localized in both spatial dimensions (Althuis 2021; Merks et al. 2007).

### Notation

We summarize a few aspects of our (mostly standard) notation.

- If $f = f(X)$ is a differentiable function on $\mathbb {R}$, then we sometimes write $f' = \partial _X[f]$.
- If $\mathcal {X}$ and $\mathcal {Y}$ are normed spaces, then we denote the space of bounded linear operators from $\mathcal {X}$ to $\mathcal {Y}$ by $\mathbf {B}(\mathcal {X},\mathcal {Y})$. We put $\mathbf {B}(\mathcal {X}) := \mathbf {B}(\mathcal {X},\mathcal {X})$.
- We sometimes abbreviate $\mathbb {R}_+ := (0,\infty )$ and $\mathbb {R}_- := (-\infty ,0)$.

## The travelling wave problem

### Rewriting the original problem (1.1)

We will reduce the problem (1.1) to a system of equations involving only $A_j$ and $P_j$, and it will be this resulting system on which we make the long wave-scaled travelling wave Ansatz.

#### Changes of notation

We begin by rewriting (1.1) in a slightly more compressed manner that also exposes more transparently the leading order terms in the nonlinearities. Let $\delta ^{\pm }$ be the left and right difference operators that act on sequences $(x_j)$ in $\mathbb {R}$ via

$$\begin{aligned} \delta ^+x_j := x_{j+1}-x_j \qquad \text { and }\qquad \delta ^-x_j := x_j-x_{j-1}. \end{aligned}$$

Next, for *k*, $x \in \mathbb {R}$ with $k+x \ne 0$ we have

$$\begin{aligned} \frac{x}{k+x} = \frac{x}{k} - \frac{x^2}{k(k+x)}. \end{aligned}$$

We put

2.1 $$\begin{aligned} \mathsf {Q}_1(x,y) := \frac{x^2y}{k_a+x} \end{aligned}$$

and compress

2.2 $$\begin{aligned} \tau _1 := \frac{T_{{{\,\mathrm{act}\,}}}}{k_a} \qquad \text { and }\qquad \tau _2 := T_{{{\,\mathrm{diff}\,}}} \end{aligned}$$

to see that our equation for $A_j$ now reads

$$\begin{aligned} {\dot{A}}_j = \tau _2\delta ^+\delta ^-A_j - \tau _1\delta ^-(R_jA_j) + \tau _1\delta ^-\mathsf {Q}_1(A_j,R_j). \end{aligned}$$

Next, we abbreviate

2.3 $$\begin{aligned} \kappa := \frac{k_1}{k_rk_m} \end{aligned}$$

and put

2.4 $$\begin{aligned} \mathsf {Q}_2(x,y) := \kappa \left( \frac{k_ry + k_mx + xy}{(k_r+x)(k_m+y)}\right) xy \end{aligned}$$

to see that, the equation for $P_j$ is

$$\begin{aligned} {\dot{P}}_j = -\kappa {A}_{j+1}P_j + \alpha {A}_j + \mathsf {Q}_2(A_{j+1},P_j). \end{aligned}$$

The equation for $R_j$ is updated similarly, and so we have rewritten (1.1) as

2.5 $$\begin{aligned} {\left\{ \begin{array}{ll} {\dot{A}}_j = \tau _2\delta ^+\delta ^-A_j - \tau _1\delta ^-(R_jA_j) + \tau _1\delta ^-\mathsf {Q}_1(A_j,R_j), \\ \\ {\dot{P}}_j = -\kappa {A}_{j+1}P_j + \alpha {A}_j + \mathsf {Q}_2(A_{j+1},P_j), \\ \\ {\dot{R}}_j = \kappa {A}_{j+1}P_j - \mathsf {Q}_2(A_{j+1},P_j). \end{array}\right. } \end{aligned}$$

We observe that the equation for $R_j$ depends only on $A_{j+1}$ and $P_j$ and therefore can be solved by direct integration. Before we do that, however, we rewrite the new equation for $P_j$ using Duhamel’s formula.

#### Rewriting the $P_j$ equation

We can view the equation for $P_j$ in (2.5) as a first-order linear differential equation forced by $\alpha {A}_j + \mathsf {Q}_2(A_{j+1},P_j)$, and so we can solve it via the integrating factor method. For *f*, $g \in L^1$ and $h \in L^{\infty }$ we introduce the operators

2.6 $$\begin{aligned}&\mathsf {E}(f)(s,t) := \exp \left( -\kappa \int _s^t f(\xi ) \ d\xi \right) , \ s, t \in \mathbb {R}, \end{aligned}$$

2.7 $$\begin{aligned}&\mathsf {P}_1(f,g)(t) := \alpha \int _{-\infty }^t \mathsf {E}(f)(s,t)g(s) \ ds, \end{aligned}$$

and

2.8 $$\begin{aligned} \mathsf {P}_2(f,h)(t) := \int _{-\infty }^t \mathsf {E}(f)(s,t)\mathsf {Q}_2(f(s),h(s)) \ ds. \end{aligned}$$

Recall from (1.3) that we want $P_j$ to vanish at $-\infty$. The unique solution for $P_j$ in (2.5) that does vanish at $-\infty$ must satisfy

$$\begin{aligned} P_j(t) = \mathsf {P}_1(A_{j+1},A_j)(t) + \mathsf {P}_2(A_{j+1},P_j)(t). \end{aligned}$$

#### Solving the $R_j$ equation

Since, per (1.3), we want $R_j$ to vanish at $-\infty$, and since we are assuming that each $A_j$ vanishes sufficiently fast at both $\pm \infty$ and $P_j$ vanishes at $-\infty$ and remains bounded at $+\infty$, we may solve for $R_j$ by integrating the third equation in (2.5) from $-\infty$ to *t*. For *f*, $g \in L^1$ and $h \in L^{\infty }$, we define more integral operators:

2.9 $$\begin{aligned} \mathsf {R}_1(f,g)(t):= & {} \kappa \tau _1\int _{-\infty }^t f(s)\mathsf {P}_1(f,g)(s) \ ds, \ t \in \mathbb {R}, \end{aligned}$$

2.10 $$\begin{aligned} \mathsf {R}_2(f,g,h)(t):= & {} \int _{-\infty }^t \big (\kappa {f}(s)\mathsf {P}_2(f,g)(s)-\mathsf {Q}_2(f(s),\mathsf {P}_1(f,g)(s)\nonumber \\&+\mathsf {P}_2(f,h)(s))\big ) \ ds, \end{aligned}$$

and

2.11 $$\begin{aligned} \mathsf {R}(f,g,h)(t) := \mathsf {R}_1(f,g)(t) + \mathsf {R}_2(f,g,h)(t). \end{aligned}$$

We have defined $\mathsf {P}_1$ and $\mathsf {P}_2$ just above, respectively, in (2.7) and (2.8) and $\mathsf {Q}_2$ earlier in (2.4). Then the solution to the third equation in (2.5) that vanishes at $-\infty$ is

2.12 $$\begin{aligned} R_j(t) = \mathsf {R}(A_{j+1},A_j,P_j)(t) = \mathsf {R}_1(A_{j+1},A_j)(t) + \mathsf {R}_2(A_{j+1},A_j,P_j)(t). \end{aligned}$$

#### The final system for $A_j$ and $P_j$

We rewrite (part of) the $A_j$ equation once more to incorporate the new expression for $R_j$. For *f*, $g \in L^1$ and $h \in L^{\infty }$ and $t \in \mathbb {R}$ put

2.13 $$\begin{aligned} \mathsf {N}(f,g,h)(t) := \tau _1\mathsf {Q}_1(g(t),\mathsf {R}(f,g,h)(t)) - \tau _1\mathsf {R}_2(f,g,h)(t)g(t), \end{aligned}$$

where we defined $\mathsf {Q}_1$ in (2.1). Then $A_j$ must satisfy

$$\begin{aligned} {\dot{A}}_j = \tau _2\delta ^+\delta ^-A_j - \delta ^-\big (\mathsf {R}_1(A_{j+1},A_j)A_j\big ) + \delta ^-\mathsf {N}(A_{j+1},A_j,P_j), \end{aligned}$$

and so our system for $A_j$ and $P_j$ is now

2.14 $$\begin{aligned} {\left\{ \begin{array}{ll} {\dot{A}}_j = \tau _2\delta ^+\delta ^-A_j - \delta ^-\big (\mathsf {R}_1(A_{j+1},A_j)A_j\big ) + \delta ^-\mathsf {N}(A_{j+1},A_j,P_j), \\ \\ P_j = \mathsf {P}_1(A_{j+1},A_j) + \mathsf {P}_2(A_{j+1},P_j). \end{array}\right. } \end{aligned}$$

That is, using the formula (2.12) for $R_j$ in terms of $A_j$ and $P_j$, we can solve (2.5) if we can solve (2.14).

We will make two changes of variables on (2.14). First, in Sect. 2.2, we make a travelling wave Ansatz for $A_j$ and $P_j$. We reformulate (2.14) for the travelling wave profiles as the system (2.28) below. Then, in Sect. 3.1, we introduce our long wave scaling on these travelling wave profiles. After numerous adjustments, we arrive at the final system (3.14) for the scaled travelling wave profiles, which we solve in Sect. 6. The reader uninterested in these intermediate stages may wish to proceed directly to Proposition 3.5, which discusses the equivalence of the problem (2.14) for $A_j$ and $P_j$ and the ultimate long wave system (3.42). Of course, our notation must keep up with these changes of variables, and we summarize in Table 1 the evolution of a typical operator’s typesetting across these different problems.

**Table 1:** Summary of notational evolution

Symbol	Use
$\mathsf {R}$	The original problem (2.14)
$\widetilde{\mathsf {R}}^c$	The travelling wave problem (2.28)
$\breve{\mathsf {R}}^{\epsilon }$	The preliminary long wave problem (3.14)
$\mathcal {R}^{\nu }$	The final long wave problem (3.42)

##### Remark 2.1

The linearization of (2.14) at 0 yields

$$\begin{aligned} {\dot{A}}_j = \tau _2\delta ^+\delta ^-A_j, \qquad P_j = R_j = 0. \end{aligned}$$

If we follow the discussion after Friesecke and Pego (1999, Thm. 1.1), as well as Faver and Wright (2018, Rem. 2.2), and look for plane wave solutions $A_j(t) = e^{ikj-i\omega {t}}$ with $\omega$, $k \in \mathbb {R}$, we find the dispersion relation

2.15 $$\begin{aligned} -i\omega = 2\tau _2(\cos (k)-1). \end{aligned}$$

The only real solutions are $\omega =0$ and $k \in 2\pi \mathbb {Z}$. Previously, in Friesecke and Pego (1999) and Faver and Wright (2018) a nontrivial dispersion relation $\omega = \omega (k)$ was found by making the same kind of plane wave Ansatz, and the result ‘phase speed’ $k \mapsto \omega (k)/k$ had a nonzero maximum $c_s$, which was called the ‘speed of sound.’ These articles then proceeded to look for travelling waves with speed slightly above their respective values of $c_s$; these were ‘supersonic’ waves. For us, $\omega (k)$ is identically zero, which suggests that the speed of sound for our auxin problem is 0. Our long wave scaling in Sect. 3.1 analytically justifies this intuition.

### The travelling wave Ansatz

We now look for solutions $A_j$ and $P_j$ to (2.14) of the form

2.16 $$\begin{aligned} A_j = \phi _1(j-ct) \qquad \text { and }\qquad P_j = \phi _2(j-ct). \end{aligned}$$

The profiles $\phi _1$ and $\phi _2$ are real-valued functions of a single real variable and $c \in \mathbb {R}$. The following manipulations will be justified if we assume $\phi _1 \in H_q^1$ and $\phi _2 \in W^{1,\infty }$; we discuss the exponentially localized Sobolev space $H_q^1$ in Appendix A.3. Working on an exponentially localized space, as opposed to an algebraically weighted space, both allows us to capture precisely certain very fast decay properties and permits us to use some technical results on approximating Fourier multipliers. Furthermore, since we want $P_j$ to vanish at $-\infty$ and be asymptotically constant at $+\infty$, per the limits (1.3) and the numerical predictions of Fig. 2, we expect that $\phi _2$ should vanish at $+\infty$ and be asymptotically constant at $-\infty$.

We will convert the problem (2.14) for $A_j$ and $P_j$ into a nonlocal system for $\phi _1$ and $\phi _2$, with *c* as a parameter. Doing so amounts to little more than changing variables *many* times in the integral operators defined in Sects. 2.1.2 and 2.1.3 and gives us a host of new integral operators that will constitute the problem for $\phi _1$ and $\phi _2$.

In what follows we assume $f \in L^1$ and $g \in L^{\infty }$, so that the operators below are defined in the special cases of $f = \phi _1 \in H_q^1$ and $g = \phi _2 \in W^{1,\infty }$. First, for *x*, $v \in \mathbb {R}$, put

2.17 $$\begin{aligned} \widetilde{\mathsf {E}}^c(f)(v,x) := \exp \left( \frac{\kappa }{c}\int _v^x f(u+1) \ du\right) \end{aligned}$$

and

2.18 $$\begin{aligned} \widetilde{\mathsf {P}}_1^c(f)(x) := \frac{\alpha }{c}\int _x^{\infty } \widetilde{\mathsf {E}}^c(f)(v,x)f(v) \ dv. \end{aligned}$$

Then we use the Ansatz (2.16) and the definition of $\mathsf {P}_1$ in (2.7) to find

$$\begin{aligned} \mathsf {P}_1(A_{j+1},A_j)(t)= & {} \alpha \int _{-\infty }^t \exp \left( -\kappa \int _s^t \phi _1(j-c\xi +1)\ d\xi \right) \phi _1(j-cs) \ ds\\= & {} \widetilde{\mathsf {P}}_1^c(\phi _1)(j-ct). \end{aligned}$$

Here we have substituted $u = j-c\xi$ in the exponential’s integral and then $v = j-cs$ throughout.

Similar substitutions, which we do not discuss, yield the following identities. Put

2.19 $$\begin{aligned} \widetilde{\mathsf {P}}_2^c(f,g)(x) := \frac{1}{c}\int _x^{\infty } \widetilde{\mathsf {E}}^c(f)(v,x)\mathsf {Q}_2(f(v+1),g(v)) \ dv, \end{aligned}$$

so that with $\mathsf {P}_2$ defined in (2.8) we have

$$\begin{aligned} \mathsf {P}_2(A_{j+1},P_j)(t) = \widetilde{\mathsf {P}}_2^c(\phi _1,\phi _2)(j-ct). \end{aligned}$$

Thus $\phi _2$ must satisfy

2.20 $$\begin{aligned} \phi _2 = \widetilde{\mathsf {P}}_1^c(\phi _1) + \widetilde{\mathsf {P}}_2^c(\phi _1,\phi _2), \end{aligned}$$

which indicates that, as expected, $\phi _2$ should vanish at $+\infty$ and be asymptotically constant at $-\infty$.

Now we reformulate the equation for $A_j$, equivalently, for $\phi _1$. Put

2.21 $$\begin{aligned} \widetilde{\mathsf {R}}_1^c(f)(x) := \frac{\kappa \tau _1}{c}\int _x^{\infty } f(u+1)\widetilde{\mathsf {P}}_1^c(f)(u) \ du, \end{aligned}$$

so that with $\mathsf {R}_1$ defined in (2.9) we have

$$\begin{aligned} \mathsf {R}_1(A_{j+1},A_j)(t) = \widetilde{\mathsf {R}}_1^c(\phi _1)(j-ct). \end{aligned}$$

Put

2.22 $$\begin{aligned} \widetilde{\mathsf {R}}_2^c(f,g)(x):= & {} \frac{1}{c}\int _x^{\infty } \big (\kappa {f}(u+1)\widetilde{\mathsf {P}}_2^c(f,g)(u) \nonumber \\&- \mathsf {Q}_2(f(u+1),g(u))\big ) \ du\end{aligned}$$

and

2.23 $$\begin{aligned} \widetilde{\mathsf {R}}^c(f,g) := \widetilde{\mathsf {R}}_1^c(f) + \widetilde{\mathsf {R}}_2^c(f,g), \end{aligned}$$

so that with $\mathsf {R}_2$ defined in (2.10) and $\mathsf {R}$ in (2.11) we have

$$\begin{aligned}&\mathsf {R}_2(A_{j+1},A_j,P_j)(t) = \widetilde{\mathsf {R}}_2^c(\phi _1,\phi _2)(j-ct) \qquad \text { and }\qquad \\&\quad \mathsf {R}(A_{j+1},A_j,P_j)(t)= \widetilde{\mathsf {R}}^c(\phi _1,\phi _2)(j-ct). \end{aligned}$$

Last, put

2.24 $$\begin{aligned} \widetilde{\mathsf {N}}^c(f,g)(x) := \tau _1\widetilde{\mathsf {R}}_2^c(f,g)(x)f(x)-\tau _1\mathsf {Q}_1(f(x),\widetilde{\mathsf {R}}^c(f,g)(x)), \end{aligned}$$

so that with $\mathsf {N}$ defined in (2.13) we have

$$\begin{aligned} \mathsf {N}(A_{j+1},A_j,P_j)(t) = \widetilde{\mathsf {N}}^c(\phi _1,\phi _2)(j-ct). \end{aligned}$$

For a function $f :\mathbb {R}\rightarrow \mathbb {R}$ and $d \in \mathbb {R}$, define, as in (1.15), the shift operator $S^d$ by

2.25 $$\begin{aligned} (S^df)(x) := f(x+d). \end{aligned}$$

This final piece of notation, along with the Eq. (2.20), allows us to convert the problem (2.14) for $A_j$ and $P_j$ into the following nonlocal system for $\phi _1$ and $\phi _2$:

2.26 $$\begin{aligned} {\left\{ \begin{array}{ll} -c\phi _1' = \tau _2(S^1-2+S^{-1})\phi _1 + (S^{-1}-1)\big (\widetilde{\mathsf {R}}_1^c(\phi _1)\phi _1+ \widetilde{\mathsf {N}}^c(\phi _1,\phi _2)\big ), \\ \\ \phi _2 = \widetilde{\mathsf {P}}_1^c(\phi _1) + \widetilde{\mathsf {P}}_2^c(\phi _1,\phi _2). \end{array}\right. } \end{aligned}$$

### The Fourier multiplier structure

We summarize our conventions and definitions for Fourier transforms and Fourier multipliers in Appendix A. If we take the Fourier transform of the equation for $\phi _1$ in (2.26), we find

$$\begin{aligned} \big (ick + 2\tau _2(\cos (k)-1)\big ){\widehat{\phi }}_1(k) = (1-e^{-ik})\mathfrak {F}\big [\widetilde{\mathsf {R}}_1^c(\phi _1)\phi _1+ \widetilde{\mathsf {N}}^c(\phi _1,\phi _2)\big ](k). \end{aligned}$$

For $k \in \mathbb {R}$, we have $ick+2\tau _2(\cos (k)-1) = 0$ if and only if $k=0$. Consequently, the function

2.27 $$\begin{aligned} \widetilde{\mathsf {M}}_c(k) := \frac{1-e^{-ik}}{ick+2\tau _2(\cos (k)-1)} \end{aligned}$$

has a removable singularity at 0 and is in fact analytic on $\mathbb {R}$. We therefore define $\mathsf {M}_c$ to be the Fourier multiplier with symbol $\widetilde{\mathsf {M}}_c$, i.e., $\mathsf {M}_c$ satisfies

$$\begin{aligned} \widehat{\mathsf {M}_cf}(k) = \widetilde{\mathsf {M}}_c(k){\widehat{f}}(k). \end{aligned}$$

We discuss some further properties of Fourier multipliers in Appendix A.2. Now the problem (2.26) is equivalent to

2.28 $$\begin{aligned} {\left\{ \begin{array}{ll} \phi _1 = \mathsf {M}_c\big (\widetilde{\mathsf {R}}_1^c(\phi _1)\phi _1 + \widetilde{\mathsf {N}}^c(\phi _1,\phi _2)\big ) \\ \phi _2 = \widetilde{\mathsf {P}}_1^c(\phi _1) + \widetilde{\mathsf {P}}_2^c(\phi _1,\phi _2). \end{array}\right. } \end{aligned}$$

## The long wave problem

### The long wave scaling

We now make the long wave Ansatz

3.1 $$\begin{aligned} \phi _1(x) = \epsilon \psi _1(\epsilon ^{\mu }x), \qquad \phi _2(x) = \epsilon ^{\beta }\psi _2(\epsilon ^{\mu }x), \qquad \text { and }\qquad c = \epsilon ^{\gamma }c_0. \end{aligned}$$

We assume, as with $\phi _1$ and $\phi _2$, that the scaled profiles satisfy $\psi _1 \in H_q^1$ and $\psi _2 \in W^{1,\infty }$. We think of $\epsilon > 0$ as small and keep the exponents $\beta$, $\gamma$, $\mu > 0$ arbitrary for now; eventually we will pick

$$\begin{aligned} \gamma = \mu = \frac{2}{5} \qquad \text { and }\qquad \beta = \frac{1}{5}. \end{aligned}$$

The reasoning behind this choice is by no means obvious at this point and will not be for some time; leaving $\mu$, $\beta$, and $\gamma$ arbitrary will allow this choice to appear more naturally (at the cost of temporarily more cumbersome notation).

As we intuited in Remark 2.1, our wave speed is now close to 0, which is the auxin problem’s natural ‘speed of sound’. The parameter $c_0 \ne 0$ affords us some additional flexibility in choosing the wave speed. A properly chosen value of $c_0$ will cause the maximum of the leading-order term of $\phi _1$ to be $\epsilon$, which will fulfill our promise in Sect. 1.4 that the auxin-height is, to leading order, $\epsilon$. Friesecke and Pego introduce a similar auxiliary parameter into their $\epsilon$-dependent wave speed, see Friesecke and Pego (1999, Eq. (2.5), (2.13)). This parameter allows them to prove that the dependence of their travelling wave profile on wave speed is sufficiently regular in different function spaces, a result needed for their subsequent stability arguments in Friesecke and Pego (2002, 2004a, 2004b). We did not provide this extra parameter in our version (1.19) of the Friesecke–Pego wave speed, but rather we selected it so that the amplitude of the leading order ${{\,\mathrm{sech}\,}}^2$-profile term in (1.22) is 1. Similarly, we will not pursue their depth of wave-speed analysis on our profiles’ dependence on $c_0$.

We convert (2.28) to another nonlocal system for $\psi _1$ and $\psi _2$, which now depends heavily on the parameter $\epsilon$. As before, this process mostly amounts to changing variables in many integrals. For example, we use the definition of $\widetilde{\mathsf {P}}_1^c$ in (2.18) and the Ansatz (3.1) to find

3.2 $$\begin{aligned} \widetilde{\mathsf {P}}_1^c(\phi _1)(x) = \frac{\alpha }{\epsilon ^{\gamma }c_0}\int _x^{\infty } \widetilde{\mathsf {E}}^c(\phi _1)(v,x)\epsilon \psi _1(\epsilon ^{\mu }v) \ dv, \end{aligned}$$

where, using the definition of $\widetilde{\mathsf {E}}^c$ in (2.17), we have

$$\begin{aligned} \widetilde{\mathsf {E}}^c(\phi _1)(v,x)= & {} \exp \left( \frac{\kappa }{\epsilon ^{\gamma }c_0}\int _v^x \epsilon \psi _1(\epsilon ^{\mu }u + \epsilon ^{\mu }) \ du\right) \\= & {} \exp \left( \frac{\kappa }{c_0}\epsilon ^{1-(\gamma +\mu )}\int _{\epsilon ^{\mu }v}^{\epsilon ^{\mu }x} \psi _1(U + \epsilon ^{\mu }) \ dU\right) . \end{aligned}$$

Here we have substituted $U = \epsilon ^{\mu }u$.

Now for $f \in L^1$ we put

3.3 $$\begin{aligned} \mathcal {E}(f)(V,X) := \exp \left( \frac{\kappa }{c_0}\int _V^X f(U) \ dU\right) , \ V, X \in \mathbb {R}, \end{aligned}$$

so that (3.2) becomes

$$\begin{aligned} \widetilde{\mathsf {P}}_1^c(\phi _1)(x) = \frac{\alpha }{c_0}\epsilon ^{1-\gamma }\int _x^{\infty } \mathcal {E}(\epsilon ^{1-(\gamma +\mu )}S^{\epsilon ^{\mu }}\psi _1)(\epsilon ^{\mu }v,\epsilon ^{\mu }x)\psi _1(\epsilon ^{\mu }v) \ dv. \end{aligned}$$

Here $S^{\epsilon ^{\mu }}$ is the shift operator defined in (2.25) with $d = \epsilon ^{\mu }$. We substitute again with $V = \epsilon ^{\mu }v$ and define

3.4 $$\begin{aligned} \breve{\mathsf {P}}_1^{\epsilon }(f)(X) := \frac{\alpha }{c_0}\int _X^{\infty } \mathcal {E}(\epsilon ^{1-(\gamma +\mu )}S^{\epsilon ^{\mu }}f)(V,X)f(V)\ dV\end{aligned}$$

to conclude that

$$\begin{aligned} \widetilde{\mathsf {P}}_1^c(\phi _1)(x) = \epsilon ^{1-(\gamma +\mu )}\breve{\mathsf {P}}_1^{\epsilon }(\psi _1)(\epsilon ^{\mu }x). \end{aligned}$$

Similar careful substitutions will allow us to reformulate the integral operators from Sect. 2.2 in terms of the long wave Ansatz. First, however, we define

3.5 $$\begin{aligned} \breve{\mathsf {Q}}_1^{\epsilon }(X,Y) := \frac{X^2Y}{k_a+\epsilon {X}} \qquad \text { and }\qquad \breve{\mathsf {Q}}_2^{\epsilon }(X,Y) := \kappa \frac{k_rY+k_m\epsilon ^{1-\beta }X+\epsilon {XY}}{(k_r+\epsilon {X})(k_m+\epsilon ^{\beta }Y)}XY . \nonumber \\ \end{aligned}$$

When $\epsilon \ne 0$, this definition permits the very convenient factorizations

$$\begin{aligned} \mathsf {Q}_1(\epsilon {X},\epsilon ^{1-(\gamma +\mu )}Y) = \epsilon ^{3-(\gamma +\mu )}\breve{\mathsf {Q}}_1^{\epsilon }(X,Y) \qquad \text { and }\qquad \mathsf {Q}_2(\epsilon {X},\epsilon ^{\beta }Y) = \epsilon ^{1+2\beta }\breve{\mathsf {Q}}_2^{\epsilon }(X,Y), \end{aligned}$$

where $\mathsf {Q}_1$ was defined in (2.1) and $\mathsf {Q}_2$ in (2.4).

Now we work on the travelling wave integral operators. Below we will assume $f \in L^1$ and $g \in L^{\infty }$. Put

3.6 $$\begin{aligned} \breve{\mathsf {P}}_2^{\epsilon }(f,g)(X) := \frac{1}{c_0}\int _X^{\infty } \mathcal {E}(\epsilon ^{1-(\gamma +\mu )}S^{\epsilon ^{\mu }}f)(V,X)\breve{\mathsf {Q}}_2^{\epsilon }(f(V+\epsilon ^{\mu }),g(V)) \ dV, \nonumber \\ \end{aligned}$$

so that with $\widetilde{\mathsf {P}}_2^c$ defined in (2.19) we have

$$\begin{aligned} \widetilde{\mathsf {P}}_2^c(\phi _1,\phi _2)(x) = \epsilon ^{1-(\gamma +\mu )+2\beta }\breve{\mathsf {P}}_2^{\epsilon }(\psi _1,\psi _2)(\epsilon ^{\mu }x). \end{aligned}$$

This converts the second equation in (2.28) for $\phi _2$ to

$$\begin{aligned} \epsilon ^{\beta }\psi _2(\epsilon ^{\mu }x) = \epsilon ^{1-(\gamma +\mu )}\breve{\mathsf {P}}_1^{\epsilon }(\psi _1)(\epsilon ^{\mu }x) + \epsilon ^{1-(\gamma +\mu )+2\beta }\breve{\mathsf {P}}_2^{\epsilon }(\psi _1,\psi _2)(\epsilon ^{\mu }x). \end{aligned}$$

Passing to $X = \epsilon ^{\mu }x$, we find that $\psi _2$ must satisfy

3.7 $$\begin{aligned} \psi _2(X) = \epsilon ^{1-(\gamma +\mu )-\beta }\breve{\mathsf {P}}_1^{\epsilon }(\psi _1)(X) + \epsilon ^{1-(\gamma +\mu )+\beta }\breve{\mathsf {P}}_2^{\epsilon }(\psi _1,\psi _2)(X). \end{aligned}$$

Now put

3.8 $$\begin{aligned} \breve{\mathsf {R}}_1^{\epsilon }(f)(X) := \frac{\kappa \tau _1}{c_0}\int _X^{\infty } \breve{\mathsf {P}}_1^{\epsilon }(f)(V)f(V + \epsilon ^{\mu }) \ dV, \end{aligned}$$

so that with $\widetilde{\mathsf {R}}_1^c$ defined in (2.21) we have

$$\begin{aligned} \widetilde{\mathsf {R}}_1^c(\phi _1)(x) = \epsilon ^{2(1-(\gamma +\mu ))}\breve{\mathsf {R}}_1^{\epsilon }(\psi _1)(\epsilon ^{\mu }x). \end{aligned}$$

Put

3.9 $$\begin{aligned} \breve{\mathsf {R}}_2^{\epsilon }(f,g)(X) := & {} \frac{1}{c_0}\int _X^{\infty } \big (\epsilon ^{1-(\gamma +\mu )}\kappa {f}(V+\epsilon ^{\mu })\breve{\mathsf {P}}_2^{\epsilon }(f,g)(V)\nonumber \\&\quad -\breve{\mathsf {Q}}_2^{\epsilon }(f(V+\epsilon ^{\mu }),g(V))\big ) \ dV\end{aligned}$$

and

3.10 $$\begin{aligned} \breve{\mathsf {R}}^{\epsilon }(f,g)(X) := \epsilon ^{1-(\gamma +\mu )}\breve{\mathsf {R}}_1^{\epsilon }(f)(X) + \epsilon ^{2\beta }\mathsf {R}_2^{\epsilon }(f,g)(X), \end{aligned}$$

so that with $\widetilde{\mathsf {R}}_2^c$ defined in (2.22) and $\widetilde{\mathsf {R}}^c$ defined in (2.23) we have

$$\begin{aligned}&\widetilde{\mathsf {R}}_2^c(\phi _1,\phi _2)(x) = \epsilon ^{1-(\gamma +\mu )+2\beta }\breve{\mathsf {R}}_2^{\epsilon }(\psi _1,\psi _2)(\epsilon ^{\mu }x) \qquad \text { and }\qquad \\&\quad \widetilde{\mathsf {R}}^c(\phi _1,\phi _2)(x) = \epsilon ^{1-(\gamma +\mu )}\breve{\mathsf {R}}^{\epsilon }(\psi _1,\psi _2)(\epsilon ^{\mu }x). \end{aligned}$$

Finally, put

3.11 $$\begin{aligned} \breve{\mathsf {N}}^{\epsilon }(f,g)(X) := \tau _1\breve{\mathsf {R}}_2^{\epsilon }(f,g)(X)f(X) - \epsilon ^{1-2\beta }\tau _1\breve{\mathsf {Q}}_1^{\epsilon }(f(X),\breve{\mathsf {R}}^{\epsilon }(f,g)(X)), \end{aligned}$$

so that with $\widetilde{\mathsf {N}}^c$ defined in (2.24) we have

$$\begin{aligned} \widetilde{\mathsf {N}}^c(\phi _1,\phi _2)(x) = \epsilon ^{2-(\gamma +\mu )+2\beta }\breve{\mathsf {N}}^{\epsilon }(\psi _1,\psi _2)(\epsilon ^{\mu }x). \end{aligned}$$

The definition of scaled Fourier multipliers from (A.3) tells us that, for $\epsilon > 0$, $\mathsf {M}_{\epsilon ^{\gamma }c_0}^{(\epsilon ^{\mu })}$ is the Fourier multiplier satisfying

$$\begin{aligned} \widehat{\mathsf {M}_{\epsilon ^{\gamma }c_0}^{(\epsilon ^{\mu })}f}(k) = \widetilde{\mathsf {M}}_{\epsilon ^{\gamma }c_0}(\epsilon ^{\mu }k){\widehat{f}}(k), \end{aligned}$$

where $\widetilde{\mathsf {M}}_{\epsilon ^{\gamma }c_0}$ is defined by taking $c = \epsilon ^{\gamma }c_0$ in (2.27). This converts the first equation in (2.28) for $\phi _1$ to

$$\begin{aligned} \epsilon \psi _1(\epsilon ^{\mu }x) = \mathsf {M}_{\epsilon ^{\gamma }c_0}^{(\epsilon ^{\mu })}[\epsilon ^{2(1-(\gamma +\mu ))}\breve{\mathsf {R}}_1^{\epsilon }(\psi _1)\epsilon \psi _1 + \epsilon ^{2-(\gamma +\mu )+2\beta }\breve{\mathsf {N}}^{\epsilon }(\psi _1,\psi _2)](\epsilon ^{\mu }x). \end{aligned}$$

We factor this to reveal

3.12 $$\begin{aligned} \psi _1(X) = \epsilon ^{2(1-(\gamma +\mu ))}\mathsf {M}_{\epsilon ^{\gamma }c_0}^{(\epsilon ^{\mu })}\big [\breve{\mathsf {R}}_1^{\epsilon }(\psi _1)\psi _1 + \epsilon ^{-1+\gamma +\mu +2\beta }\mathsf {N}^{\epsilon }(\psi _1,\psi _2)\big ](X). \end{aligned}$$

We abbreviate

3.13 $$\begin{aligned} \breve{\mathsf {M}}_{\epsilon } := \epsilon ^{2(1-(\gamma +\mu ))}\mathsf {M}_{\epsilon ^{\gamma }c_0}^{(\epsilon ^{\mu })} \end{aligned}$$

to conclude from (3.12) and the prior Eq. (3.7) for $\psi _2$ that the long wave profiles must satisfy

3.14 $$\begin{aligned} {\left\{ \begin{array}{ll} \psi _1 = \breve{\mathsf {M}}_{\epsilon }\big [\breve{\mathsf {R}}_1^{\epsilon }(\psi _1)\psi _1 + \epsilon ^{-1+\gamma +\mu +2\beta }\breve{\mathsf {N}}^{\epsilon }(\psi _1,\psi _2)\big ] \\ \\ \psi _2 = \epsilon ^{1-(\gamma +\mu +\beta )}\breve{\mathsf {P}}_1^{\epsilon }(\psi _1) + \epsilon ^{1-(\gamma +\mu )+\beta }\breve{\mathsf {P}}_2^{\epsilon }(\psi _1,\psi _2). \end{array}\right. } \end{aligned}$$

We have been tacitly assuming that all of the exponents on powers of $\epsilon$ above are nonnegative so that the various $\epsilon$-dependent operators and prefactors are actually defined at $\epsilon =0$. In particular, this demands

3.15 $$\begin{aligned} 1-2\beta \ge 0, \qquad -1+\gamma +\mu +2\beta \ge 0, \qquad \text { and }\qquad 1-(\gamma +\mu +\beta ) \ge 0. \nonumber \\ \end{aligned}$$

### The formal long wave limit and exponent selection

Our intention is now to take the limit $\epsilon \rightarrow 0$ in the Eq. (3.14) for $\psi _1$ and $\psi _2$. Doing so in a way that the limit is both meaningful (i.e., defined and nontrivial) and reflective of what the numerics predict at $\epsilon =0$ will teach us what the exponents $\mu$, $\gamma$, and $\beta$ should be, beyond the requirements of (3.15).

#### The formal limit on $\breve{\mathsf {M}}_{\epsilon }$ and the selection of the exponents $\gamma$ and $\mu$

We want to assign a ‘natural’ definition to $\breve{\mathsf {M}}_0$, where $\breve{\mathsf {M}}_{\epsilon }$ was defined, for $\epsilon > 0$, in (3.13). However, we relied above on having $\epsilon > 0$ to invoke the scaled Fourier multiplier identity (A.3) that gave us $\breve{\mathsf {M}}_{\epsilon }$, and naively setting $\epsilon = 0$ in that identity is meaningless. Additionally, we should be careful that the prefactor $\epsilon ^{2(1-(\gamma +\mu ))}$ in (3.13) does not lead us to define $\breve{\mathsf {M}}_0 = 0$; otherwise, we would have $\psi _1 = 0$ when $\epsilon = 0$, and that is not what the numerics in Fig. 2 predict.

A natural starting point, then, is to study $\breve{\mathsf {M}}_{\epsilon }$ in the limit $\epsilon \rightarrow 0^+$, and this amounts to considering the limit of its symbol, whose definition we extract from the definition of $\breve{\mathsf {M}}_{\epsilon }$ in (3.13) and the definition of the scaled Fourier multiplier in (A.3). Thus, for each $k \in \mathbb {R}$, we want the limit

3.16 $$\begin{aligned} \lim _{\epsilon \rightarrow 0^+} \epsilon ^{2(1-(\gamma +\mu ))}\widetilde{\mathsf {M}}_{\epsilon ^{\gamma }c_0}(\epsilon ^{\mu }k) \end{aligned}$$

to exist without being identically zero. The function $\widetilde{\mathsf {M}}_{\epsilon ^{\gamma }c_0}$ was defined in (2.27).

To calculate this limit, we first state the Taylor expansions

3.17 $$\begin{aligned} 1-e^{-iz} = iz+iz^2N_1(z) \qquad \text { and }\qquad \cos (z)-1 = -\frac{z^2}{2}+ \frac{iz^4N_2(z)}{2\tau _2} \end{aligned}$$

for $z \in \mathbb {C}$. The functions $N_1$ and $N_2$ are analytic and uniformly bounded on strips in the sense that

3.18 $$\begin{aligned} C_q := \sup _{x \in \mathbb {R}} |N_1(x\pm {iq})| + |N_2(x\pm {iq})| < \infty \end{aligned}$$

for any $q > 0$. The choice of constants on $N_1$ and $N_2$ will permit some useful cancellations later. Then

$$\begin{aligned} \widetilde{\mathsf {M}}_c(k) = \frac{ik+ik^2N_1(k)}{ick -\tau _2k^2 + ik^4N_2(k)} = \frac{1+kN_1(k)}{c+i\tau _2k + k^3N_2(k)}, \end{aligned}$$

and so

3.19 $$\begin{aligned} \epsilon ^{2(1-(\gamma +\mu ))}\widetilde{\mathsf {M}}_{\epsilon ^{\gamma }c_0}(\epsilon ^{\mu }k) = \epsilon ^{2(1-(\gamma +\mu ))}\frac{1+\epsilon ^{\mu }kN_1(\epsilon ^{\mu }k)}{\epsilon ^{\gamma }c_0 + i\tau _2\epsilon ^{\mu }k + \epsilon ^{3\mu }k^3N_2(\epsilon ^{\mu }k)}. \quad \end{aligned}$$

At this point it does not make sense to set $\epsilon = 0$, as then the denominator would be identically zero. So, we would like to factor some power of $\epsilon$ out of the denominator. Since the first term in the denominator has a factor of $\epsilon ^{\gamma }$ and the second a factor of $\epsilon ^{\mu }$, we assume $\gamma = \mu$ and remove the power of $\epsilon$ from both the first and the second terms. We discuss the choice of $\gamma =\mu$ further in Remark 3.2.

Then

3.20 $$\begin{aligned} \epsilon ^{2(1-(\gamma +\mu ))}\widetilde{\mathsf {M}}_{\epsilon ^{\gamma }c_0}(\epsilon ^{\mu }k)= & {} \epsilon ^{2(1-2\gamma )}\widetilde{\mathsf {M}}_{\epsilon ^{\gamma }c_0}(\epsilon ^{\gamma }k) \nonumber \\= & {} \epsilon ^{2(1-2\gamma )-\gamma }\frac{1+\epsilon ^{\gamma }kN_1(\epsilon ^{\gamma }k)}{c_0+i\tau _2k+\epsilon ^{2\gamma }k^3N_2(\epsilon ^{\gamma }k)}. \end{aligned}$$

Pointwise in *k* we have

$$\begin{aligned} \lim _{\epsilon \rightarrow 0^+} \frac{1+\epsilon ^{\gamma }kN_1(\epsilon ^{\gamma }k)}{c_0+i\tau _2k+\epsilon ^{2\gamma }k^3N_2(\epsilon ^{\gamma }k)} = \frac{1}{c_0+i\tau _2k}, \end{aligned}$$

and so we want

$$\begin{aligned} 2(1-2\gamma )-\gamma = 0 \end{aligned}$$

so that the prefactor of $\epsilon ^{2(1-2\gamma )-\gamma }$ in (3.20) does not induce a trivial or undefined limit. Thus we take

$$\begin{aligned} \gamma = \mu = \frac{2}{5}. \end{aligned}$$

Certainly doing so does not contradict any of the inequalities in (3.15), provided that $\beta$ is chosen appropriately. Moreover, the power of 2/5 agrees with the height-speed-width relations suggested in Fig. 4. And so

$$\begin{aligned} \lim _{\epsilon \rightarrow 0^+} \epsilon ^{2(1-(\gamma +\mu ))}\widetilde{\mathsf {M}}_{\epsilon ^{\gamma }c_0}(\epsilon ^{\mu }k) = \lim _{\epsilon \rightarrow 0^+} \epsilon ^{4/5}\widetilde{\mathsf {M}}_{\epsilon ^{2/5}c_0}(\epsilon ^{2/5}k) = \frac{1}{c_0+\tau _2ik}. \end{aligned}$$

Put

3.21 $$\begin{aligned} \widetilde{\mathcal {M}}^{(0)}(z) := \frac{1}{c_0+i\tau _2z}, \end{aligned}$$

so $\widetilde{\mathcal {M}}^{(0)}$ is analytic on any strip $\left\{ z \in \mathbb {C} \ | \ |{{\,\mathrm{Im}\,}}(z)| < q\right\}$ for $q \in (0,\tau _2/c_0)$. Let $\mathcal {M}^{(0)}$ be the Fourier multiplier with symbol $\widetilde{\mathcal {M}}^{(0)}$.

Lemma A.2 then gives the following properties of $\mathcal {M}^{(0)}$; the identities (3.22) are direct calculations with the Fourier transform.

##### Lemma 3.1

Fix $q \in (0,\tau _2/c_0)$. Then $\mathcal {M}^{(0)} \in \mathbf {B}(H_q^r,H_q^{r+1})$ for all *r*. More generally, if $f \in H^1$ and $g \in L^2$, then

3.22 $$\begin{aligned} \mathcal {M}^{(0)}(c_0+ \tau _2\partial _X)f = f \qquad \text { and }\qquad (c_0+\tau _2\partial _X)\mathcal {M}^{(0)}g = g. \end{aligned}$$

Because of the identities (3.22), we write $\mathcal {M}^{(0)} = (c_0+\tau _2\partial _X)^{-1}$. The formal analysis above then leads us to expect

3.23 $$\begin{aligned} \lim _{\epsilon \rightarrow 0^+} \breve{\mathsf {M}}_{\epsilon } = \mathcal {M}^{(0)} = (c_0+\tau _2\partial _X)^{-1}. \end{aligned}$$

However, we have not yet proved this rigorously by any means.

##### Remark 3.2

Here is why we take $\gamma =\mu$ when factoring the power of $\epsilon$ out of the denominator in (3.19). First, taking $\gamma > \mu$ produces

$$\begin{aligned} \epsilon ^{2(1-(\gamma +\mu ))}\widetilde{\mathsf {M}}_{\epsilon ^{\gamma }c_0}(\epsilon ^{\mu }k) = \epsilon ^{2(1-(\gamma +\mu ))-\mu }\frac{1+\epsilon ^{\mu }kN_1(\epsilon ^{\mu }k)}{\epsilon ^{\gamma -\mu }c_0 + i\tau _2k + \epsilon ^{2\mu }k^3N_2(\epsilon ^{\mu }k)} \end{aligned}$$

instead of (3.20). If $2(1-(\gamma +\mu ))-\mu > 0$, then the right side above is identically zero at $\epsilon = 0$, and so we demand $2(1-(\gamma +\mu ))-\mu = 0$; there are many pairs of $\gamma$ and $\mu$ that work here. But then

$$\begin{aligned} \lim _{\epsilon \rightarrow 0^+} \frac{1+\epsilon ^{\mu }kN_1(\epsilon ^{\mu }k)}{\epsilon ^{\gamma -\mu }c_0 + i\tau _2k + \epsilon ^{2\mu }k^3N_2(\epsilon ^{\mu }k)} = \frac{1}{i\tau _2k}. \end{aligned}$$

This suggests that instead of (3.23), we have

$$\begin{aligned} \lim _{\epsilon \rightarrow 0^+} \breve{\mathsf {M}}_{\epsilon } = (\tau _2\partial _X)^{-1}. \end{aligned}$$

However, this is meaningless: differentiation is not invertible from $H_q^r$ to $H_q^{r+1}$.

Taking $\gamma < \mu$ also does not work. In that case, instead of (3.20) we would have found

$$\begin{aligned} \epsilon ^{2(1-(\gamma +\mu ))}\widetilde{\mathsf {M}}_{\epsilon ^{\gamma }c_0}(\epsilon ^{\mu }k) = \epsilon ^{2(1-(\gamma +\mu ))-\gamma }\frac{1+\epsilon ^{\mu }kN_1(\epsilon ^{\mu }k)}{c_0 + i\tau _2\epsilon ^{\mu -\gamma }k + \epsilon ^{3\mu -\gamma }k^3N_2(\epsilon ^{\mu }k)}. \end{aligned}$$

Since $\gamma < \mu$ we find

$$\begin{aligned} \lim _{\epsilon \rightarrow 0^+} \frac{1+\epsilon ^{\mu }kN_1(\epsilon ^{\mu }k)}{c_0 + i\tau _2\epsilon ^{\mu -\gamma }k + \epsilon ^{3\mu -\gamma }k^3N_2(\epsilon ^{\mu }k)} = \frac{1}{c_0}. \end{aligned}$$

We would then want $2-3\gamma -2\mu = 0$ to prevent a nontrivial limit.

Choosing $\gamma$ and $\mu$ appropriately, we conclude that at $\epsilon = 0$ the equation for $\psi _1$ from (3.14) formally reduces to

$$\begin{aligned} \psi _1 = \frac{1}{c_0}\breve{\mathsf {R}}_1^0(\psi _1)\psi _1. \end{aligned}$$

Numerically we expect $\psi _1(X) > 0$ for all *X* when $\epsilon = 0$, and so, using the definition of $\breve{\mathsf {R}}_1^0$ from (3.8), we have

$$\begin{aligned} c_0 = \breve{\mathsf {R}}_1^0(\psi _1)(X) = \frac{\alpha \kappa \tau _1}{c_0^2}\int _X^{\infty } \left( \int _V^{\infty } \psi _1(W)\ dW\right) \psi _1(V) \ dV. \end{aligned}$$

Differentiating, we find

$$\begin{aligned} \left( \int _X^{\infty }\psi _1(W) \ dW\right) \psi _1(X) = 0. \end{aligned}$$

But since $\psi _1(W) > 0$ for all *W*, we cancel the integral factor to find $\psi _1(X) = 0$, a contradiction to our numerical predictions.

#### The formal leading order equation for $\psi _1$

At $\epsilon = 0$ the equation for $\psi _1$ in (3.14) becomes (again, formally)

$$\begin{aligned} \psi _1 = \mathcal {M}^{(0)}\big (\breve{\mathsf {R}}^0(\psi _1)\psi _1\big ) = (c_0+\tau _2\partial _X)^{-1}\big (\breve{\mathsf {R}}^0(\psi _1)\psi _1\big ). \end{aligned}$$

This is equivalent to

3.24 $$\begin{aligned} c_0\psi _1+\tau _2\psi _1' = \breve{\mathsf {R}}^0(\psi _1)\psi _1. \end{aligned}$$

We will rewrite this equation so that each term is a perfect derivative.

The definition of $\breve{\mathsf {R}}_1^{\epsilon }$ in (3.8), valid for all $\epsilon$, gives

3.25 $$\begin{aligned} \breve{\mathsf {R}}^0(\psi _1)(X) = \frac{\alpha \kappa \tau _1}{c_0^2}\int _X^{\infty } \left( \int _V^{\infty } \psi _1(W) \ dW\right) \psi _1(V) \ dV. \end{aligned}$$

Write

$$\begin{aligned} \Psi _1(X) := \int _X^{\infty } \psi _1(W) \ dW, \end{aligned}$$

so that $\Psi _1' = -\psi _1$. The double integral from (3.25) is

$$\begin{aligned} \int _X^{\infty } \left( \int _V^{\infty } \psi _1(W) \ dW\right) \psi _1(V) \ dV= & {} -\int _X^{\infty } \Psi _1(V)\Psi _1'(V) \ dV\\= & {} -\int _X^{\infty } \partial _V\left[ \frac{\Psi _1^2(V)}{2}\right] \ dV\\= & {} \frac{\Psi _1(X)^2}{2}. \end{aligned}$$

Here we are using the requirement that $\psi _1 \in H_q^1$, which implies $\Psi _1(X) \rightarrow 0$ as $X \rightarrow \infty$. Thus

$$\begin{aligned} \breve{\mathsf {R}}^0(\psi _1)\psi _1 = -\left( \frac{\alpha \kappa \tau _1}{2c_0^2}\right) \Psi _1^2\Psi _1' = -\left( \frac{\alpha \kappa \tau _1}{6c_0^2}\right) \partial _X[\Psi _1^3] . \end{aligned}$$

Then (3.24) is equivalent to

$$\begin{aligned} \tau _2\Psi _1'' + c_0\Psi _1' - \left( \frac{\alpha \kappa \tau _1}{6c_0^2}\right) \partial _X[\Psi _1^3] = 0. \end{aligned}$$

We integrate both sides from 0 to $\infty$ and use the aforementioned fact that $\Psi _1$ and its derivatives are required to vanish at $\infty$ to find

3.26 $$\begin{aligned} \tau _2\Psi _1' + c_0\Psi _1 - \left( \frac{\alpha \kappa \tau _1}{6c_0^2}\right) \Psi _1^3 = 0. \end{aligned}$$

This is a Bernoulli equation, and it has the solution

3.27 $$\begin{aligned} \Psi _1(X) = \Sigma (X) := \left( \frac{6c_0^3}{\alpha \kappa \tau _1+6c_0^2\exp \big (2c_0X/\tau _2+\theta \big )}\right) ^{1/2}. \end{aligned}$$

Here $\theta \in \mathbb {R}$ is an arbitrary phase shift. It follows that putting

3.28 $$\begin{aligned} \psi _1(X) = \sigma (X) := -\Sigma '(X) = \frac{(6c_0^3)^{3/2}\exp \big (2c_0X/\tau _2+\theta \big )}{\tau _2\big [\alpha \kappa \tau _1+6c_0^2\exp \big (2c_0X/\tau _2+\theta \big )\big ]^{3/2}} \end{aligned}$$

solves (3.24).

##### Remark 3.3

We view the free parameter $\theta$ in (3.28) as an artifact of the translation invariance of our original problem (1.1). Friesecke and Pego (1999) do not incorporate a phase shift like $\theta$ into their leading order ${{\,\mathrm{sech}\,}}^2$-type KdV solution, since their broader existence result relies on working in spaces of even functions, and phase shifts destroy evenness. We will not need such symmetry in our subsequent arguments (nor could we achieve it, since no translation of $\sigma$ is even or odd), and so we will leave $\theta$ as an arbitrary free parameter and not specify its value. Instead, we will restrain the extra degree of freedom of translation invariance by imposing a certain integral condition, which we make precise in (5.2).

#### The formal leading order equation for $\psi _2$ and the selection of the exponent $\beta$

From our choice of $\gamma =\mu =2/5$ and the inequalities in (3.15), we need, at the very least,

$$\begin{aligned} \frac{1}{10} \le \beta \le \frac{1}{5}. \end{aligned}$$

If the strict inequality $\beta < 1/5$ holds, then at $\epsilon = 0$ the equation for $\psi _2$ in (3.14) reduces to the trivial result $\psi _2 = 0$. This is not at all what we expect numerically from Fig. 2; rather, we anticipate that $\psi _2$ will asymptote to some nonzero constant at $\infty$.

However, if we instead take $\beta$ so that

$$\begin{aligned} 0 = 1-(\gamma +\mu +\beta ) = \frac{1}{5}-\beta , \end{aligned}$$

which is to say,

$$\begin{aligned} \beta = \frac{1}{5}, \end{aligned}$$

then the equation for $\psi _2$ in (3.14) at $\epsilon = 0$ becomes

$$\begin{aligned} \psi _2 = \breve{\mathsf {P}}_1^0(\psi _1). \end{aligned}$$

Putting

3.29 $$\begin{aligned} \psi _2(X) = \zeta (X) := \breve{\mathsf {P}}_1^0(\sigma )(X) = \frac{\alpha }{c_0}\int _X^{\infty } \sigma (V) \ dV\end{aligned}$$

therefore solves the leading order equation for $\psi _2$. We really have

$$\begin{aligned} \zeta (X) = \frac{\alpha }{c_0}\Sigma (X) = \frac{\alpha }{c_0}\left( \frac{6c_0^3}{\alpha \kappa \tau _1+6c_0^2e^{2c_0X/\tau _2+\theta }}\right) ^{1/2}, \end{aligned}$$

where $\Sigma$ was defined in (3.27).

### The final long wave system

With the choices of exponents $\gamma =\mu =2/5$ and $\beta = 1/5$, it becomes convenient to introduce the new small parameter

3.30 $$\begin{aligned} \nu := \epsilon ^{2/5} \end{aligned}$$

into the problem (3.14) and then recast that problem more cleanly in terms of $\nu$. First, the long wave Ansatz (3.1) becomes

3.31 $$\begin{aligned} \phi _1(x) = \nu ^{5/2}\psi _1(\nu {x}), \qquad \phi _2(x) = \nu ^{1/2}\psi _2(\nu {x}), \qquad \text { and }\qquad c = \nu {c}_0. \end{aligned}$$

Proceeding very much as in Sect. 3.1, we then define

3.32 $$\begin{aligned} \mathcal {Q}_1^{\nu }(X,Y ):= & {} \frac{X^2Y}{k_a(k_a+\nu ^{5/2}{X})} \qquad \text { and }\qquad \nonumber \\ \quad \mathcal {Q}_2^{\nu }(X,Y):= & {} \kappa \frac{k_rY+k_m\nu ^2X+\nu ^{5/2}{XY}}{(k_r+\nu ^{5/2}{X})(k_m+\nu ^{1/2}Y)}XY \end{aligned}$$

for *X*, $Y \in \mathbb {R}$, while for $f \in L^1$ and $g \in L^{\infty }$, we put

3.33 $$\begin{aligned} \mathcal {P}_1^{\nu }(f)(X) := \frac{\alpha }{c_0}\int _X^{\infty } \mathcal {E}(\nu ^{1/2}S^{\nu }f)(V,X)f(V) \ dV, \end{aligned}$$

where $\mathcal {E}$ was defined in (3.3), and

3.34 $$\begin{aligned} \mathcal {P}_2^{\nu }(f,g)(X):= & {} \frac{1}{c_0}\int _X^{\infty } \mathcal {E}(\nu ^{1/2}S^{\nu }f)(V,X)\mathcal {Q}_2^{\nu }(f(V+\nu ),g(V)) \ dV, \end{aligned}$$

3.35 $$\begin{aligned} \mathcal {R}_1^{\nu }(f)(X):= & {} \frac{\kappa \tau _1}{c_0}\int _X^{\infty } \mathcal {P}_1^{\nu }(f)(V)f(V + \nu ) \ dV, \end{aligned}$$

3.36 $$\begin{aligned} \mathcal {R}_2^{\nu }(f,g)(X):= & {} \frac{1}{c_0}\int _X^{\infty } \big (\nu ^{1/2}\kappa {f}(V+\nu )\mathcal {P}_2^{\nu }(f,g)(V)\nonumber \\&-\mathcal {Q}_2^{\nu }(f(V+\nu ),g(V))\big ) \ dV, \end{aligned}$$

3.37 $$\begin{aligned} \mathcal {R}^{\nu }(f,g)(X):= & {} \mathcal {R}_1^{\nu }(f)(X) + \nu ^{1/2}\mathcal {R}_2^{\nu }(f,g)(X), \end{aligned}$$

and

3.38 $$\begin{aligned} \mathcal {N}^{\nu }(f,g)(X) := \tau _1\mathcal {R}_2^{\nu }(f,g)(X)f(X) - \nu ^{3/2}\tau _1\mathcal {Q}_1^{\nu }(f(X),\mathcal {R}^{\nu }(f,g)(X)). \nonumber \\ \end{aligned}$$

#### Remark 3.4

The operators $\mathcal {P}_1^{\nu }$ and $\mathcal {R}_1^{\nu }$ map $L^1$ into $L^{\infty }$, while $\mathcal {P}_2^{\nu }$ and $\mathcal {R}_2^{\nu }$ map $L^1 \times L^{\infty }$ into $L^{\infty }$, and $\mathcal {N}^{\nu }$ maps $L^1 \times L^{\infty }$ into $L^1$. More precisely, we could replace $L^1$ with $H_q^1$ and $L^{\infty }$ with $W^{1,\infty }$ and the preceding statement would still be true; see the estimates in Appendix B.1.

The operator $\mathcal {R}_1^0$ has the especially simple form

3.39 $$\begin{aligned} \mathcal {R}_1^0(f)(X) = \left( \frac{\alpha \kappa \tau _1}{6c_0^2}\right) \int _X^{\infty } \left( \int _V^{\infty } f(W) \ dW\right) f(V) \ dV\end{aligned}$$

and therefore is differentiable from $L^1$ to $L^{\infty }$.

Last, for $\nu > 0$, let $\mathcal {M}^{(\nu )}$ be the Fourier multiplier with symbol

3.40 $$\begin{aligned} \widetilde{\mathcal {M}}^{(\nu )}(z) := \nu \frac{1-e^{-i\nu {z}}}{ic_0\nu ^2z+2\tau _2(\cos (\nu {z})-1)}. \end{aligned}$$

When $\nu = 0$ we have already defined $\mathcal {M}^{(0)}$ as the Fourier multiplier whose symbol $\widetilde{\mathcal {M}}^{(0)}$ is given in (3.21). We will show momentarily in Sect. 4 how $\mathcal {M}^{(0)}$ is a good approximation of $\mathcal {M}^{(\nu )}$ in the operator norm topology, which we formally anticipated in the limit (3.23).

We now summarize the work of this section and the preceding one. In Sect. 2 we reformulated our original problem (1.1) into the more concise structure (2.14) and then made a travelling wave ansatz on this latter system. That led to the travelling wave problem (2.28). In this section, we introduced the long wave Ansatz (3.1) into this travelling wave problem and studied several formal expansions and limits to deduce the ‘correct’ choice of exponents and scalings. With the operators defined above, we can summarize our long wave problem in the following form.

#### Proposition 3.5

Suppose

3.41 $$\begin{aligned} {\left\{ \begin{array}{ll} A_j(t) = \nu ^{5/2}\psi _1(\nu (j-\nu {c}_0t)), \\ P_j(t) = \nu ^{1/2}\psi _2(\nu (j-\nu {c}_0t)) \end{array}\right. } \end{aligned}$$

for some $\psi _1 \in H_q^1$ and $\psi _2 \in L^{\infty }$, where $c_0$, $\nu > 0$ and $q \in (0,c_0/\tau _2)$. Then $A_j$ and $P_j$ satisfy (2.14) if and only if $\psi _1$ and $\psi _2$ satisfy

3.42 $$\begin{aligned} {\left\{ \begin{array}{ll} \psi _1 = \mathcal {M}^{(\nu )}\big (\mathcal {R}_1^{\nu }(\psi _1)\psi _1 + \nu ^{1/2}\mathcal {N}^{\nu }(\psi _1,\psi _2)\big ), \\ \psi _2 = \mathcal {P}_1^{\nu }(\psi _1) + \nu \mathcal {P}_2^{\nu }(\psi _1,\psi _2). \end{array}\right. } \end{aligned}$$

Moreover, taking

$$\begin{aligned} \psi _1 = \sigma \qquad \text { and }\qquad \psi _2 = \zeta = \mathcal {P}_1^0(\sigma ), \end{aligned}$$

where $\sigma$ is defined in (3.28) and $\zeta$ is given explicitly in (3.29), solves (3.42) when $\nu = 0$.

We will analyze the system (3.42) with a quantitative contraction mapping argument that tracks its dependence on $\nu$. Specifically, we will look for solutions $\psi _1$ and $\psi _2$ to (3.42) that are close to $\sigma$ and $\zeta$, respectively, when $\nu$ is close to 0. We provide the details of this argument in Sect. 6.

Before doing so, we need to understand the behavior of two key operators, on whose good behavior the successful contraction argument will hinge. Our first task, as we mentioned above, is to study how $\mathcal {M}^{(0)}$ approximates $\mathcal {M}^{(\nu )}$, and we do this in Sect. 4. Next, since we are looking for solutions $(\psi _1,\psi _2)$ to (3.42) that are close to $(\sigma ,\zeta )$, it is natural to study the linearization of (3.42) at $(\sigma ,\zeta )$ for $\nu =0$. It turns out that we will only need the linearization of the first equation, which is the operator

3.43 $$\begin{aligned} \mathcal {T}\psi := \psi -\mathcal {M}^{(0)}\big [\mathcal {R}_1^0(\sigma )\psi +(D\mathcal {R}_1^0(\sigma )\psi )\sigma \big ]. \end{aligned}$$

We will show in Sect. 5 that $\mathcal {T}$ is surjective from $H_q^1$ to $H_q^1$ with a one-dimensional kernel. Restricting $\mathcal {T}$ off this kernel will yield an extremely useful bijectivity result.

## Analysis of the Fourier multiplier $\mathcal {M}^{(\nu )}$

We show that the Fourier multiplier $\mathcal {M}^{(\nu )}$, whose symbol was defined in (3.40), converges to the multiplier $\mathcal {M}^{(0)}$, whose symbol was defined in (3.21). More precisely, we prove the following estimate.

### Proposition 4.1

Fix $q \in (0,c_0/\tau _2)$. There exist $\nu _{\mathcal {M}}$, $C_{\mathcal {M}} > 0$ such that if $0< \nu < \nu _{\mathcal {M}}$, then

$$\begin{aligned} \Vert \mathcal {M}^{(\nu )}-\mathcal {M}^{(0)}\Vert _{\mathbf {B}(H_q^1)} \le C_{\mathcal {M}}\nu ^{1/3}. \end{aligned}$$

The proof of this estimate depends on the following lemma, whose proof we give in the remainder of this section.

### Lemma 4.2

Let $q \in (0,c_0/\tau _2)$. There exist $C_{\mathcal {M}}$, $\nu _{\mathcal {M}} > 0$ such that if $0< \nu < \nu _{\mathcal {M}}$, then the map $\widetilde{\mathcal {M}}^{(\nu )}$ defined in (3.40) is analytic on the strip $\overline{\mathcal {U}}_q := \!\left\{ z \in \mathbb {C} \ | \ |{{\,\mathrm{Im}\,}}(z)| \le q\right\}$ and satisfies

4.1 $$\begin{aligned} \sup _{k \in \mathbb {R}} \big |\widetilde{\mathcal {M}}^{(\nu )}(k \pm iq) - \widetilde{\mathcal {M}}^{(0)}(k\pm {iq})\big | < C_{\mathcal {M}}\nu ^{1/3}, \end{aligned}$$

where $\widetilde{\mathcal {M}}^{(0)}$ was defined in (3.21).

This lemma allows us to invoke Beale’s result in Lemma A.2 (with $r=1$ and $s=0$) to prove Proposition 4.1. Beale’s result depends very much on our working in exponentially weighted Sobolev spaces, and this is another of our reasons for preferring these spaces to algebraically weighted ones.

We will estimate the difference $\big |\widetilde{\mathcal {M}}^{(\nu )}(z)-\widetilde{\mathcal {M}}^{(0)}(z)\big |$ over two regimes, one in which $z = k\pm {iq}$ is ‘close’ to 0, and the other in which *z* is ‘far from’ 0. Part of these estimates will involve bounding the denominator of $\widetilde{\mathcal {M}}^{(\nu )}$ away from zero; this will ensure the analyticity of $\widetilde{\mathcal {M}}^{(\nu )}$, since it is the quotient of two analytic functions.

To quantify these regimes, we introduce two positive constants *p* and *m*; we say that *z* is ‘close’ to 0 if $|z| \le \nu ^{-p}$ and ‘far from’ 0 if $|z| > \nu ^{-p}$. The constant *m* will later control how close the real part of $\nu {z}$ is to an integer multiple of $2\pi$, a bound that will be very useful in certain estimates to come. All constants *C* in the work below are allowed to depend on *m*, *p*, and *q*, but they are always independent of $\nu$ and *z*.

Our estimates will depend on the parameters *p* and *m*; once we have all the estimates together, we will choose useful values for *p* and *m*. We feel that this approach allows the otherwise nonobvious final values for *p* and *m* to emerge very naturally. This strategy of splitting the estimates over regions close to and far from 0 is modeled on the proofs of Faver and Wright (2018, Lem. A.13) and Stefanov and Wright (2020, Lem. 3) and the strategy in Johnson and Wright (2020, App. A.3). Friesecke and Pego Friesecke and Pego (1999, Sec. 3) give a rather different proof of symbol convergence that relies on more knowledge of the poles of $\widetilde{\mathcal {M}}^{(\nu )}$ than we care to discover.

### Estimates for *z* ‘close to’ 0

In this regime we fix $|z| \le \nu ^{-p}$. We recall the Taylor expansions

$$\begin{aligned} 1-e^{-iz} = iz+iz^2N_1(z) \qquad \text { and }\qquad \cos (z)-1 = -\frac{z^2}{2}+ \frac{iz^4N_2(z)}{2\tau _2} \end{aligned}$$

from (3.17), as well as the estimate

$$\begin{aligned} C_q := \sup _{x \in \mathbb {R}} |N_1(x\pm {iq})| + |N_2(x\pm {iq})| < \infty . \end{aligned}$$

Now we can write

$$\begin{aligned} \widetilde{\mathcal {M}}^{(\nu )}(z) = \frac{1+\nu {z}N_1(\nu {z})}{c_0+\tau _2iz+ \nu ^2z^3N_2(\nu {z})}. \end{aligned}$$

With this expression we find the following equality

$$\begin{aligned} \widetilde{\mathcal {M}}^{(\nu )}(z) - \widetilde{\mathcal {M}}^0(z) = I_{\nu }(z) + I\!I_{\nu }(z), \end{aligned}$$

where

4.2 $$\begin{aligned} I_{\nu }(z) := \frac{c_0\nu {z}N_1(\nu {z}) - \nu ^2z^3N_2(\nu {z})}{(c_0 + \tau _2 i z + \nu ^2 z^3 N_2(\nu z))(c_0 +i \tau _2 z)} \end{aligned}$$

and

4.3 $$\begin{aligned} I\!I_{\nu }(z) := \frac{i\tau _2\nu {z}^2N_1(\nu {z})}{(c_0 + \tau _2 i z + \nu ^2 z^3 N_2(\nu z))(c_0 +i \tau _2 z)}. \end{aligned}$$

We work on the denominators. We use the reverse triangle inequality to find

$$\begin{aligned}&\big |\big (c_0+\tau _2iz- 2\tau _2 i \nu ^2z^3N_2(\nu {z})\big )(c_0+\tau _2iz)\big | \ge \big |c_0-\tau _2q-2 \tau _2\nu ^2|z|^3|N_2(\nu {z})|\big |\\&\quad |c_0-\tau _2q|. \end{aligned}$$

As $q \in (0, c_0/\tau _2)$, we have $|c_0-\tau _2 q| > 0$. Also, since $|z| \le \nu ^{-p}$, we have $\nu ^2 |z|^3 \le \nu ^{2-3p}$. If we take

4.4 $$\begin{aligned} 0< p < \frac{2}{3} \end{aligned}$$

and assume $\nu \in (0,\nu _1)$, where

4.5 $$\begin{aligned} \nu _1 := \min \left\{ 1,\left( \frac{|c_0-\tau _2q|}{4C_q\tau _2}\right) ^{1/(2-3p)}\right\} , \end{aligned}$$

then

4.6 $$\begin{aligned} \big |c_0-\tau _2q-\nu ^2|z|^3|N_2(\nu {z})|\big | \ge \frac{|c_0-\tau _2q|}{2}. \end{aligned}$$

In particular,

4.7 $$\begin{aligned} \big |c_0-\tau _2q-2 \tau _2\nu ^2|z|^3|N_2(\nu {z})|\big ||c_0-\tau _2q| \ge \frac{|c_0-\tau _2q|^2}{2}, \end{aligned}$$

and this inequality guarantees that $\widetilde{\mathcal {M}}^{(\nu )}$ is defined (and analytic) for $|z| \le \nu ^{-p}$ and $|{{\,\mathrm{Im}\,}}(z)| < q$. Then we use (4.7) to estimate $I_{\nu }(z)$ from (4.2) as

$$\begin{aligned} |I_{\nu }(z)| \le C\nu ^{1-p} + C\nu ^{2-3p}. \end{aligned}$$

Next, we use (4.6) to estimate $I\!I_{\nu }(z)$ from (4.3) as

$$\begin{aligned} |I\!I_{\nu }(z)| \le C\nu ^{1-p}\frac{|z|}{|c_0+i\tau _2z|}. \end{aligned}$$

Setting $z=x \pm iq$ we note

$$\begin{aligned} \frac{|z|^2}{|c_0+i\tau _2z|^2} = \frac{x^2+q^2}{(c_0\pm \tau _2q)^2 + \tau _2^2x^2} \le \frac{x^2+q^2}{(c_0-\tau _2q)^2 + \tau _2^2x^2}. \end{aligned}$$

We know

$$\begin{aligned} D:= \sup _{x \in \mathbb {R}} \frac{x^2+q^2}{(c_0-\tau _2q)^2 + c_0^2x^2} < \infty , \end{aligned}$$

and thus

$$\begin{aligned} |I\!I_{\nu }(z)| \le C\nu ^{1-p}. \end{aligned}$$

We conclude

4.8 $$\begin{aligned} \big |\widetilde{\mathcal {M}}^{(\nu )}(z) - \widetilde{\mathcal {M}}^{(0)}(z)\big | \le |I_{\nu }(z)| + |I\!I_{\nu }(z)| \le C\big (\nu ^{1-p} + \nu ^{2-3p}\big ). \end{aligned}$$

As we required $p \in (0,2/3)$, the final estimate contains only positive powers of $\nu$. Since we will always consider $0< \nu < \nu _1$ in the future, the definition of $\nu _1$ in (4.5) ensures $0< \nu < 1$ in the following regimes.

### Estimates for *z* ‘far from’ 0

In this regime we assume $|z| > \nu ^p$. Take

4.9 $$\begin{aligned} \nu _2 < \min \left\{ \nu _1,\left( \frac{\tau _2}{2c_0}\right) ^{1/p}\right\} , \end{aligned}$$

with $\nu _1$ defined in (4.5), so that if $0< \nu < \nu _2$, then $|z| > c_0/\tau _2$. With the reverse triangle inequality we find

4.10 $$\begin{aligned} |\widetilde{\mathcal {M}}^{(0)}(z)| \le \frac{1}{\big ||c_0| - |\tau _2 z|\big |}< \frac{1}{\tau _2 \nu ^{-p}-c_0} < \frac{2}{\tau _2} \nu ^{p}. \end{aligned}$$

Consequently, it suffices in this regime to show that $\widetilde{\mathcal {M}}^{(\nu )}$ is bounded by a multiple of some power of $\nu$. It will be convenient now to rewrite $\widetilde{\mathcal {M}}^{(\nu )}$ as

$$\begin{aligned} \widetilde{\mathcal {M}}^{(\nu )}(z) = \frac{\nu \widetilde{\mathcal {M}}_1^{(\nu )}(z)}{\widetilde{\mathcal {M}}_2^{(\nu )}(z)}, \end{aligned}$$

where

4.11 $$\begin{aligned} \widetilde{\mathcal {M}}_1^{(\nu )}(z) := 1-e^{-i\nu {z}} \qquad \text { and }\qquad \widetilde{\mathcal {M}}_2^{(\nu )}(z) := ic_0\nu ^2z + 2\tau _2(\cos (\nu {z})-1). \nonumber \\ \end{aligned}$$

The analyticity of $\widetilde{\mathcal {M}}^{(\nu )}$ for $|z| > \nu ^p$ will follow if we bound $\widetilde{\mathcal {M}}_2^{(\nu )}$ away from zero here.

The presence of the factor $\cos (\nu {z})-1$ in the denominator of $\widetilde{\mathcal {M}}^{(\nu )}$ suggests that the behavior of this function may be different when ${{\,\mathrm{Re}\,}}(\nu {z})$ is ‘close’ to an integer multiple of $2\pi$ and when it is not. For this reason, we expand $z=x \pm iq$ and let $n \in \mathbb {Z}$ be the unique integer such that $|\nu {x}-2\pi {n}| \le \pi$. We consider three cases on the behavior of $\nu {x}$ and *n*.

#### Estimates for ${{\,\mathrm{Re}\,}}(\nu {z})$ ‘close to’ a nonzero integer multiple of $2\pi$

In this regime we assume $|\nu {x}-2\pi {n}| \le \nu ^m$ with $n \ne 0$.

We first rewrite the numerator as

$$\begin{aligned} \widetilde{\mathcal {M}}_1^{(\nu )}(z) = 1-e^{-i(\nu {x}-2\pi {n)}}+e^{-i\nu {x}}(1-e^{\pm \nu {q}}). \end{aligned}$$

Since the map $y \mapsto e^{-iy}$ is uniformly Lipschitz on $\mathbb {R}$ we have

$$\begin{aligned} |1-e^{-i(\nu {x}-2\pi {n})}| \le |\nu {x}-2\pi {n}| \le \nu ^m. \end{aligned}$$

Since the map $y \mapsto e^{-y}$ is locally Lipschitz on $\mathbb {R}$ we have, if we take $0< \nu < \nu _3$ with

4.12 $$\begin{aligned} \nu _3 := \min \left\{ \nu _2,\frac{1}{q}\right\} \end{aligned}$$

and $\nu _2$ defined in (4.9), the estimate

$$\begin{aligned} |1-e^{\pm \nu {q}}| \le \nu {q}. \end{aligned}$$

Then

4.13 $$\begin{aligned} \big |\widetilde{\mathcal {M}}_1^{(\nu )}(z)\big | \le \nu ^m + \nu {q} \le C(\nu ^m+\nu ). \end{aligned}$$

We remark that we did not need $n \ne 0$ here, although we will momentarily.

We now turn to the denominator, $\mathcal {M}_2^{(\nu )}(z)$. Using the identity

4.14 $$\begin{aligned} \cos (a+bi) = \cos (a)\cosh (b)-i\sin (a)\sinh (b) \end{aligned}$$

for $a,b \in \mathbb {R}$ we find

$$\begin{aligned} {{\,\mathrm{Im}\,}}\big (\widetilde{\mathcal {M}}_1^{(\nu )}(z)\big ) = c_0 \nu ^2 x - 2 \tau _2 \sin (\nu x) \sinh (\nu q). \end{aligned}$$

We estimate

$$\begin{aligned} \big |{{\,\mathrm{Im}\,}}\big (\widetilde{\mathcal {M}}_1^{(\nu )}(z)\big ) \big | \ge C\big (\nu |n| - \nu |\nu {x}-2n\pi | - |\sin (\nu {x}-2n\pi )||\sinh (\nu {q})|\big ) \end{aligned}$$

We control the three terms on the right as follows. First, $|n| \ge 1$. Next, we are in the regime $|\nu {x}-2n\pi | \le \nu ^m$. Finally, we have

$$\begin{aligned} |\sin (\nu {x}-2n\pi )| \le |\nu {x}-2n\pi | \le \nu ^m \qquad \text { and }\qquad |\sinh (\nu {q})| \le 2|\nu {q}|, \end{aligned}$$

since $|\nu {q}| \le 1$. We thus find

$$\begin{aligned} \big |{{\,\mathrm{Im}\,}}\big (\widetilde{\mathcal {M}}_2^{(\nu )}(z)\big )\big | \ge C\big (\nu -\nu ^{m+1}). \end{aligned}$$

Now that we have the numerator and the denominator bounded, we can conclude

4.15 $$\begin{aligned} \big |\widetilde{\mathcal {M}}^{(\nu )}(z)\big | \le C\nu \frac{\nu ^m+\nu }{\nu -\nu ^{m+1}} = C\frac{\nu ^m+\nu }{1-\nu ^m} \le C\nu ^m. \end{aligned}$$

Here we need to assume

4.16 $$\begin{aligned} 0< m < 1. \end{aligned}$$

#### Estimates for ${{\,\mathrm{Re}\,}}(\nu {z})$ ‘close to’ 0

In this regime we assume $|\nu {x}| \le \nu ^m$; in particular, we are taking $n = 0$. We will need the following bound on the cosine, which is a consequence of an elementary argument with Taylor’s theorem.

##### Lemma 4.3

Let $Q \ge 0$. There exist $C_{1,Q}$, $C_{2,Q} > 0$ such that if $Z \in \mathbb {C}$ with $|Z| \le C_{1,Q}$ and $|{{\,\mathrm{Im}\,}}(Z)| \le Q$, then

$$\begin{aligned} |\cos (Z)-1| \ge C_{2,Q}|Z|^2. \end{aligned}$$

In particular, if $Q = 0$, then $C_{1,0} > \pi$.

We use the reverse triangle inequality on $\widetilde{\mathcal {M}}_2^{(\nu )}$ from (4.11) to find

4.17 $$\begin{aligned} \big |\widetilde{\mathcal {M}}_2^{(\nu )}(z)\big | \ge 2\tau _2|\cos (\nu {z})-1| - c_0\nu ^2q - c_0\nu ^2|x|. \end{aligned}$$

Take $$0< \nu < \nu _{\mathcal {M}}$$, where

4.18 $$\begin{aligned} \nu _{\mathcal {M}} < \min \left\{ \nu _3,\left( \frac{1}{q}\right) ^{1/(1-m)}, \left( \frac{C_{1,q}}{2}\right) ^{1/m}\right\} , \end{aligned}$$

with $\nu _3$ defined in (4.12), to find

$$\begin{aligned} |\nu z | \le \nu ^{m} + \nu {z} \le 2 \nu ^{m} < C_{1,q}. \end{aligned}$$

Lemma 4.3 then guarantees

$$\begin{aligned} |\cos (\nu {z})-1| \ge C|\nu {z}|^2 \ge C\nu ^{2-2p}. \end{aligned}$$

Finally, since $|x| \le \nu ^{m-1}$ in this regime we use the bound (4.17) to conclude

$$\begin{aligned} \big |\widetilde{\mathcal {M}}_2^{(\nu )}(z)\big | \ge C\big (\nu ^{2-2p} - \nu ^2 - \nu ^{m+1}\big ). \end{aligned}$$

We remark that the derivation of the estimate (4.13) only assumed $|\nu {x}-2\pi {n}| \le \nu ^m$ and did not rely on having $n \ne 0$. So it is still valid here, and we conclude

4.19 $$\begin{aligned} \big |\widetilde{\mathcal {M}}^{(\nu )}(z)\big |\le & {} C\nu \frac{\nu ^m+\nu }{\nu ^{2-2p}-\nu ^2-\nu ^{m+1}} = C\frac{\nu ^{m+2p-1}+\nu ^{2p}}{1-\nu ^{2p}-\nu ^{m+2p-1}}\nonumber \\\le & {} C\big (\nu ^{m+2p-1}+\nu ^{2p}\big ). \end{aligned}$$

Here we are assuming

4.20 $$\begin{aligned} 0 \le 1-2p < \min \{1,m\}. \end{aligned}$$

#### Estimates for ${{\,\mathrm{Re}\,}}(\nu {z})$ ‘far from’ a nonzero integer multiple of $2\pi$

In this regime we assume $|\nu {x}-2\pi {n}| > \nu ^m$. We do not perform separate work on $n = 0$ and $n \ne 0$.

Via (4.14) we find

$$\begin{aligned} {{\,\mathrm{Re}\,}}\big (\mathcal {M}_2^{(\nu )}(z)\big ) = -c_0\nu ^2q + 2\tau _2(\cos (\nu {x}-2n\pi )-1) + 2\tau _2\cos (\nu {x})(\cosh (\nu {q})-1). \end{aligned}$$

We estimate

$$\begin{aligned} \big |{{\,\mathrm{Re}\,}}\big (\mathcal {M}_2^{(\nu )}(z)\big )\big | \ge C\big (|\cos (\nu {x}-2n\pi )-1|-|\cos (\nu {x})||\cosh (\nu {q})-1|-\nu ^2\big ). \end{aligned}$$

Now we use Lemma 4.3 with $Q = 0$ to bound

$$\begin{aligned} |\cos (\nu {x}-2n\pi )-1| \ge C|\nu {z}-2n\pi |^2 \ge C\nu ^{2m}. \end{aligned}$$

Also, a routine Lipschitz estimate on the hyperbolic cosine gives

$$\begin{aligned} |\cos (\nu {x})||\cosh (\nu {q})-1| \le C\nu ^2 \end{aligned}$$

since $|\nu {q}| \le 1$. We thus find

$$\begin{aligned} \big |{{\,\mathrm{Re}\,}}\big (\mathcal {M}_2^{(\nu )}(z)\big )\big | \ge C(\nu ^{2m}-\nu ^2). \end{aligned}$$

As we are assuming $0< m < 1$ from (4.16), this is a positive lower bound.

Finally, we bound the numerator $\widetilde{\mathcal {M}}_1^{\nu }(z)$ crudely as $\big |\widetilde{\mathcal {M}}_1^{(\nu )}(z)\big | \le C$ for all $z \in \mathbb {C}$ with $|{{\,\mathrm{Im}\,}}(z)| = q$. This follows from the boundedness of $Z \mapsto e^{iZ}$ on strips. We conclude

4.21 $$\begin{aligned} \big |\widetilde{\mathcal {M}}^{(\nu )}(z)\big | < C\frac{\nu }{\nu ^{2m}-\nu ^2} \le C\nu ^{1-2m}. \end{aligned}$$

This is a positive bound if we now require

4.22 $$\begin{aligned} 0< m < \frac{1}{2}. \end{aligned}$$

### Overall estimates

Suppose $0< \nu < \nu _{\mathcal {M}}$, where $\nu _{\mathcal {M}}$ was specified in (4.18). We conclude from (4.8) that

4.23 $$\begin{aligned} \sup _{\begin{array}{c} |{{\,\mathrm{Im}\,}}(z)| = q \\ |z| \le \nu ^{-p} \end{array}} \big |\widetilde{\mathcal {M}}^{(\nu )}(z) - \widetilde{\mathcal {M}}^{(0)}(z)\big | \le C\big (\nu ^{1-p} + \nu ^{2-3p}\big ) \end{aligned}$$

and, by combining (4.10), (4.15), (4.19), and (4.21), that

4.24 $$\begin{aligned} \sup _{\begin{array}{c} |{{\,\mathrm{Im}\,}}(z)| = q \\ |z| > \nu ^{-p} \end{array}} \big |\widetilde{\mathcal {M}}^{(\nu )}(z) - \widetilde{\mathcal {M}}^{(0)}(z)\big | \le C\nu ^p + C\max \big \{\nu ^m, \nu ^{m+2p-1}+\nu ^{2p}, \nu ^{1-2m}\big \}. \nonumber \\ \end{aligned}$$

Additionally, we need, per (4.4), and (4.20), and (4.22), the exponents *p* and *m* to satisfy

4.25 $$\begin{aligned} 0< p< \frac{2}{3}, \qquad 0< m< \frac{1}{2}, \qquad \text { and }\qquad 0< 1-2p < \min \{1,m\}. \end{aligned}$$

There are many possible choices of *p* and *m* that will satisfy (4.25). Purely for convenience, we elect to take $m = 1/3$ and then $p = 1/2$. We combine (4.23) and (4.24) to conclude the estimate (4.1).

## Analysis of the linearization $\mathcal {T}$

The operator

5.1 $$\begin{aligned} \mathcal {T}\psi = \psi -\mathcal {M}^{(0)}\big [\mathcal {R}_1^0(\sigma )\psi +(D\mathcal {R}_1^0(\sigma )\psi )\sigma \big ] \end{aligned}$$

is the linearization of the first equation in our long wave-scaled travelling wave problem (3.42) at $(\psi _1,\psi _2) = (\sigma ,\zeta )$ and $\nu =0$. We recall that the symbol of the Fourier multiplier $\mathcal {M}^{(0)}$ was defined in (3.21) and the operator $\mathcal {R}_1^0$ was defined in (3.39).

We will work with $\mathcal {T}$ defined on the following subspace of $H_q^1$:

5.2 $$\begin{aligned} H_{q,0}^1 := \!\left\{ f \in H_q^1 \ | \ \int _0^{\infty } f(W) \ dW= 0\right\} . \end{aligned}$$

We norm $H_{q,0}^1$ with the $H_q^1$-norm. In Lemma 5.2 below we show that the kernel of $\mathcal {T}$ in $H_q^1$ is spanned by $\sigma '$. Restricting $\mathcal {T}$ to $H_{q,0}^1$ removes this kernel and guarantees injectivity. We will then prove that $\mathcal {T}$ is surjective onto $H_q^1$ and so conclude the following result.

### Proposition 5.1

For $q \in (0,c_0/\tau _2)$, the operator $\mathcal {T}:H_{q,0}^1 \rightarrow H_q^1$ is invertible with bounded inverse.

The linearization at the limiting localized solution appears as a key operator in numerous FPUT problems, including (Friesecke and Pego 1999; Faver and Wright 2018; Hoffman and Wright 2017), and the invertibility of this operator is a property essential to the development of the right fixed point formula for the given problem. Our treatment of the invertibility of $\mathcal {T}$ is rather different from the analogous inversions in those papers, as the problem $\mathcal {T}{f}=g$ is really a linearized Bernoulli equation in disguise, rather than the linearized KdV travelling wave profile equation. In particular, solving $\mathcal {T}{f} = g$ amounts to studying a first-order linear problem, which we can solve explicitly with an integrating factor. In doing so, we avoid the more abstract spectral theory that controls the second-order KdV linearizations (see, e.g., Friesecke and Pego (1999, Lem. 4.2)).

It will be convenient to abbreviate

5.3 $$\begin{aligned} q_* := \frac{c_0}{\tau _2}, \end{aligned}$$

and in the following we always assume $0< q < q_*$.

### The proof of Proposition 5.1

We will reformulate the equation $\mathcal {T}{f} = g$ in a very convenient manner, which we summarize in Lemma 5.2 below. This lemma will be the key to deducing the injectivity and surjectivity of $\mathcal {T}$.

First, for $h \in H_q^1$, we introduce the operator

5.4 $$\begin{aligned} (\mathcal {A}{f})(X) := \int _X^{\infty } h(W) \ dW, \end{aligned}$$

so that

5.5 $$\begin{aligned} h = -\partial _X[\mathcal {A}{h}]. \end{aligned}$$

Then $h \in H_{q,0}^1$ if and only if $h \in H_q^1$ and $(\mathcal {A}{f})(0) = 0$. We will show that the equation $\mathcal {T}{f} = g$ for *f*, $g \in H_q^1$ is equivalent to a statement about $\mathcal {A}{f}$ and $\mathcal {A}{g}$, which we give precisely in Lemma 5.2.

The following steps are quite similar to the derivation of the Bernoulli solution $\sigma$ in Sect. 3.2.2. From the definition of $\mathcal {T}$ in (5.1), we have $\mathcal {T}{f} = g$ if and only if

5.6 $$\begin{aligned} (c_0+\tau _2\partial _X)f - \mathcal {R}_1^0(\sigma )f + \big (D\mathcal {R}_1^0(\sigma )f\big )\sigma = g. \end{aligned}$$

From the definition of $\mathcal {R}_1^0$ in (3.39), we find

5.7 $$\begin{aligned} \big (D\mathcal {R}^0(\sigma )f\big )(X)= & {} \int _X^{\infty } \left( \int _W^{\infty } \sigma (V) \ dV\right) f(W) \ dW\nonumber \\&+ \int _X^{\infty } \left( \int _W^{\infty } f(V) \ dV\right) \sigma (W) \ dW. \end{aligned}$$

Since *g*, $\sigma \in H_q^1$, and since we seek $f \in H_q^1$, we abbreviate

5.8 $$\begin{aligned} F := \mathcal {A}{f}, \qquad G := \mathcal {A}{g}, \qquad \text { and }\qquad \Sigma := \mathcal {A}\sigma . \end{aligned}$$

Then (5.6) is equivalent to

5.9 $$\begin{aligned}&\tau _2F''(X) -c_0F'(X) - \left( \frac{\alpha \kappa \tau _1}{c_0^2}\right) F'(X)\int _X^{\infty } \Sigma (W)\Sigma '(W) \ dW\nonumber \\&\quad - \left( \frac{\alpha \kappa \tau _1}{c_0^2}\right) \Sigma '(X)\int _X^{\infty } \big (\Sigma (W)F'(W) + F(W)\Sigma '(W)\big ) \ dW\nonumber \\&\qquad =-c_0G'(X) -\tau _2G''(X). \end{aligned}$$

Although it may not be apparent at first glance, every term in this equation is a perfect derivative. First, since $\Sigma$ and *F* must vanish at $+\infty$, we have

$$\begin{aligned} \int _X^{\infty } \Sigma (W)\Sigma '(W) \ dW= -\frac{\Sigma (X)^2}{2} \end{aligned}$$

and

$$\begin{aligned} \int _X^{\infty } \big (\Sigma (W)F'(W) + F(W)\Sigma '(W)\big ) \ dW= -\Sigma (X)F(X). \end{aligned}$$

Hence (5.9) really is

5.10 $$\begin{aligned} \tau _2F''-c_0F' +\left( \frac{\alpha \kappa \tau _1}{c_0^2}\right) \left( \frac{F'\Sigma ^2}{2} + \Sigma '\Sigma {F}\right) = -c_0G' - \tau _2G'', \end{aligned}$$

where

$$\begin{aligned} \frac{F'\Sigma ^2}{2} + \Sigma '\Sigma {F} = \frac{1}{2}\partial _X[\Sigma ^2F]. \end{aligned}$$

So, we deduce that *F* and *G* must satisfy

5.11 $$\begin{aligned} - \tau _2F'' -c_0F' + \left( \frac{\alpha \kappa \tau _1}{c_0^2}\right) \partial _X\left[ \frac{\Sigma ^2F}{2}\right] = -c_0G' - \tau _2G''. \end{aligned}$$

Since both *F* and *G* must vanish at $+\infty$, we may integrate (5.11) to find

5.12 $$\begin{aligned} \underbrace{\tau _2F' + c_0F - \left( \frac{\alpha \kappa \tau _1}{c_0^2}\right) \frac{\Sigma ^2F}{2}}_{{\displaystyle {\mathcal {L}{F}}}} = c_0G + \tau _2G'. \end{aligned}$$

The operator $\mathcal {L}$ defined above is the linearization of the Bernoulli equation (3.26) at its solution $\Sigma$, and so

5.13 $$\begin{aligned} 0 = \mathcal {L}\Sigma ' = \mathcal {L}(-\sigma ). \end{aligned}$$

The operator $\mathcal {L}$, or, more precisely, $\tau _2^{-1}\mathcal {L}$, is also a first-order linear differential operator, and so we can solve (5.12) with an integrating factor. Namely, let ${{\,\mathrm{P}\,}}$ satisfy

5.14 $$\begin{aligned} {{\,\mathrm{P}\,}}' = \frac{c_0}{\tau _2}-\left( \frac{\alpha \kappa \tau _1}{2c_0^2\tau _2}\right) \Sigma ^2. \end{aligned}$$

Then *F* solves (5.12) if and only if

5.15 $$\begin{aligned} F(X) = F(0)e^{{{\,\mathrm{P}\,}}(0)-{{\,\mathrm{P}\,}}(X)}+\sigma (X)\int _0^X e^{{{\,\mathrm{P}\,}}(W)}\big (q_*G(W)+G'(W)\big ) \ dW. \end{aligned}$$

In particular, any solution *H* to $\mathcal {L}{H} = 0$ must be a scalar multiple of $e^{-{{\,\mathrm{P}\,}}(\cdot )}$, and so, by (5.13), $\sigma$ is also a scalar multiple of $e^{-{{\,\mathrm{P}\,}}(\cdot )}$. Consequently, we can rewrite (5.15) as

5.16 $$\begin{aligned} F(X) = \frac{F(0)}{\sigma (0)}\sigma (X)+\sigma (X)\int _0^X \frac{q_*G(W)+G'(W)}{\sigma (W)} \ dW. \end{aligned}$$

Conversely, if *F* satisfies (5.16), then we may undo all of the work above to see that $f := -F'$ solves $\mathcal {T}{f} = g$. Using the identities (5.8), we can recast this result in terms of the original functions *f* and *g*.

#### Lemma 5.2

For $$g \in H_q^1$$, define

5.17 $$\begin{aligned} (\mathcal {H}{g})(X) := q_*(\mathcal {A}{g})(X) - g(X) \end{aligned}$$

and

5.18 $$\begin{aligned} (\mathcal {K}{g})(X) := \sigma (X)\int _0^X \frac{(\mathcal {H}{g})(W)}{\sigma (W)} \ dW. \end{aligned}$$

Then $f \in H_q^1$ satisfies $\mathcal {T}{f} = g$ if and only if

5.19 $$\begin{aligned} (\mathcal {A}{f})(X) = \frac{(\mathcal {A}{f})(0)}{\sigma (0)}\sigma (X) + (\mathcal {K}{g})(X). \end{aligned}$$

In particular, a function $f \in H_{q,0}^1$ satisfies $\mathcal {T}{f} = g$ if and only if

5.20 $$\begin{aligned} (\mathcal {A}{f})(X) = (\mathcal {K}{g})(X). \end{aligned}$$

The identity (5.20) allows to prove the bijectivity of $\mathcal {T}:H_{q,0}^1 \rightarrow H_q^1$. The proof of injectivity is very easy. If $\mathcal {T}{f} = 0$ for some $f \in H_{q,0}^1$, then (5.20) implies $\mathcal {A}{f} = 0$, and so the identity (5.5) gives $f=0$. Observe that if we were working on all of $H_q^1$, then (5.19) tells us that $\mathcal {T}$ would have a one-dimensional kernel in $H_q^1$ spanned by $\sigma '$. But since $(\mathcal {A}\sigma ')(0) = -\sigma (0) \ne 0$, we have $\sigma ' \not \in H_{q,0}^1$.

Toward surjectivity, suppose $\mathcal {T}{f} = g$ for some $f \in H_{q,0}^1$ and $g \in H_q^1$. Then (5.5) and (5.20) imply

5.21 $$\begin{aligned} f = -\partial _X[\mathcal {K}{g}] =: \mathcal {S}{g}. \end{aligned}$$

That is, we expect $\mathcal {T}^{-1}=\mathcal {S}$. Now we make this rigorous.

#### Lemma 5.3

The operator $\mathcal {S}$, defined in (5.21), is a bounded linear operator from $H_q^1$ to $H_{q,0}^1$ that satisfies $\mathcal {T}\mathcal {S}{g} = g$ for all $g \in H_q^1$.

#### Proof

Let $g \in H_q^1$. In part (i) of Lemma 5.4 below we show that $\sigma ' = -\rho \sigma$ for a certain function $\rho \in L^{\infty }$. Then the definition of $\mathcal {K}$ in (5.18) gives

5.22 $$\begin{aligned} \mathcal {S}{g} = \rho (\mathcal {K}{g})-\mathcal {H}{g}, \end{aligned}$$

and the definition of $\mathcal {H}$ in (5.17) shows

5.23 $$\begin{aligned} \partial _X[\mathcal {S}{g}] = \rho '(\mathcal {K}{g})-\rho (\mathcal {S}{g})+q_*g+g'. \end{aligned}$$

We claim there is a constant *C*, independent of *g*, such that

5.24 $$\begin{aligned} \Vert \rho '(\mathcal {K}{g})\Vert _{L_q^2} \le C\Vert g\Vert _{H_q^1} \end{aligned}$$

and

5.25 $$\begin{aligned} \Vert \mathcal {S}{g}\Vert _{L_q^2} \le C\Vert g\Vert _{H_q^1} \end{aligned}$$

Since $\rho \in L^{\infty }$, the identities (5.22) and (5.23) show that $\mathcal {S}$ is a bounded operator on $H_q^1$. We prove the estimate (5.24) in Sect. 5.2.1 below and the estimate (5.25) in Sect. 5.2.2.

To show both that $\mathcal {S}{g} \in H_{q,0}^1$ and that taking $f=\mathcal {S}{g}$ satisfies (5.20), we first use the definition of $\mathcal {A}$ in (5.4) to compute

5.26 $$\begin{aligned} (\mathcal {A}\mathcal {S}{g})(X) = (\mathcal {K}{g})(X)-\lim _{B \rightarrow \infty } (\mathcal {K}{g})(B). \end{aligned}$$

We claim that

5.27 $$\begin{aligned} \lim _{B \rightarrow \infty } (\mathcal {K}{g})(B) = 0. \end{aligned}$$

Indeed, since $\mathcal {S}{g} \in H_q^1$, we know that $\mathcal {S}{g}$ vanishes at infinity; so does $\mathcal {H}{g}$ by the definition of $\mathcal {H}$ in (5.17). However, $\rho (X) \rightarrow q_* \ne 0$ as $X \rightarrow \infty$ by part (iii) of Lemma 5.4 below. The first equality in (5.22) then forces the limit (5.27) to be true.

Thus

5.28 $$\begin{aligned} (\mathcal {A}\mathcal {S}{g})(X) = (\mathcal {K}{g})(X). \end{aligned}$$

In particular,

$$\begin{aligned} (\mathcal {A}\mathcal {S}{g})(0) = (\mathcal {K}{g})(0) = 0 \end{aligned}$$

by the definition of $\mathcal {K}$ in (5.18). Consequently, $\mathcal {S}{g} \in H_{q,0}^1$, and so (5.28) shows that $f=\mathcal {S}{g}$ satisfies (5.20). This implies $\mathcal {T}(\mathcal {S}{g}) = g$. $\square$

### Auxiliary results for the proof of Lemma 5.3

We first study some properties of $\sigma$ and its derivative.

#### Lemma 5.4

(i) There exists $\rho \in L^{\infty }$ such that $\sigma ' = -\rho \sigma$.
(ii) There exist $\varsigma _1^+$, $\varsigma _2^+$, $\varrho ^+ \in L^{\infty }(\mathbb {R}_+)$, $\varsigma _1^-$, $\varsigma _2^-$, $\varrho ^- \in L^{\infty }(\mathbb {R}_-)$, and $C_1$, $C_2 \in \mathbb {R}$ such that
  5.29 $$\begin{aligned}&\frac{1}{\sigma (X)} = {\left\{ \begin{array}{ll} C_1e^{q_*X}+e^{-q_*X}\varsigma _1^+(X), \ X > 0 \\ C_2e^{-2q_*X}+\varsigma _1^-(X), \ X < 0, \end{array}\right. } \end{aligned}$$
  5.30 $$\begin{aligned}&\sigma (X) = {\left\{ \begin{array}{ll} C_1^{-1}e^{-q_*X}+e^{-3q_*X}\varsigma _2^+(X), \ X > 0 \\ C_2^{-1}e^{2q_*X}+e^{4q_*X}\varsigma _2^-(X), \ X < 0, \end{array}\right. } \end{aligned}$$
  and
  5.31 $$\begin{aligned} \rho (X) = {\left\{ \begin{array}{ll} q_*+e^{-q_*X}\varrho ^+(X), \ X > 0 \\ -2q_*+e^{q_*X}\varrho ^-(X), \ X < 0. \end{array}\right. } \end{aligned}$$
(iii) There is $C_3 > 0$ such that
  5.32 $$\begin{aligned} |\rho '(X)| \le C_3e^{-q_*|X|} \end{aligned}$$
  for all $$X \in \mathbb {R}$$.

#### Proof

(i) Recall that $\sigma = -\Sigma '$, where $\Sigma$ satisfies the Bernoulli equation (3.26). That is,
  $$\begin{aligned} \sigma = -\Sigma ' = \frac{c_0}{\tau _2}\Sigma -\left( \frac{\alpha \kappa \tau _1}{6c_0^2\tau _2}\right) \Sigma ^3. \end{aligned}$$
  Then
  $$\begin{aligned} \sigma ' = \frac{c_0}{\tau _2}\Sigma '-\left( \frac{\alpha \kappa \tau _1}{2c_0^2\tau _2}\right) \Sigma ^2\Sigma ' = \left[ \left( \frac{\alpha \kappa \tau _1}{2c_0^2\tau _2}\right) \Sigma ^2-\frac{c_0}{\tau _2}\right] \sigma . \end{aligned}$$
  Put
  5.33 $$\begin{aligned} \rho =\frac{c_0}{\tau _2}-\left( \frac{\alpha \kappa \tau _1}{2c_0^2\tau _2}\right) \Sigma ^2. \end{aligned}$$
  By the definition of $\Sigma$ in (3.27), we have $\rho \in L^{\infty }$. Note, incidentally, that $\rho$ must be a scalar multiple of ${{\,\mathrm{P}\,}}$ from (5.14).
(ii) The expansions (5.30) and (5.29) follow directly from the formula for $\sigma$ in (3.28). The expansion (5.31) follows from the definition of $\rho$ in (5.33) and the definition of $\Sigma$ in (3.27), which gives
  $$\begin{aligned} \lim _{X \rightarrow \infty } \Sigma (X)^2 = 0 \qquad \text { and }\qquad \lim _{X \rightarrow -\infty } \Sigma (X)^2 = \frac{3c_0}{\tau _2} = 3q_*. \end{aligned}$$
(iii) This is a direct consequence of (5.33).

$$\square $$

We will also need estimates on $\mathcal {A}$ and $\mathcal {H}$.

#### Lemma 5.5

There is $C > 0$ such that

5.34 $$\begin{aligned} \Vert \mathcal {A}{g}\Vert _{L^{\infty }} + \Vert \mathcal {H}{g}\Vert _{L^{\infty }} \le C\Vert g\Vert _{H_q^1} \end{aligned}$$

for all $$g \in H_q^1$$.

#### Proof

We use the definition of $\mathcal {A}$ in (5.4) to bound

$$\begin{aligned} |(\mathcal {A}{g})(X)| \le \Vert f\Vert _{L^1} \le C\Vert f\Vert _{H_q^1} \end{aligned}$$

by the embedding of $H_q^1$ into $L^1$, which we discuss in Appendix A.3. The estimate for $\mathcal {H}$ then follows from the triangle inequality. $\square$

In the following we again recall that $0< q < q_*$.

#### The proof of the estimate (5.24)

We first use the definition of $\mathcal {K}$ in (5.18) and the estimates (5.32) on $\rho '$ and (5.34) on $\mathcal {H}{g}$ to bound

$$\begin{aligned} |\rho '(X)(\mathcal {K}{g})(X)| \le C\Vert g\Vert _{H_q^1}e^{-q_*|X|}\sigma (X)\int _0^X \frac{dW}{\sigma (W)}. \end{aligned}$$

If $X > 0$, we use the estimates (5.30) on $\sigma (X)$ and (5.29) on $1/\sigma (W)$ to bound

$$\begin{aligned} \sigma (X)\int _0^X \frac{dW}{\sigma (W)} \le Ce^{-q_*X}\int _0^X e^{q_*W} \ dW\le C. \end{aligned}$$

If $X < 0$, we use the negative versions of these estimates to bound

$$\begin{aligned} \sigma (X)\left| \int _0^X \frac{dW}{\sigma (W)}\right| \le Ce^{2q_*X}\int _X^0 e^{-2q_*W} \le C. \end{aligned}$$

We conclude

$$\begin{aligned} |\rho '(X)(\mathcal {K}{g})(X)| \le C\Vert g\Vert _{H_q^1}e^{-q_*|X|} \end{aligned}$$

for all *X*. Since $0< q < q_*$, this gives $\Vert \rho '(\mathcal {K}{g})\Vert _{L_q^2} \le C\Vert g\Vert _{H_q^1}$.

#### The proof of the estimate (5.25)

It suffices to find $C > 0$ such that for all $g \in H_q^1$, we have

$$\begin{aligned} \Vert \mathcal {S}{g}\Vert _{L_q^2(\mathbb {R}_+)} + \Vert \mathcal {S}{g}\Vert _{L_q^2(\mathbb {R}_-)} \le C\Vert g\Vert _{H_q^1}. \end{aligned}$$

We will rewrite $(\mathcal {S}{g})(X)$ in different ways for $X > 0$ and $X < 0$ to exploit the different decay rates of $\sigma$ at $+\infty$ and $-\infty$.

First suppose $X > 0$. The formula (5.22) for $\mathcal {S}{g}$, the formula (5.18) for $\mathcal {K}$ and the expansions in Lemma 5.4 allow us to write

$$\begin{aligned}&(\mathcal {S}{g})(X) = \big (C_1^{-1}q_*e^{-q_*X}+e^{-2q_*X}\varsigma _3^+(X)\big )\\&\quad \int _0^X \big (C_1e^{q_*W}+e^{-q_*W}\varsigma _1^+(W)\big )(\mathcal {H}{g})(W)\ dW-(\mathcal {H}{g})(X), \end{aligned}$$

where $\varsigma _3^+ \in L^{\infty }(\mathbb {R}_+)$. We expand this to give

$$\begin{aligned} (\mathcal {S}{g})(X) = \sum _{j=1}^4 (\mathcal {S}_j^+g)(X), \end{aligned}$$

where

$$\begin{aligned} (\mathcal {S}_1^+g)(X)&:= q_*e^{-q_*X}\int _0^X e^{q_*W}(\mathcal {H}{g})(W) \ dW- (\mathcal {H}{g})(X) \\ (\mathcal {S}_2^+g)(X)&:= C_1^{-1}q_*e^{-q_*X}\int _0^X e^{-q_*W}\varsigma _1^+(W)(\mathcal {H}{g})(W) \ dW\\ (\mathcal {S}_3^+g)(X)&:= C_1e^{-2q_*X}\varsigma _3^+(X)\int _0^X e^{q_*W}(\mathcal {H}{g})(W) \ dW\\ (\mathcal {S}_4^+g)(X)&:= e^{-2q_*X}\varsigma _3^+(X)\int _0^X e^{-q_*W}\varsigma _1^+(W)(\mathcal {H}{g})(W) \ dW. \end{aligned}$$

The estimate (5.34) from Lemma 5.5 allows us to bound the last three terms with

5.35 $$\begin{aligned} |(\mathcal {S}_2^+g)(X)| + |(\mathcal {S}_4^+g)(X)| \le Ce^{-q_*X}\Vert \mathcal {H}{g}\Vert _{L^{\infty }}\int _0^X e^{-q_*W} \le Ce^{-q_*X}\Vert g\Vert _{H_q^1}\nonumber \\ \end{aligned}$$

and

5.36 $$\begin{aligned} |(\mathcal {S}_3^+g)(X)| \le Ce^{-2q_*X}\Vert \mathcal {H}{g}\Vert _{L^{\infty }}\int _0^X e^{q_*W} \le Ce^{-q_*X}\Vert g\Vert _{H_q^1}. \end{aligned}$$

To control $\mathcal {S}_1^+g$, we first integrate by parts:

$$\begin{aligned} \int _0^X e^{q_*W}(\mathcal {H}{g})(W) \ dW= \frac{e^{q_*X}(\mathcal {H}{g})(X)-(\mathcal {H}{g})(0)}{q_*}-\frac{1}{q_*}\int _0^X e^{q_*W}(\mathcal {H}{g})'(W) \ dW. \end{aligned}$$

The definition of $\mathcal {H}$ in (5.17) gives

5.37 $$\begin{aligned}&\int _0^X e^{q_*W}(\mathcal {H}{g})'(W) \ dW\nonumber \\&\qquad = -\int _0^X e^{q_*W}(q_*g(W)+g'(W)) \ dW= -\int _0^X \partial _W[e^{q_*W}g(W)] \ dW\nonumber \\&\qquad = g(0)-e^{q_*X}g(X). \end{aligned}$$

It follows that

5.38 $$\begin{aligned} (\mathcal {S}_1^+g)(X) = -e^{-q_*X}(\mathcal {H}{g})(0) -e^{-q_*X}g(0) +g(X). \end{aligned}$$

We apply Lemma 5.5 to the factor $(\mathcal {H}{g})(0)$ and use the Sobolev embedding to estimate *g*(0), so that

$$\begin{aligned} |(\mathcal {S}_1^+g)(X)| \le Ce^{-q_*X}\Vert g\Vert _{H_q^1}+|g(X)|. \end{aligned}$$

Since $0< q < q_*$, the estimates (5.35) and (5.36) and the identity (5.38) give

$$\begin{aligned} e^{qX}|(\mathcal {S}{g})(X)| \le Ce^{(q-q_*)X}\Vert g\Vert _{H_q^1} + e^{qX}|g(X)| \end{aligned}$$

for $X > 0$, from which the bound

$$\begin{aligned} \Vert \mathcal {S}{g}\Vert _{L_q^2(\mathbb {R}_+)} \le C\Vert g\Vert _{H_q^1} \end{aligned}$$

follows.

Now suppose $X < 0$. Using the expansions in Lemma 5.4 valid for $X < 0$, we rewrite

$$\begin{aligned}&(\mathcal {S}{g})(X) = \big (2q_*C_2^{-1}e^{2q_*X}+e^{3q_*X}\varsigma _3^-(X)\big )\int _X^0 \big (C_2e^{-2q_*W}+\varsigma _1^-(W)\big )(\mathcal {H}{g})(W) \\&\quad - (\mathcal {H}{g})(X), \end{aligned}$$

where $\varsigma _3^- \in L^{\infty }(\mathbb {R}_-)$. We expand this as

$$\begin{aligned} (\mathcal {S}{g})(X) = \sum _{j=1}^4 (\mathcal {S}_j^-g)(X), \end{aligned}$$

where

$$\begin{aligned} (\mathcal {S}_1^-g)(X)&:= 2q_*e^{2q_*X}\int _X^0 e^{-2q_*W}(\mathcal {H}{g})(W) \ dW- (\mathcal {H}{g})(X) \\ (\mathcal {S}_2^-g)(X)&:= 2q_*C_2^{-1}e^{2q_*X}\int _X^0 \varsigma _1^-(W)(\mathcal {H}{g})(W) \ dW\\ (\mathcal {S}_3^-g)(X)&:= C_2e^{3q_*X}\varsigma _3^-(X)\int _X^0 e^{-2q_*W} (\mathcal {H}{g})(W) \ dW\\ (\mathcal {S}_4^-g)(X)&:= e^{3q_*X}\varsigma _3^-(X)\int _X^0 \varsigma _1^-(W)(\mathcal {H}{g})(W) \ dW. \end{aligned}$$

We crudely estimate the last three terms as

5.39 $$\begin{aligned} |(\mathcal {S}_2^-g)(X)| + |(\mathcal {S}_3^-g)(X)| + |(\mathcal {S}_4^-g)(X)|&\le Ce^{2q_*X}|X|\Vert \mathcal {H}{g}\Vert _{L^{\infty }} \nonumber \\&\le Ce^{q_*X}\Vert g\Vert _{H_q^1}. \end{aligned}$$

For the first term, we integrate by parts to find

$$\begin{aligned} \int _X^0 e^{-2q_*W}(\mathcal {H}{g})(W) \ dW= & {} \frac{e^{-2q_*X}(\mathcal {H}{g})(X)-(\mathcal {H}{g})(0)}{2r} \\&- \frac{1}{2q_*}\int _X^0 e^{-2q_*W}(q_*g(W)+g'(W)) \ dW. \end{aligned}$$

The difference compared to (5.37) in our treatment of $\mathcal {S}_1^+g$ is that we no longer have a perfect derivative as the integrand on the right; this is an consequence of the different asymptotic behavior of $\rho$ and $\sigma$ at $-\infty$ compared to $+\infty$, as specified in Lemma 5.4. Thus

5.40 $$\begin{aligned} (\mathcal {S}_1^-g)(X) = -(\mathcal {H}{g})(0)e^{2q_*X}+\mathcal {I}[g](X), \end{aligned}$$

where

$$\begin{aligned} \mathcal {I}[g](X) := -e^{2q_*X}\int _X^0 e^{-2q_*W}\big (rg(W)+g'(W)\big ) \ dW. \end{aligned}$$

To control this integral term, we will use the following lemma, whose proof we defer to Sect. 5.2.3.

##### Lemma 5.6

There exists $C > 0$ such that

5.41 $$\begin{aligned} \int _{-\infty }^0 e^{2X}\left| \int _X^0 e^{-W}h(W) \ dW\right| ^2 \ dX\le C\Vert h\Vert _{L^2} \end{aligned}$$

for all $$h \in L^2$$.

Since $q_*g+g' \in L_q^2$, we can write

$$\begin{aligned} q_*g(X)+g'(X) = e^{-q|X|}h(X) \end{aligned}$$

for some $h \in L^2$. Then

$$\begin{aligned} \int _{-\infty }^0 e^{-2qX}|\mathcal {I}[g](X)|^2 \ dX= & {} \int _{-\infty }^0 e^{2(2q_*-q)X}\left| \int _X^0 e^{-(2q_*-q)W}h(W) \ dW\right| ^2 \ dX\\= & {} \frac{1}{(2q_*-q)^2}\int _{-\infty }^0 e^{2U}\left| \int _U^0 e^{-V}h\left( \frac{V}{2q_*-q}\right) \ dV\right| ^2 \ dU. \end{aligned}$$

Applying Lemma 5.6, we obtain

$$\begin{aligned} \Vert \mathcal {I}[g]\Vert _{L_q^2(\mathbb {R}_-)} \le C\left\| h\left( \frac{\cdot }{2q_*-q}\right) \right\| _{L^2} \le C\Vert e^{-q|\cdot |}(e^{q|\cdot |}h)\Vert _{L^2} \le C\Vert g\Vert _{H_q^1}. \end{aligned}$$

All together, we use the estimates (5.39) and the identity (5.40) to bound

$$\begin{aligned} |(\mathcal {S}{g})(X)| \le Ce^{q_*X}\Vert g\Vert _{H_q^1} + |\mathcal {I}[g](X)|, \end{aligned}$$

from which we obtain

$$\begin{aligned} \Vert \mathcal {S}{g}\Vert _{L_q^2(\mathbb {R}_-)} \le C\Vert g\Vert _{H_q^1}. \end{aligned}$$

#### The proof of Lemma 5.6

Put

$$\begin{aligned} \mathcal {W}:= \!\left\{ (X,W,Y) \in \mathbb {R}^3 \ | \ -\infty < X \le 0, \ X \le W \le 0, \ X \le Y \le 0\right\} , \end{aligned}$$

so that, after using the triangle inequality, the integral in (5.41) is bounded by

$$\begin{aligned} \mathcal {J}:= & {} \int _{-\infty }^0 e^{2X}\left( \int _X^0 e^{-W}|h(W)| \ dW\right) ^2 \ dX\\= & {} \iiint _{\mathcal {W}} e^{2X}e^{-W}e^{-Y}|h(W)h(Y)|\ dY\ dW\ dX. \end{aligned}$$

Next, put

$$\begin{aligned} \mathcal {W}_1 := \!\left\{ (X,W,Y) \in \mathbb {R}^3 \ | \ -\infty< X \le W, \ W \le Y \le 0, \ -\infty < W \le 0\right\} \end{aligned}$$

and

$$\begin{aligned} \mathcal {W}_2 := \!\left\{ (X,W,Y) \in \mathbb {R}^3 \ | \ -\infty< X \le Y, \ Y \le W \le 0, \ -\infty < Y \le 0\right\} , \end{aligned}$$

so $\mathcal {W}= \mathcal {W}_1 \cup \mathcal {W}_2$ and $\mathcal {W}_1 \cap \mathcal {W}_2$ has measure zero. Then

$$\begin{aligned} \mathcal {J}= \mathcal {J}_1 + \mathcal {J}_2, \end{aligned}$$

where

$$\begin{aligned} \mathcal {J}_1 := \iiint _{\mathcal {W}_1} e^{2X}e^{-W}e^{-Y}|h(W)h(Y)|\ dY\ dW\ dX\end{aligned}$$

and

$$\begin{aligned} \mathcal {J}_2 := \iiint _{\mathcal {W}_2} e^{2X}e^{-W}e^{-Y}|h(W)h(Y)|\ dY\ dW\ dX. \end{aligned}$$

Since the integrands are symmetric in *W* and *Y*, it suffices to show

$$\begin{aligned} \mathcal {J}_1 \le C\int _{-\infty }^0 |h(X)|^2 \ dX. \end{aligned}$$

Change variables to obtain

$$\begin{aligned} \mathcal {J}_1= & {} \int _{-\infty }^0\int _W^0\left( \int _{-\infty }^W e^{2X} \ dX\right) e^{-W}e^{-Y} |h(W)h(Y)| \ dY\ dW\\= & {} \frac{1}{2}\int _{-\infty }^0\int _W^0 e^We^{-Y} |h(W)h(Y)| \ dY\ dW. \end{aligned}$$

Now we estimate

5.42 $$\begin{aligned} 4|\mathcal {J}_1| \le \mathcal {J}_{12} + \mathcal {J}_{13}, \end{aligned}$$

where

$$\begin{aligned} \mathcal {J}_{12}&:= \int _{-\infty }^0\int _W^0 e^{W}e^{-Y}|h(W)|^2 \ dY\ dW\qquad \text { and }\qquad \\ \mathcal {J}_{13}&:= \int _{-\infty }^0\int _W^0 e^{W}e^{-Y}|h(Y)|^2 \ dY\ dW. \end{aligned}$$

We first evaluate

$$\begin{aligned} \mathcal {J}_{12} = \int _{-\infty }^0 \left( \int _W^0 e^{-Y} \ dY\right) e^W|h(W)|^2 \ dW= \int _{-\infty }^0 (1-e^W)|h(W)|^2 \ dW. \end{aligned}$$

Since $W \le 0$ we have $|1-e^W| \le 2$, and so

5.43 $$\begin{aligned} \mathcal {J}_{12} \le 2\int _{-\infty }^0 |h(W)|^2 \ dW\le C\Vert h\Vert _{L^2}^2. \end{aligned}$$

Next, we change variables in $\mathcal {J}_{13}$ to find

5.44 $$\begin{aligned} \mathcal {J}_{13} = \int _{-\infty }^0 \left( \int _{-\infty }^{Y} e^{W} \ dW\right) e^{-Y}|h(Y)|^2 \ dY= \int _{-\infty }^0 |h(Y)|^2 \ dY\le \Vert h\Vert _{L^2}^2. \nonumber \\ \end{aligned}$$

Combining the decomposition (5.42) and the estimates (5.43) and (5.44) gives

$$\begin{aligned} |\mathcal {J}_1| \le C\Vert h\Vert _{L^2}^2, \end{aligned}$$

as desired.

## Analysis of the long wave problem

### The perturbation Ansatz for the long wave problem (3.42)

Throughout this section we keep $q \in (0,c_0/\tau _2)$ fixed. We make the perturbation Ansatz

6.1 $$\begin{aligned} \psi _1 = \sigma + \eta _1 \qquad \text { and }\qquad \psi _2 = \zeta + \eta _2 \end{aligned}$$

for the long wave problem (3.42). Here $\eta _1 \in H_{q,0}^1$, which was defined in (5.2), and $\eta _2 \in W^{1,\infty }$. We abbreviate

$$\begin{aligned} \varvec{\eta }= (\eta _1,\eta _2) \in \mathcal {X}:= H_{q,0}^1 \times W^{1,\infty }, \end{aligned}$$

where $\mathcal {X}$ has the norm

$$\begin{aligned} \Vert \varvec{\eta }\Vert _{\mathcal {X}} := \Vert \eta _1\Vert _{H_q^1} + \Vert \eta _2\Vert _{W^{1,\infty }}. \end{aligned}$$

The Ansatz (6.1) solves the system (3.42) if and only if $\eta _1$ and $\eta _2$ solve

6.2 $$\begin{aligned} {\left\{ \begin{array}{ll} \mathcal {T}\eta _1 = \sum _{k=1}^5 \mathcal {V}_{1k}^{\nu }(\varvec{\eta }), \\ \eta _2 = \sum _{k=1}^3 \mathcal {V}_{2k}^{\nu }(\varvec{\eta }), \end{array}\right. } \end{aligned}$$

where $\mathcal {T}$ was defined in (3.43) and the $\mathcal {V}$-operators are given by

6.3 $$\begin{aligned} \begin{aligned} \mathcal {V}_{11}^{\nu }(\varvec{\eta })&:= \big (\mathcal {M}^{(\nu )}-\mathcal {M}^{(0)}\big )\big [\mathcal {R}_1^{\nu }(\sigma +\eta _1)(\sigma +\eta _1)\big ], \\ \mathcal {V}_{12}^{\nu }(\varvec{\eta })&:= \mathcal {M}^{(0)}\big [\big (\mathcal {R}_1^{\nu }(\sigma +\eta _1)-\mathcal {R}_1^0(\sigma +\eta _1)\big )(\sigma +\eta _1)\big ], \\ \mathcal {V}_{13}^{\nu }(\varvec{\eta })&:= \mathcal {M}^{(0)}\big [\big (\mathcal {R}_1^0(\sigma +\eta _1)-\mathcal {R}_1^0(\sigma )-D\mathcal {R}_1^0(\sigma )\eta _1\big )\sigma \big ], \\ \mathcal {V}_{14}^{\nu }(\varvec{\eta })&:= \mathcal {M}^{(0)}\big [\big (\mathcal {R}_1^0(\sigma +\eta _1)-\mathcal {R}_1^0(\sigma )\big )\eta _1\big ], \\ \mathcal {V}_{15}^{\nu }(\varvec{\eta })&:= \nu ^{1/2}\mathcal {M}^{(0)}\mathcal {N}^{\nu }(\sigma +\eta _1,\zeta +\eta _2) \end{aligned} \end{aligned}$$

and

6.4 $$\begin{aligned} \begin{aligned} \mathcal {V}_{21}^{\nu }(\varvec{\eta })&:= \mathcal {P}_1^{\nu }(\sigma +\eta _1)-\mathcal {P}_1^0(\sigma +\eta _1), \\ \mathcal {V}_{22}^{\nu }(\varvec{\eta })&:= \mathcal {P}_1^0(\sigma +\eta _1)-\zeta , \\ \mathcal {V}_{23}^{\nu }(\varvec{\eta })&:= \nu \mathcal {P}_2^{\nu }(\sigma +\eta _1,\zeta +\eta _2). \end{aligned} \end{aligned}$$

We recall that the symbol of $\mathcal {M}^{(\nu )}$ was defined in (3.40) and the symbol of $\mathcal {M}^{(0)}$ in (3.21). The operator $\mathcal {R}_1^{\nu }$ was defined in (3.35), the operator $\mathcal {N}^{\nu }$ in (3.38), the operator $\mathcal {P}_1^{\nu }$ in (3.33) and the operator $\mathcal {P}_2^{\nu }$ in (3.34).

Due to Proposition 5.1, the first equation in (6.2) is equivalent to

6.5 $$\begin{aligned} \eta _1 = \mathcal {T}^{-1}\sum _{k=1}^5 \mathcal {V}_{1k}^{\nu }(\varvec{\eta }) =: \mathfrak {N}_1^{\nu }(\varvec{\eta }). \end{aligned}$$

Subsequently, $\eta _1$ and $\eta _2$ solve (6.2) if and only if

6.6 $$\begin{aligned} \eta _2 = \mathcal {V}_{21}^{\nu }(\varvec{\eta }) + \mathcal {V}_{22}^{\nu }(\mathfrak {N}_1^{\nu }(\varvec{\eta })) + \mathcal {V}_{23}^{\nu }(\varvec{\eta }) = : \mathfrak {N}_2^{\nu }(\varvec{\eta }). \end{aligned}$$

We have replaced $\eta _1$ with its fixed point expression (6.5) in $\mathcal {V}_{22}^{\nu }$ for the sake of better estimates later; see Appendix B.2.7 for a more precise discussion. Finally, set

6.7 $$\begin{aligned} \varvec{\mathfrak {N}}^{\nu }(\varvec{\eta }) := (\mathfrak {N}_1^{\nu }(\varvec{\eta }),\mathfrak {N}_2^{\nu }(\varvec{\eta })), \end{aligned}$$

so $\varvec{\mathfrak {N}}^{\nu }$ maps $\mathcal {X}$ to $\mathcal {X}$. More precisely, this follows from the mapping estimates in Appendix B.3. We conclude that the problem (6.2) is equivalent to the fixed point problem

6.8 $$\begin{aligned} \varvec{\eta }= \varvec{\mathfrak {N}}^{\nu }(\varvec{\eta }), \end{aligned}$$

which we now solve.

### The solution of the fixed point problem (6.8)

For $r > 0$, we define the ball

$$\begin{aligned} \mathfrak {B}(r) := \!\left\{ \varvec{\eta }\in \mathcal {X} \ | \ \Vert \varvec{\eta }\Vert _{\mathcal {X}} \le r\right\} . \end{aligned}$$

We prove the following estimates in Appendix B; their verifications are routine, but detailed, so we do not present them here.

#### Proposition 6.1

There exist $C_{\star }$, $\nu _{\star } > 0$ such that if $0< \nu < \nu _{\star }$ then the following hold.

(i) If $$\varvec{\eta }\in \mathfrak {B}(C_{\star }\nu ^{1/3})$$, then $$\varvec{\mathfrak {N}}^{\nu }(\varvec{\eta }) \in \mathfrak {B}(C_{\star }\nu ^{1/3})$$.
(ii) If $\varvec{\eta }$, $\grave{\varvec{\eta }} \in \mathfrak {B}(C_{\star }\nu ^{1/3})$, then
  $$\begin{aligned} \Vert \varvec{\mathfrak {N}}^{\nu }(\varvec{\eta })-\varvec{\mathfrak {N}}^{\nu }(\grave{\varvec{\eta }})\Vert _{\mathcal {X}} \le \frac{1}{2}\Vert \varvec{\eta }-\grave{\varvec{\eta }}\Vert _{\mathcal {X}}. \end{aligned}$$

Proposition 6.1 then guarantees that $\varvec{\mathfrak {N}}^{\nu }$ is a contraction on $\mathfrak {B}(C_{\star }\nu ^{1/3})$ for each $0< \nu < \nu _{\star }$, and so Banach’s fixed point theorem gives the following solution to (6.2).

#### Proposition 6.2

Let $C_{\star }$, $\nu _{\star } > 0$ be as in Proposition 6.1. For each $0< \nu < \nu _{\star }$, there exists a unique $\varvec{\eta }^{\nu } \in \mathfrak {B}(C_{\star }\nu ^{1/3})$ such that $\varvec{\eta }^{\nu } = \varvec{\mathfrak {N}}^{\nu }(\varvec{\eta }^{\nu })$.

The existence of the perturbation terms $\varvec{\eta }^{\nu }$ enables us to conclude our main results, which are paraphrased nontechnically in (1.5).

#### Theorem 6.3

Let $\alpha$, $\kappa$, $\tau _1$, $\tau _2 > 0$, $q \in (0,c_0/\tau _2)$, and $\theta \in \mathbb {R}$. Define the leading-order profile terms

$$\begin{aligned}&\phi _A^*(X) := \left( \frac{6\sqrt{6}c_0^{9/2}}{\tau _2}\right) \left( \frac{\exp (2c_0X/\tau _2+\theta )}{\big [\alpha \kappa \tau _1+6c_0^2\exp (2c_0X/\tau _2+\theta )\big ]^{3/2}}\right) , \\&\phi _P^*(X) := \big ((6c_0)^{1/2}\alpha \big )\left( \frac{1}{\big [\alpha \kappa \tau _1+6c_0^2\exp \big (2c_0X/\tau _2+\theta )/\tau _2\big )\big ]^{1/2}}\right) , \end{aligned}$$

and

$$\begin{aligned} \phi _R^*(X) := (3\alpha \kappa {c}_0)\left( \frac{1}{\alpha \kappa \tau _1+6c_0^2\exp (2c_0X/\tau _2+\theta )/\tau _2)}\right) . \end{aligned}$$

There exists $\epsilon _{\star } > 0$ such that for each $0< \epsilon < \epsilon _{\star }$, there are $\phi _A^{\epsilon } \in H_q^1 \cap \mathcal {C}^{\infty }$ and $\phi _P^{\epsilon }$, $\phi _R^{\epsilon } \in W^{1,\infty } \cap \mathcal {C}^{\infty }$ with the following properties.

(i) Let
  $$\begin{aligned}&A_j(t) = \epsilon \phi _A^*(\epsilon ^{2/5}(j-\epsilon ^{2/5}{c}_0t)) + \epsilon ^{17/15}\phi _A^{\epsilon }(\epsilon ^{2/5}(j-\epsilon ^{2/5}c_0t)), \\&P_j(t) = \epsilon ^{1/5}\phi _P^*(\epsilon ^{2/5}(j-\epsilon ^{2/5}c_0t))+\epsilon ^{1/3}\phi _P^{\epsilon }(\epsilon ^{2/5}(j-\epsilon ^{2/5}c_0t)), \end{aligned}$$
  and
  $$\begin{aligned} R_j(t) = \epsilon ^{2/5}\phi _R^*(\epsilon ^{2/5}(j-\epsilon ^{2/5}c_0t)) + \epsilon ^{3/5}\phi _R^{\epsilon }(\epsilon ^{2/5}(j-\epsilon ^{2/5}c_0t)). \end{aligned}$$
  Then the triple $(A_j,P_j,R_j)$ solves (1.1).
(ii) The remainder terms $\phi _A^{\epsilon }$, $\phi _P^{\epsilon }$, and $\phi _R^{\epsilon }$ satisfy
  $$\begin{aligned} \sup _{0< \epsilon< \epsilon _{\star }} \Vert \phi _A^{\epsilon }\Vert _{H_q^1} + \Vert \phi _P^{\epsilon }\Vert _{W^{1,\infty }} + \Vert \phi _R^{\epsilon }\Vert _{W^{1,\infty }} < \infty . \end{aligned}$$
(iii) The functions $\phi _P^{\epsilon }$ and $\phi _R^{\epsilon }$ vanish exponentially fast at $+\infty$ and are asymptotically constant at $-\infty$ in the following sense: there exist $\ell _P^{\epsilon }$, $\ell _R^{\epsilon } \in \mathbb {R}$ such that
  $$\begin{aligned} \sup _{0< \epsilon< \epsilon _{\star }} \left( |\ell _P^{\epsilon }| + \sup _{X \ge 0} e^{qX}|\phi _P^{\epsilon }(X)| + \sup _{X \le 0} e^{-qX}|\phi _P^{\epsilon }(X)-\ell _P^{\epsilon }|\right) < \infty \end{aligned}$$
  and
  $$\begin{aligned} \sup _{0< \epsilon< \epsilon _{\star }} \left( |\ell _R^{\epsilon }| + \sup _{X \ge 0} e^{qX}|\phi _R^{\epsilon }(X)| + \sup _{X \le 0} e^{-qX}|\phi _R^{\epsilon }(X)-\ell _R^{\epsilon }|\right) < \infty . \end{aligned}$$

#### Proof

Write the solution of (6.8), which exists due to Proposition 6.2, as $\varvec{\eta }^{\nu } = (\eta _1^{\nu },\eta _2^{\nu })$. Define

6.9 $$\begin{aligned} \psi _1^{\nu } := \sigma + \eta _1^{\nu } \qquad \text { and }\qquad \psi _2^{\nu } := \zeta + \eta _2^{\nu }. \end{aligned}$$

By the discussion at the start of Sect. 6.1, the pair $(\psi _1^{\nu },\psi _2^{\nu })$ then solves the system (3.42).

Now take

$$\begin{aligned} A_j(t) = \nu ^{5/2}\psi _1^{\nu }(\nu (j-\nu {c}_0t)) \qquad \text { and }\qquad P_j(t) = \nu ^{1/2}\psi _2^{\nu }(\nu (j-\nu {c}_0t)). \end{aligned}$$

This is the scaled travelling wave ansatz from (3.41), and so Proposition 3.5 guarantees that $A_j$ and $P_j$ thus defined solve the simplified system (2.14). Let $R_j$ be given by (2.12). Then the discussion in Sect. 2.1 shows that $A_j$, $P_j$ and $R_j$ together solve our original problem (1.1).

Next, we use the identity $\epsilon = \nu ^{5/2}$ from (3.30) to reintroduce the original long wave parameter $\epsilon$ into the solutions. The expansions in (i) and the estimates in (ii) above then follow from (6.9) and the estimates in Proposition 6.2. The exact formulas for the leading order terms follow from the definitions of $\sigma$ in (3.28) and $\zeta$ in (3.29). The asymptotics of part (iii) follow from the definition of $\zeta$, the fixed point property $\eta _2^{\nu } = \mathfrak {N}_2^{\nu }(\varvec{\eta }^{\nu })$ and the definition of $\mathfrak {N}_2^{\nu }$ in (6.6), and the definition of $R_j$ from (2.12).

Finally, to obtain the smoothness of the solutions, we first note that $\sigma$, $\zeta \in \mathcal {C}^{\infty }$. Next, crude estimates on the symbol of the Fourier multiplier $\mathcal {M}^{(\nu )}$, which we omit, allow us to invoke Lemma A.2 to conclude $\mathcal {M}^{(\nu )} \in \mathbf {B}(H_q^r,H_q^{r+1})$ for each $\nu \ge 0$ and $r \ge 0$; we make no claim about uniform estimates in $\nu$ here. This smoothing property of $\mathcal {M}^{(\nu )}$, as well as the smoothing properties of the integral operators that compose $\mathfrak {N}_2^{\nu }$, show that if $\varvec{\eta }\in \mathcal {C}^r \times \mathcal {C}^2$, then $\varvec{\mathfrak {N}}^{\nu }(\varvec{\eta }) \in \mathcal {C}^{r+1} \times \mathcal {C}^{r+1}$. By bootstrapping, we obtain $\varvec{\eta }^{\nu } \in \mathcal {C}^{\infty } \times \mathcal {C}^{\infty }$. $\square$

In order to achieve the normalization $\Vert \phi _A^*\Vert _{L^{\infty }} = 1$, as discussed in Sect. 1.4, we need to use the explicit choice

6.10 $$\begin{aligned} c_0 = \left( \frac{9\alpha \kappa \tau _1\tau _2^2}{8}\right) ^{1/5} =: c_*, \end{aligned}$$

as employed in (1.5). From (6.10), we obtain

6.11 $$\begin{aligned} \Vert \phi _P^*\Vert _{L^{\infty }} = \left( \frac{6\alpha }{\kappa \tau _1}\right) ^{1/2}\left( \frac{9\alpha \kappa \tau _1\tau _2^2}{8}\right) ^{1/10} \qquad \text { and }\qquad \Vert \phi _R^*\Vert _{L^{\infty }} = \frac{3}{\tau _1}\left( \frac{9\alpha \kappa \tau _1\tau _2^2}{8}\right) ^{1/5}. \nonumber \\ \end{aligned}$$

Substituting the abbreviations (2.2) and (2.3) into the quantities in (6.10) and (6.11) then leads to the identities (1.8). This completes the rigorous derivation of the main results that we discussed more informally in Sect. 1.4.

### Supplementary Information

Below is the link to the electronic supplementary material.

Supplementary file 1 (mp4 4441 KB)

## Data Availability

The datasets generated during the current study are available from the corresponding author on reasonable request.

## Appendix A. Fourier analysis

### A.1. The Fourier transform

We use the following conventions for Fourier transforms. If $f \in L^1$, then its Fourier transform is

$$\begin{aligned} \mathfrak {F}[f](k) = {\widehat{f}}(k) := \frac{1}{\sqrt{2\pi }}\int _{-\infty }^{\infty } f(x)e^{-ikx} \ dx, \end{aligned}$$

and its inverse Fourier transform is

### A.2. Fourier multipliers on Sobolev spaces

For integers $r \ge 0$, we denote by $H^r = H^r(\mathbb {R})$ the usual Sobolev space of all *r*-times weakly differentiable functions whose weak derivatives are square-integrable.

Our fundamental operator on Sobolev spaces is the Fourier multiplier. The following result above is standard; see, e.g., Faver (2018, Lem. D.2.1).

#### Lemma A.1

Let $\widetilde{\mathcal {M}}:\mathbb {R}\rightarrow \mathbb {C}$ be measurable and suppose

$$\begin{aligned} N_{\widetilde{\mathcal {M}}}(r,s) := \sup _{k \in \mathbb {R}} \frac{|\widetilde{\mathcal {M}}(k)|}{(1+k^2)^{(r-s)/2}} < \infty . \end{aligned}$$

Then the **Fourier multiplier** $\mathcal {M}$ **with symbol** $\widetilde{\mathcal {M}}$ defined by

A.1 $$\begin{aligned} \mathcal {M}{f} := \mathfrak {F}^{-1}\big [\widetilde{\mathcal {M}}{\widehat{f}}\big ], \end{aligned}$$

i.e., by $\widehat{\mathcal {M}{f}}(k) = \widetilde{\mathcal {M}}(k){\widehat{f}}(k)$, is a bounded operator from $H^r$ to $H^s$, and

A.2 $$\begin{aligned} \Vert \mathcal {M}\Vert _{\mathbf {B}(H^r,H^s)} = N_{\widetilde{\mathcal {M}}}(r,s). \end{aligned}$$

We also need a convenient expression for ‘scaled’ Fourier multipliers. If *f* is a function on $\mathbb {R}$ and $\nu \in \mathbb {R}\setminus \{0\}$, let $f(\nu \cdot )$ be the ‘scaled’ map $x \mapsto f(\nu {x})$. Now let $\mathcal {M}$ be the Fourier multiplier with symbol $\widetilde{\mathcal {M}}$ and define $\widetilde{\mathcal {M}}^{(\nu )}(k) := \widetilde{\mathcal {M}}(\nu {k})$. Let $\mathcal {M}^{(\nu )}$ be the Fourier multiplier with symbol $\widetilde{\mathcal {M}}^{(\nu )}$. Then standard scaling properties of the Fourier transform imply that

A.3 $$\begin{aligned} \mathcal {M}[f(\nu \cdot )](x) = (\mathcal {M}^{(\nu )}f)(\nu {x}). \end{aligned}$$

### A.3. Fourier multipliers on weighted Sobolev spaces

We frequently work with weighted Sobolev spaces. For $q \in \mathbb {R}$, let

$$\begin{aligned} H_q^r := \!\left\{ f \in H_q^r \ | \ e^{q|\cdot |}f \in H^r\right\} . \end{aligned}$$

We norm this space by

$$\begin{aligned} \Vert f\Vert _{H_q^r} := \Vert e^{q|\cdot |}f\Vert _{H^r}, \end{aligned}$$

and, see Faver (2018, App. C), this norm is equivalent to

$$\begin{aligned} f \mapsto \sum _{j=0}^r \Vert e^{q|\cdot |}\partial _x^j[f]\Vert _{L^2}. \end{aligned}$$

We put $L_q^2 := H_q^0$. The Cauchy–Schwarz inequality guarantees that $L_q^2$ embeds into $L^1$:

$$\begin{aligned} \Vert f\Vert _{L^1} = \Vert e^{-q|\cdot |}(e^{q|\cdot |}f)\Vert _{L^1} \le \Vert e^{-|q\cdot |}\Vert _{L^2}\Vert e^{q|\cdot |}f\Vert _{L^2} \le C_q\Vert f\Vert _{L_q^2}. \end{aligned}$$

Finally, if $I \subseteq \mathbb {R}$ is an interval, we sometimes denote by $L_q^2(I)$ the set of all measurable functions $f :I \rightarrow \mathbb {C}$ such that

$$\begin{aligned} \int _I e^{2q|X|}|f(X)|^2 \ dX< \infty . \end{aligned}$$

Since $H_q^r \subseteq H^r$, any Fourier multiplier defined on $H^r$ is defined on $H_q^r$. A variation on a result of Beale (1980, Lem. 5.1) gives sufficient conditions for a Fourier multiplier on $H_q^r$ to map into another weighted space $H_q^s$.

#### Lemma A.2

(Beale) Fix $q > 0$ and let

$$\begin{aligned} \overline{\mathcal {U}}_q := \!\left\{ z \in \mathbb {C} \ | \ |{{\,\mathrm{Im}\,}}(z)| \le q\right\} . \end{aligned}$$

Let $\widetilde{\mathcal {M}}$ be analytic on $\overline{\mathcal {U}}_q$. Suppose there exist $s \ge 0$ and *C*, $r_0 > 0$ such that if $z \in \overline{\mathcal {U}}_q$ and $r_0 < |z|$, then

$$\begin{aligned} |\widetilde{\mathcal {M}}(z)| \le \frac{C}{|{{\,\mathrm{Re}\,}}(z)|^{(s-r)}}. \end{aligned}$$

Then the Fourier multiplier $\mathcal {M}$ with symbol $\widetilde{\mathcal {M}}$, defined by (A.1), is a bounded operator from $H_q^r$ to $H_q^s$ and

$$\begin{aligned} \Vert \mathcal {M}\Vert _{\mathbf {B}(H_q^r,H_q^s)} \le \sup _{k \in \mathbb {R}} \big |(1+k^2)^{(s-r)/2}\widetilde{\mathcal {M}}(k\pm {i}q)\big |. \end{aligned}$$

## Appendix B. The Proof of Proposition 6.1

Our proof depends on the following lemma, which we prove in the subsequent parts of this appendix.

### Lemma B.1

Let $\nu _{\mathcal {M}} > 0$ be as in Proposition 4.1. There exist $C_{\varvec{\mathfrak {N}}}$, $\rho _{\varvec{\mathfrak {N}}} > 0$ such that if $0< \nu < \nu _{\mathcal {M}}$ and $\Vert \eta _1\Vert _{H_q^1}$, $\Vert \grave{\eta }_1\Vert _{H_q^1}$, $\Vert \eta _2\Vert _{H_q^1}$, $\Vert \grave{\eta }_2\Vert _{H_q^1} \le \rho _{\varvec{\mathfrak {N}}}$, then the following hold.

(i) $$\Vert \varvec{\mathfrak {N}}^{\nu }(\varvec{\eta })\Vert _{\mathcal {X}} \le C_{\varvec{\mathfrak {N}}}\big (\nu ^{1/3} + \Vert \varvec{\eta }\Vert _{\mathcal {X}}^2\big )$$.
(ii) $$\Vert \varvec{\mathfrak {N}}^{\nu }(\varvec{\eta })-\varvec{\mathfrak {N}}^{\nu }(\grave{\varvec{\eta }})\Vert _{\mathcal {X}} \le C_{\varvec{\mathfrak {N}}}\big (\nu ^{1/3} + \Vert \varvec{\eta }\Vert _{\mathcal {X}} + \Vert \grave{\varvec{\eta }}\Vert _{\mathcal {X}}\big )\Vert \varvec{\eta }-\grave{\varvec{\eta }}\Vert _{\mathcal {X}}$$.

Assuming this lemma to be true, we define

$$\begin{aligned} C_{\star } := C_{\varvec{\mathfrak {N}}} \qquad \text { and }\qquad \nu _{\star } := \frac{1}{2}\min \left\{ \nu _{\mathcal {M}},1,\frac{1}{(1+C_{\star }^2)^6}, \frac{1}{64C_{\star }^6(1+2C_{\star })^6},\rho _{\varvec{\mathfrak {N}}}\right\} . \end{aligned}$$

Take $0< \nu < \nu _{\star }$ and $\varvec{\eta }$, $\grave{\varvec{\eta }} \in \mathfrak {B}(C_{\star }\nu )$. Then by part (i) of Lemma B.1 we have

$$\begin{aligned} \Vert \varvec{\mathfrak {N}}^{\nu }(\varvec{\eta })\Vert _{\mathcal {X}} \le C_{\varvec{\mathfrak {N}}}\big (\nu ^{1/3} + \Vert \varvec{\eta }\Vert _{\mathcal {X}}^2\big ) \le C_{\star } \big [C_{\star }(1+C_{\star }^2)\nu ^{1/6}\big ]\nu ^{1/3} \le C_{\star }\nu ^{1/3}. \end{aligned}$$

This proves part (i) of Proposition 6.1. Next, part (ii) of that lemma gives

$$\begin{aligned}&\Vert \varvec{\mathfrak {N}}^{\nu }(\varvec{\eta })-\varvec{\mathfrak {N}}^{\nu }(\grave{\varvec{\eta }})\Vert _{\mathcal {X}} \le C_{\varvec{\mathfrak {N}}}\big (\nu ^{1/3}+2C_{\varvec{\mathfrak {N}}}\nu ^{1/6}\big )\Vert \varvec{\eta }-\grave{\varvec{\eta }}\Vert _{\mathcal {X}}\\&\quad \le C_{\star }\big (1+2C_{\star })\nu ^{1/6}\Vert \varvec{\eta }-\grave{\varvec{\eta }}\Vert _{\mathcal {X}} \le \frac{1}{2}\Vert \varvec{\eta }-\grave{\varvec{\eta }}\Vert _{\mathcal {X}}. \end{aligned}$$

This proves part (ii) of Proposition 6.1.

In the remainder of this appendix, we first give some essential auxiliary estimates in Appendix B.1. Then we prove the Lipschitz estimates of part (ii) of Proposition 6.1 in Appendix B.2. Finally, using in part these Lipschitz estimates, we prove in Appendix B.3 the mapping estimates from part (i) of the proposition.

### B.1. Auxiliary estimates

Throughout this appendix we will frequently obtain estimates in terms of the $L^1$- or $L^{\infty }$-norms of a function $f \in H_q^1$. Afterwards we can use the embedding of $L_q^2$ into $L^1$ and the corresponding inequalities

$$\begin{aligned} \Vert f\Vert _{L^1} \le C\Vert f\Vert _{L_q^2} \le C\Vert f\Vert _{H_q^1} \end{aligned}$$

for $f \in H_q^1$, as well as the Sobolev embedding, to turn these $L^1$- and $L^{\infty }$-estimates into $H_q^1$ estimates. For brevity, we will omit those details.

We will also use the operator $\mathcal {A}$ defined in (5.4); we recall

$$\begin{aligned} (\mathcal {A}{f})(X) = \int _X^{\infty } f(W) \ dW\end{aligned}$$

for $f \in H_q^1$, so that

$$\begin{aligned} f = -\partial _X[\mathcal {A}{f}] \qquad \text { and }\qquad \Vert \mathcal {A}{f}\Vert _{L^{\infty }} \le C\Vert f\Vert _{L^1}. \end{aligned}$$

Last, we will use the shift operator $S^{\nu }$, which satisfies

$$\begin{aligned} (S^{\nu }f)(X) = f(X+\nu ) \end{aligned}$$

for any function *f* defined on $\mathbb {R}$ and any $\nu \in \mathbb {R}$.

The operator $\mathcal {A}$ and the definitions of our fundamental operators $\mathcal {P}_1^{\nu }$ in (3.33) and $\mathcal {R}_1^{\nu }$ in (3.35) allow us to rewrite

$$\begin{aligned} \mathcal {P}_1^{\nu }(f)(X) = \frac{\alpha }{c_0}\mathcal {A}\big [\mathcal {E}(\nu ^{1/2}S^{\nu }f)(\cdot ,X)f\big ](X) \end{aligned}$$

and

$$\begin{aligned} \mathcal {R}_1^{\nu }(f)(X) = \frac{\alpha }{c_0^2\tau _1}\int _X^{\infty } \mathcal {A}\big [\mathcal {E}(\nu ^{1/2}S^{\nu }f)(\cdot ,V)f\big ](V)(S^{\nu }f)(V) \ dV. \end{aligned}$$

Now we begin our estimates on $\mathcal {E}$, $\mathcal {P}_1^{\nu }$, and $\mathcal {R}_1^{\nu }$ in earnest.

#### Lemma B.2

There exists $C > 0$ such that if $\Vert f\Vert _{L^1}$, $\Vert \grave{f}\Vert _{L^1} \le 1$, then the following hold.

(i) $$|\mathcal {E}(f)(V,X)-\mathcal {E}(\grave{f})(V,X)| \le C\Vert f-\grave{f}\Vert _{L^1}$$ for all *V*, $$X \in \mathbb {R}$$.
(ii) $|\mathcal {E}(f)(V,X)| \le C$ for all *V*, $X \in \mathbb {R}$.

#### Proof

(i) Since
  $$\begin{aligned} \left| \frac{\kappa }{c_0}\int _V^X f(U) \ dU\right| \le \frac{\kappa }{c_0}\Vert f\Vert _{L^1} \end{aligned}$$
  for all *V*, $X \in \mathbb {R}$, a local Lipschitz estimate on the exponential yields $C > 0$ such that if $\Vert f\Vert _{L^1}$, $\Vert \grave{f}\Vert _{L^1} \le 1$, then
  $$\begin{aligned} |\mathcal {E}(f)(V,X)-\mathcal {E}(\grave{f})(V,X)| \le C\left| \int _V^X f(U) \ dU- \int _V^X \grave{f}(U) \ dU\right| \le C\Vert f-\grave{f}\Vert _{L^1}. \end{aligned}$$
(ii) Since $\mathcal {E}(0) = 0$, this follows from part (i) by taking $\grave{f} = 0$.

$$\square $$

The following lemma guarantees that $\mathcal {P}_1^{\nu }$ maps $H_q^1$ to $W^{1,\infty }$, among other results.

#### Lemma B.3

There exists $C> 0$ such that if $0 \le \nu < 1$ and *f*, $\grave{f} \in H_q^1$ with $\Vert f\Vert _{H_q^1}$, $\Vert \grave{f}\Vert _{H_q^1} \le 1$, then the following hold.

(i) $$\Vert \mathcal {P}_1^{\nu }(f)-\mathcal {P}_1^{\nu }(\grave{f})\Vert _{L^{\infty }} \le C\big (\nu ^{1/2}\Vert f\Vert _{H_q^1}+1\big )\Vert f-\grave{f}\Vert _{H_q^1}$$.
(ii) $$\Vert \mathcal {P}_1^{\nu }(f)\Vert _{L^{\infty }} \le C\Vert f\Vert _{H_q^1}$$.
(iii) $$\Vert \mathcal {P}_1^{\nu }(f)-\mathcal {P}_1^0(f)\Vert _{L^{\infty }} \le C\nu ^{1/2}\Vert f\Vert _{H_q^1}^2$$.
(iv) $$\Vert \partial _X[\mathcal {P}_1^{\nu }(f)-\mathcal {P}_1^0(f)]\Vert _{L^{\infty }} \le C\nu ^{1/2}\Vert f\Vert _{H_q^1}^2$$.
(v) $$\Vert \big (\mathcal {P}_1^{\nu }(f)-\mathcal {P}_1^0(f)\big )-\big (\mathcal {P}_1^{\nu }(\grave{f})-\mathcal {P}_1^0(\grave{f})\big )\Vert _{L^{\infty }} \le C\nu ^{1/2}\big (\Vert f\Vert _{H_q^1}+\Vert \grave{f}\Vert _{H_q^1}\big )\Vert f-\grave{f}\Vert _{H_q^1}$$.
(vi) $$\Vert \partial _X[\mathcal {P}_1^{\nu }(f)-\mathcal {P}_1^0(f)]\big )-\big (\partial _X[\mathcal {P}_1^{\nu }(\grave{f})-\mathcal {P}_1^0(\grave{f})]\Vert _{L^{\infty }} \le C\nu ^{1/2}\big (\Vert f\Vert _{H_q^1}+\Vert \grave{f}\Vert _{H_q^1}\big )\Vert f-\grave{f}\Vert _{H_q^1}$$.

#### Proof

As we mentioned earlier, in most cases we will conclude bounds in terms of $L^{\infty }$- or $L^1$-norms, which then immediately yield the $H_q^1$-bounds stated above.

(i) We have
  $$\begin{aligned} \mathcal {P}_1^{\nu }(f)(X) - \mathcal {P}_1^{\nu }(\grave{f})(X) = \mathcal {I}_1^{\nu }(f,\grave{f})(X) + \mathcal {I}_2^{\nu }(f,\grave{f})(X), \end{aligned}$$
  where
  $$\begin{aligned} \mathcal {I}_1^{\nu }(f,\grave{f})(X) := \frac{\alpha }{c_0}\int _X^{\infty } \big (\mathcal {E}(\nu ^{1/2}S^{\nu }f)(V,X)-\mathcal {E}(\nu ^{1/2}S^{\nu }\grave{f})(V,X)\big )f(V) \ dV\end{aligned}$$
  and
  $$\begin{aligned} \mathcal {I}_2^{\nu }(f,\grave{f})(X) := \frac{\alpha }{c_0}\int _X^{\infty } \mathcal {E}(\nu ^{1/2}S^{\nu }\grave{f})(V,X)\big (f(V)-\grave{f}(V)\big ) \ dV. \end{aligned}$$
  We use part (i) of Lemma B.2 to bound
  B.1 $$\begin{aligned}&\big |\mathcal {E}(\nu ^{1/2}S^{\nu }f)(V,X)-\mathcal {E}(\nu ^{1/2}S^{\nu }\grave{f})(V,X)\big | \le C\nu ^{1/2}\Vert S^{\nu }f-S^{\nu }\grave{f}\Vert _{L^1} \nonumber \\&\quad = C\nu ^{1/2}\Vert f-\grave{f}\Vert _{L^1} \end{aligned}$$
  for all *V*, $X \in \mathbb {R}$. Thus
  $$\begin{aligned} |\mathcal {I}_1^{\nu }(f,\grave{f})(X)| \le C\nu ^{1/2}\Vert f-\grave{f}\Vert _{L^1}\int _X^{\infty }|f(V)| \ dV\le C\nu ^{1/2}\Vert f-\grave{f}\Vert _{L^1}\Vert f\Vert _{L^1} \end{aligned}$$
  for all $$X \in \mathbb {R}$$.
  Next, we use part (ii) of Lemma B.2 to bound
  $$\begin{aligned} |\mathcal {I}_2^{\nu }(f,\grave{f})(X)| \le C\int _X^{\infty } |f(V)-\grave{f}(V)| \ dV\le C\Vert f-\grave{f}\Vert _{L^1}. \end{aligned}$$
(ii) Since $\mathcal {P}^{\nu }(0) = 0$, this follows from part (i) by taking $\grave{f} = 0$.
(iii) We have
  B.2 $$\begin{aligned} \mathcal {P}_1^{\nu }(f)(X) - \mathcal {P}_1^0(f)(X) = \frac{\alpha }{c_0}\int _X^{\infty } \big (\mathcal {E}(\nu ^{1/2}S^{\nu }f)(V,X)-1\big )f(V) \ dV.\nonumber \\ \end{aligned}$$
  Since
  $$\begin{aligned} \mathcal {E}(\nu ^{1/2}S^{\nu }f)(V,X)-1 = \mathcal {E}(\nu ^{1/2}S^{\nu }f)(V,X) - \mathcal {E}(0)(V,X), \end{aligned}$$
  we may use part (i) of Lemma B.2 to bound
  B.3 $$\begin{aligned} |\mathcal {E}(\nu ^{1/2}S^{\nu }f)(V,X)-1| \le C\nu ^{1/2}\Vert S^{\nu }f\Vert _{L^1} = C\nu ^{1/2}\Vert f\Vert _{L^1}. \end{aligned}$$
  Thus
  $$\begin{aligned} |\mathcal {P}_1^{\nu }(f)(X) - \mathcal {P}_1^0(f)(X)| \le C\nu ^{1/2}\Vert f\Vert _{L^1}\int _X^{\infty } |f(V)| \ dV\le C\nu ^{1/2}\Vert f\Vert _{L^1}^2. \end{aligned}$$
(iv) We first differentiate under the integral and use the condition $\mathcal {E}(g)(X,X) = 1$, apparent from the definition of $\mathcal {E}$ in (3.3) and valid for all integrable *g* and $X \in \mathbb {R}$, to calculate
  $$\begin{aligned} \partial _X[\mathcal {P}_1^{\nu }(f)](X)= & {} -\frac{\alpha }{c_0}f(X) + \left( \frac{\alpha }{c_0}\right) ^2\nu ^{1/2}\\&\int _X^{\infty } \mathcal {E}(\nu ^{1/2}S^{\nu }f)(V,X)f(V+\nu )f(V) \ dV. \end{aligned}$$
  Then
  B.4 $$\begin{aligned} \partial _X[\mathcal {P}_1^{\nu }(f)-\mathcal {P}_1^0(f)](X)= & {} \left( \frac{\alpha }{c_0}\right) ^2\nu ^{1/2}\nonumber \\&\int _X^{\infty } \mathcal {E}(\nu ^{1/2}S^{\nu }f)(V,X)f(V+\nu )f(V) \ dV. \qquad \qquad \end{aligned}$$
  Part (ii) of Lemma B.2 then guarantees
  $$\begin{aligned} \Vert \partial _X[\mathcal {P}_1^{\nu }(f)-\mathcal {P}_1^0(f)]\Vert _{L^{\infty }} \le C\nu ^{1/2}\Vert f\Vert _{L^{\infty }}\Vert f\Vert _{L^1}. \end{aligned}$$
(v) We use (B.2) to write
  $$\begin{aligned} \big (\mathcal {P}_1^{\nu }(f)-\mathcal {P}_1^0(f)\big )-\big (\mathcal {P}_1^{\nu }(\grave{f})-\mathcal {P}_1^0(\grave{f})\big ) = \mathcal {I}_3^{\nu }(f,\grave{f}) + \mathcal {I}_4^{\nu }(f,\grave{f}), \end{aligned}$$
  where
  $$\begin{aligned} \mathcal {I}_3^{\nu }(f,\grave{f})(X) := \frac{\alpha }{c_0}\int _X^{\infty } \big (\mathcal {E}(\nu ^{1/2}S^{\nu }f)(V,X)-\mathcal {E}(\nu ^{1/2}S^{\nu }\grave{f})(V,X)\big )f(V) \ dV\end{aligned}$$
  and
  $$\begin{aligned} \mathcal {I}_4^{\nu }(f,\grave{f})(X) := \frac{\alpha }{c_0}\int _X^{\infty } \big (\mathcal {E}(\nu ^{1/2}S^{\nu }\grave{f})(V,X)-1\big )\big (f(V)-\grave{f}(V)\big ) \ dV. \end{aligned}$$
  We use (B.1) to estimate
  $$\begin{aligned} |\mathcal {I}_3^{\nu }(f,\grave{f})(X)| \le C\nu ^{1/2}\Vert f-\grave{f}\Vert _{L^1}\int _X^{\infty } |f(V)| \ dV\le C\nu ^{1/2}\Vert f\Vert _{L^1}\Vert f-\grave{f}\Vert _{L^1}. \end{aligned}$$
  We use (B.3) to estimate
  $$\begin{aligned} |\mathcal {I}_3^{\nu }(f,\grave{f})(X)|&\le C\nu ^{1/2}\Vert \grave{f}\Vert _{L^1}\int _X^{\infty } |f(V)-\grave{f}(V)| \ dV\\&\le C\nu ^{1/2}\Vert \grave{f}\Vert _{L^1}\Vert f-\grave{f}\Vert _{L^1}. \end{aligned}$$
(vi) Using (B.4), we have
  $$\begin{aligned}&\partial _X[\mathcal {P}_1^{\nu }(f)-\mathcal {P}_1^0(f)](X)-\partial _X[\mathcal {P}_1^{\nu }(\grave{f})-\mathcal {P}_1^0(\grave{f})](X) \\&\quad = \left( \frac{\alpha }{c_0}\right) ^2\nu ^{1/2}\int _X^{\infty } \big [\mathcal {E}(\nu ^{1/2}S^{\nu }f)(V,X)f(V+\nu )f(V)\\&\qquad -\mathcal {E}(\nu ^{1/2}S^{\nu }\grave{f})(V,X)\grave{f}(V+\nu )\grave{f}(V)\big ] \ dV. \end{aligned}$$
  The estimate follows in a manner analogous to the proof of part (v) above, so we omit the details.

$$\square $$

The next lemma guarantees that $\mathcal {R}_1^{\nu }$ maps $H_q^1$ to $W^{1,\infty }$.

#### Lemma B.4

There exists $C> 0$ such that if $0 \le \nu < 1$ and $\Vert f\Vert _{H_q^1}$, $\Vert \grave{f}\Vert _{H_q^1} \le 1$, then the following hold.

(i) $$\Vert \mathcal {R}^{\nu }(f)\Vert _{L^{\infty }} \le C\Vert f\Vert _{H_q^1}^2$$.
(ii) $$\Vert \partial _X[\mathcal {R}^{\nu }(f)]\Vert _{L^{\infty }} \le C\Vert f\Vert _{H_q^1}^2$$.
(iii) $$\Vert \mathcal {R}^{\nu }(f) - \mathcal {R}^{\nu }(\grave{f})\Vert _{L^{\infty }} \le C\big (\nu ^{1/2}+\Vert f\Vert _{H_q^1} + \Vert \grave{f}\Vert _{H_q^1}\big )\Vert f-\grave{f}\Vert _{H_q^1}$$.
(iv) $$\Vert \partial _X[\mathcal {R}^{\nu }(f) - \mathcal {R}^{\nu }(\grave{f})]\Vert _{L^{\infty }} \le C\big (\nu ^{1/2}+\Vert f\Vert _{H_q^1} + \Vert \grave{f}\Vert _{H_q^1}\big )\Vert f-\grave{f}\Vert _{H_q^1}$$
(v) $$\Vert \mathcal {R}^{\nu }(f) - \mathcal {R}_1^0(f)\Vert _{L^{\infty }} \le C\nu ^{1/2}\Vert f\Vert _{H_q^1}^2$$.
(vi) $$\Vert \big (\mathcal {R}^{\nu }(f)-\mathcal {R}_1^0(f)\big )-\big (\mathcal {R}^{\nu }(\grave{f})-\mathcal {R}_1^0(\grave{f})\big )\Vert _{L^{\infty }} \le C\big (\nu ^{1/2} + \Vert f\Vert _{H_q^1} + \Vert \grave{f}\Vert _{H_q^1}\big )\Vert f-\grave{f}\Vert _{H_q^1}$$.
(vii) $$\Vert \mathcal {R}^{\nu }(f)-\mathcal {R}_1^0(f)\Vert _{L^{\infty }} \le C\nu ^{1/2}\Vert f\Vert _{H_q^1}^2$$.

#### Proof

Throughout we will use the inequality

$$\begin{aligned} \Vert \mathcal {R}^{\nu }(f)\Vert _{L^{\infty }} \le \Vert \mathcal {P}_1^{\nu }(f)(S^{\nu }f)\Vert _{L^1}. \end{aligned}$$

As before, we stop when we have bounds in terms of $L^1$- or $L^{\infty }$-norms.

(i) We use part (ii) of Lemma B.3 to bound
  $$\begin{aligned} \Vert \mathcal {R}^{\nu }(f)\Vert _{L^{\infty }} = C\Vert \mathcal {P}_1^{\nu }(f)(S^{\nu }f)\Vert _{L^1} \le C\Vert \mathcal {P}_1^{\nu }(f)\Vert _{L^{\infty }}\Vert S^{\nu }f\Vert _{L^1} \le C\Vert f\Vert _{L^1}^2. \end{aligned}$$
(ii) We have
  $$\begin{aligned} \partial _X[\mathcal {R}^{\nu }(f)] = -\frac{\alpha }{c_0^2\tau _1}\mathcal {P}_1^{\nu }(f)(S^{\nu }f), \end{aligned}$$
  thus
  $$\begin{aligned} \Vert \partial _X[\mathcal {R}^{\nu }(f)]\Vert _{L^{\infty }} \le C\Vert \mathcal {P}_1^{\nu }(f)(S^{\nu }f)\Vert _{L^{\infty }} \le C\Vert \mathcal {P}_1^{\nu }(f)\Vert _{L^{\infty }}\Vert f\Vert _{H_q^1} \le C\Vert f\Vert _{H_q^1}^2 \end{aligned}$$
  by the Sobolev embedding and part (ii) of Lemma B.3.
(iii) We use parts (i) and (ii) of Lemma B.3 to bound
  $$\begin{aligned}&\Vert \mathcal {R}^{\nu }(f)-\mathcal {R}^{\nu }(\grave{f})\Vert _{L^{\infty }} \le C\Vert \big (\mathcal {P}_1^{\nu }(f)-\mathcal {P}_1^{\nu }(f)\big )f\Vert _{L^1} + C\Vert \mathcal {P}_1^{\nu }(\grave{f})\big (S^{\nu }(f-\grave{f})\big )\Vert _{L^1} \\&\quad \le C\Vert \mathcal {P}_1^{\nu }(f)-\mathcal {P}_1^{\nu }(\grave{f})\Vert _{L^{\infty }}\Vert f\Vert _{L^1} + C\Vert \mathcal {P}_1^{\nu }(\grave{f})\Vert _{L^{\infty }}\Vert S^{\nu }(f-\grave{f})\Vert _{L^1} \\&\quad \le C\big (\nu ^{1/2}\Vert f\Vert _{L^1} + 1\big )\Vert f\Vert _{L^1}\Vert f-\grave{f}\Vert _{L^1} + C\Vert \grave{f}\Vert _{L^1}\Vert f-\grave{f}\Vert _{L^1}. \end{aligned}$$
(iv) We have
  $$\begin{aligned} \partial _X[\mathcal {R}^{\nu }(f)-\mathcal {R}^{\nu }(\grave{f})] = \frac{\alpha }{c_0^2\tau _1}\mathcal {P}_1^{\nu }(\grave{f})(S^{\nu }\grave{f}) - \frac{\alpha }{c_0^2\tau _1}\mathcal {P}_1^{\nu }(f)(S^{\nu }f), \end{aligned}$$
  thus
  $$\begin{aligned}&\Vert \partial _X[\mathcal {R}^{\nu }(f)-\mathcal {R}^{\nu }(\grave{f})]\Vert _{L^{\infty }} \le C\Vert \big (\mathcal {P}_1^{\nu }(f)-\mathcal {P}_1^{\nu }(\grave{f})\big )\grave{f}\Vert _{L^{\infty }}\\&\quad + C\Vert \mathcal {P}_1^{\nu }(\grave{f})\big (S^{\nu }(f-\grave{f})\big )\Vert _{L^{\infty }}. \end{aligned}$$
  We use part (i) of Lemma B.3 and the Sobolev embedding to estimate
  $$\begin{aligned}&\Vert \big (\mathcal {P}_1^{\nu }(f)-\mathcal {P}_1^{\nu }(\grave{f})\big )\grave{f}\Vert _{L^{\infty }} \le \Vert \mathcal {P}_1^{\nu }(f)-\mathcal {P}_1^{\nu }(\grave{f})\Vert _{L^{\infty }}\Vert f\Vert _{H_q^1}\\&\quad \le C\big (\nu ^{1/2}\Vert f\Vert _{L^1}+1\big )\Vert f\Vert _{H_q^1}\Vert f-\grave{f}\Vert _{L^1} \end{aligned}$$
  and part (ii) of Lemma B.3 and the Sobolev embedding to estimate
  $$\begin{aligned} \Vert \mathcal {P}_1^{\nu }(\grave{f})\big (S^{\nu }(f-\grave{f})\big )\Vert _{L^{\infty }} \le C\Vert \mathcal {P}_1^{\nu }(\grave{f})\Vert _{L^{\infty }}\Vert f-\grave{f}\Vert _{L^{\infty }} \le C\Vert f\Vert _{L^1}\Vert f-\grave{f}\Vert _{H_q^1}. \end{aligned}$$
(v) We first estimate
  $$\begin{aligned}&\Vert \mathcal {R}^{\nu }(f)-\mathcal {R}_1^0(f)\Vert _{L^{\infty }} \le C\Vert \mathcal {P}_1^{\nu }(f)(S^{\nu }f) - \mathcal {P}_1^0(f)f\Vert _{L^1}\\&\qquad \le C\Vert \big (\mathcal {P}_1^{\nu }(f)-\mathcal {P}_1^0(f)\big )(S^{\nu }f)\Vert _{L^1} \\&\qquad \quad + C\Vert \mathcal {P}_1^0(f)(S^{\nu }f-f)\Vert _{L^1}. \end{aligned}$$
  Then part (iii) of Lemma B.3 gives
  $$\begin{aligned} \Vert \big (\mathcal {P}_1^{\nu }(f)-\mathcal {P}_1^0(f)\big )(S^{\nu }f)\Vert _{L^1} \le \Vert \mathcal {P}_1^{\nu }(f)-\mathcal {P}_1^0(f)\Vert _{L^{\infty }}\Vert S^{\nu }f\Vert _{L^1} \le C\nu ^{1/2}\Vert f\Vert _{L^1}^3. \end{aligned}$$
  Next, part (ii) of Lemma B.3 implies
  $$\begin{aligned} \Vert \mathcal {P}_1^0(f)(S^{\nu }f-f)\Vert _{L^1} \le \Vert \mathcal {P}_1^0(f)\Vert _{L^{\infty }}\Vert S^{\nu }f-f\Vert _{L^1} \le C\Vert f\Vert _{L^1}\Vert (S^{\nu }-1)f\Vert _{L^1}. \end{aligned}$$
  Since $f \in H_q^1$, we have
  $$\begin{aligned} \Vert (S^{\nu }-1)f\Vert _{L^1} \le C_q\Vert (S^{\nu }-1)f\Vert _{L_q^2}. \end{aligned}$$
  It follows from Faver and Wright (2018, Lem. A.11) that
  $$\begin{aligned} \Vert (S^{\nu }-1)f\Vert _{L_q^2} \le C\nu \Vert f\Vert _{H_q^1}. \end{aligned}$$
(vi) We estimate
  $$\begin{aligned}&\Vert \big (\mathcal {R}^{\nu }(f)-\mathcal {R}_1^0(f)\big )-\big (\mathcal {R}^{\nu }(\grave{f})-\mathcal {R}_1^0(\grave{f})\big )\Vert _{L^{\infty }} \le C\Vert \big (\mathcal {P}_1^{\nu }(f)(S^{\nu }f) \\&\qquad - \mathcal {P}_1^0(f)f\big ) - \big (\mathcal {P}^{\nu }(\grave{f})(S^{\nu }\grave{f}) - \mathcal {P}_1^0(\grave{f})\grave{f}\big )\Vert _{L^1}\\&\quad \le C\Vert \big (\mathcal {P}_1^{\nu }(f)-\mathcal {P}_1^{\nu }(\grave{f})\big )(S^{\nu }f)\Vert _{L^1} + C\Vert \mathcal {P}_1^{\nu }(\grave{f})\big (S^{\nu }(f-\grave{f})\big )\Vert _{L^1}\\&\qquad + C\Vert \big (P_1^0(f)-\mathcal {P}_1^0(\grave{f})\big )\grave{f}\Vert _{L^1} + C\Vert \mathcal {P}_1^0(f)(f-\grave{f})\Vert _{L^1}. \end{aligned}$$
  We use part (ii) of Lemma B.3 to bound
  $$\begin{aligned}&\Vert \mathcal {P}_1^{\nu }(\grave{f})\big (S^{\nu }(f-\grave{f})\big )\Vert _{L^1} + \Vert \mathcal {P}_1^0(f)(f-\grave{f})\Vert _{L^1} \le \Vert \mathcal {P}_1^{\nu }(\grave{f}\Vert _{L^{\infty }}\Vert S^{\nu }(f-\grave{f})\Vert _{L^1}\\&\qquad + \Vert \mathcal {P}_1^0(f)\Vert _{L^{\infty }}\Vert f-\grave{f}\Vert _{L^1} \\&\quad \le C\Vert f\Vert _{L^1}\Vert f-\grave{f}\Vert _{L^1}. \end{aligned}$$
  We use part (i) of Lemma B.3 to bound
  $$\begin{aligned}&\Vert \big (\mathcal {P}_1^{\nu }(f)-\mathcal {P}_1^{\nu }(\grave{f})\big )(S^{\nu }f)\Vert _{L^1} + \Vert \big (P_1^0(f)-\mathcal {P}_1^0(\grave{f})\big )\grave{f}\Vert _{L^1} \\&\quad \le \Vert \mathcal {P}_1^{\nu }(f)-\mathcal {P}_1^{\nu }(\grave{f})\Vert _{L^{\infty }}\Vert S^{\nu }f\Vert _{L^1} + \Vert \mathcal {P}_1^0(f)-\mathcal {P}_1^0(\grave{f})\Vert _{L^{\infty }}\Vert \grave{f}\Vert _{L^1} \\&\quad \le C\big (\nu ^{1/2}\Vert f\Vert _{L^1}+1\big )\Vert f\Vert _{L^1}\Vert f-\grave{f}\Vert _{L^1} + C\Vert \grave{f}\Vert _{L^1}\Vert f-\grave{f}\Vert _{L^1}. \end{aligned}$$
(vii) We use part (vi) with $\grave{f} = 0$.

$$\square $$

Finally, we present estimates on the operators $\mathcal {N}^{\nu }$ defined in (3.38) and $\mathcal {P}_2^{\nu }$ from (3.34).

#### Lemma B.5

There exist *C*, $\rho _0 > 0$ such that if $0 \le \nu < 1$, then the following hold.

(i) If *f*, $\grave{f} \in H_q^1$ and *g*, $\grave{g} \in W^{1,\infty }$ with $\Vert f\Vert _{H_q^1} + \Vert g\Vert _{W^{1,\infty }} \le \rho _0$ and $\Vert \grave{f}\Vert _{H_q^1} + \Vert \grave{g}\Vert _{W^{1,\infty }} \le \rho _0$, then
  $$\begin{aligned}&\Vert \mathcal {N}^{\nu }(f,g) - \mathcal {N}^{\nu }(\grave{f},\grave{g})\Vert _{L_q^2} + \Vert \mathcal {P}_2^{\nu }(f,g) - \mathcal {P}_2^{\nu }(\grave{f},\grave{g})\Vert _{H_q^1} \\&\quad \le C\big (\nu ^{1/2} + \Vert f\Vert _{H_q^1} + \Vert \grave{f}\Vert _{H_q^1} + \Vert g\Vert _{W^{1,\infty }} + \Vert \grave{g}\Vert _{W^{1,\infty }}\big )\\&\qquad \big (\Vert f-\grave{f}\Vert _{H_q^1} + \Vert g-\grave{g}\Vert _{W^{1,\infty }}\big ). \end{aligned}$$
(ii) If $f \in H_q^1$ and $g \in W^{1,\infty }$ with $\Vert f\Vert _{H_q^1} + \Vert g\Vert _{W^{1,\infty }} \le \rho _0$, then
  $$\begin{aligned} \Vert \mathcal {N}^{\nu }(f,g)\Vert _{L_q^2} + \Vert \mathcal {P}_2^{\nu }(f,g)\Vert _{W^{1,\infty }} \le C. \end{aligned}$$

#### Proof

Part (ii) follows from part (i) since $\mathcal {N}^{\nu }(0,0) = \mathcal {P}_2^{\nu }(0,0) = 0$. The proof of the Lipschitz estimates in part (i) follows exactly the strategies deployed above, and we would learn almost nothing new from seeing its argument, so we omit that. The one difference here is that $\mathcal {N}^{\nu }$ and $\mathcal {P}_2^{\nu }$ incorporate the maps $\mathcal {Q}_1^{\nu }$ and $\mathcal {Q}_2^{\nu }$, which were defined in (3.32) and which are really rational functions from $\mathbb {R}^2$ to $\mathbb {R}$. A glance at the formulas for $\mathcal {Q}_1^{\nu }$ and $\mathcal {Q}_2^{\nu }$ provides $\rho _{\mathcal {Q}} > 0$ such that if $0< \nu < 1$, then $\mathcal {Q}_1^{\nu }$ and $\mathcal {Q}_2^{\nu }$ are defined and smooth on the ball $\left\{ (X,Y) \in \mathbb {R}^2 \ | \ |X| + |Y| \le \rho _{\mathcal {Q}}\right\}$. By taking $\Vert f\Vert _{H_q^1} + \Vert g\Vert _{W^{1,\infty }} \le \rho _0$ for some small $\rho _0 > 0$, we can guarantee that the compositions involving $\mathcal {Q}_1^{\nu }$ and $\mathcal {Q}_2^{\nu }$ with *f*, *g*, and other operators acting on *f* and *g* are all defined and satisfy tame Lipschitz estimates. $\square$

### B.2. Lipschitz estimates

We first prove the Lipschitz estimates undergirding part (ii) of Lemma B.1, which we then use to prove the mapping estimates in part (i). From (6.7), we have $\varvec{\mathfrak {N}}^{\nu } = (\mathfrak {N}_1^{\nu },\mathfrak {N}_2^{\nu })$, where $\mathfrak {N}_1^{\nu }$ was defined in (6.5) and $\mathfrak {N}_2^{\nu }$ in (6.6). Using these definitions and the boundedness of the operator $\mathcal {T}^{-1}$ from Proposition 5.1, we can prove part (ii) of Lemma B.1 if we show

$$\begin{aligned}&\sum _{k=1}^5 \big (\Vert \mathcal {V}_{1k}^{\nu }(\varvec{\eta })-\mathcal {V}_{1k}^{\nu }(\grave{\varvec{\eta }})\Vert _{H_q^1}\big ) + \big (\Vert \mathcal {V}_{21}^{\nu }(\varvec{\eta })-\mathcal {V}_{21}^{\nu }(\grave{\varvec{\eta }})\Vert _{W^{1,\infty }}\big ) \\&\quad + \Vert \mathcal {V}_{23}^{\nu }(\varvec{\eta })-\mathcal {V}_{23}^{\nu }(\grave{\varvec{\eta }})\Vert _{W^{1,\infty }}\big ) \le C\mathfrak {R}_{\star }^{\nu }(\varvec{\eta },\grave{\varvec{\eta }}), \end{aligned}$$

where

$$\begin{aligned} \mathfrak {R}_{\star }^{\nu }(\varvec{\eta },\grave{\varvec{\eta }}):= & {} \big (\nu ^{1/3} + \Vert \eta _1\Vert _{H_q^1} + \Vert \grave{\eta }_1\Vert _{H_q^1} + \Vert \eta _2\Vert _{W^{1,\infty }} + \Vert \grave{\eta }_2\Vert _{W^{1,\infty }}\big )\\&\big (\Vert \eta _1-\grave{\eta _1}\Vert _{H_q^1}+ \Vert \eta _2-\grave{\eta }_2\Vert _{W^{1,\infty }}\big ). \end{aligned}$$

The terms $\mathcal {V}_{1k}^{\nu }$ were defined in (6.3) and $\mathcal {V}_{2k}^{\nu }$ in (6.4).

#### B.2.1. Lipschitz estimates on $\mathcal {V}_{11}^{\nu }$

We use the estimate on $\mathcal {M}^{(\nu )}-\mathcal {M}^{(0)}$ from Proposition 4.1 to obtain

$$\begin{aligned} \Vert \mathcal {V}_{11}^{\nu }(\varvec{\eta })-\mathcal {V}_{11}^{\nu }(\grave{\varvec{\eta }})\Vert _{H_q^1}\le & {} C\nu ^{1/3}\Vert \big (\mathcal {R}^{\nu }(\sigma +\eta _1)-\mathcal {R}^{\nu }(\sigma +\grave{\eta }_1)\big )(\sigma +\grave{\eta }_1)\Vert _{H_q^1}\\&+ C\nu ^{1/3}\Vert \mathcal {R}^{\nu }(\sigma +\eta _1)(\eta _1-\grave{\eta }_1)\Vert _{H_q^1}. \end{aligned}$$

We first estimate

$$\begin{aligned}&\Vert \big (\mathcal {R}^{\nu }(\sigma +\eta _1)-\mathcal {R}^{\nu }(\sigma +\grave{\eta }_1)\big )(\sigma +\grave{\eta }_1)\Vert _{H_q^1} \le \Vert \partial _X\big [\mathcal {R}^{\nu }(\sigma +\eta _1)-\mathcal {R}^{\nu }(\sigma +\grave{\eta }_1)\big ]\\&\qquad (\sigma +\grave{\eta }_1)\Vert _{L_q^2}+ \Vert \big (\mathcal {R}^{\nu }(\sigma +\eta _1)-\mathcal {R}^{\nu }(\sigma +\grave{\eta }_1)\big )\partial _X[\sigma +\grave{\eta }_1]\Vert _{L_q^2}, \end{aligned}$$

where

$$\begin{aligned}&\Vert \partial _X\big [\mathcal {R}^{\nu }(\sigma +\eta _1)-\mathcal {R}^{\nu }(\sigma +\grave{\eta }_1)\big ](\sigma +\grave{\eta }_1)\Vert _{L_q^2} \\&\qquad \le \Vert \partial _X\big [\mathcal {R}^{\nu }(\sigma +\eta _1)-\mathcal {R}_1^{\nu }(\sigma +\grave{\eta }_1)\big ]\Vert _{L^{\infty }} \Vert \sigma +\grave{\eta }_1\Vert _{L_q^2}\\&\qquad \le C\big (\nu ^{1/2}+\Vert \eta _1\Vert _{H_q^1}+\Vert \grave{\eta }_1\Vert _{H_q^1}\big )\Vert \eta _1-\grave{\eta }_1\Vert _{H_q^1} \end{aligned}$$

by part (iv) of Lemma B.4 and

$$\begin{aligned}&\Vert \big (\mathcal {R}_1^{\nu }(\sigma +\eta _1)-\mathcal {R}_1^{\nu }(\sigma +\grave{\eta }_1)\big )\partial _X[\sigma +\grave{\eta }_1]\Vert _{L_q^2}, \\&\qquad \le \Vert \mathcal {R}_1^{\nu }(\sigma +\eta _1)-\mathcal {R}_1^{\nu }(\sigma +\grave{\eta }_1)\Vert _{L^{\infty }}\Vert \partial _X[\sigma +\grave{\eta }_1]\Vert _{L_q^2}\\&\qquad \le C\big (\nu ^{1/2}+\Vert \eta _1\Vert _{H_q^1}+\Vert \grave{\eta }_1\Vert _{H_q^1}\big )\Vert \eta _1-\grave{\eta }_1\Vert _{H_q^1} \end{aligned}$$

by part (iii) of Lemma B.4.

Next we estimate

$$\begin{aligned}&\Vert \mathcal {R}_1^{\nu }(\sigma +\eta _1)(\eta _1-\grave{\eta }_1)\Vert _{H_q^1} \le \Vert \partial _X\big [\mathcal {R}_1^{\nu }(\sigma +\eta _1)\big ](\eta _1-\grave{\eta }_1)\Vert _{L_q^2}\\&\quad + \Vert \mathcal {R}_1^{\nu }(\sigma +\eta _1)\partial _X[\eta _1-\grave{\eta }_1]\Vert _{L_q^2}, \end{aligned}$$

where

$$\begin{aligned}&\Vert \partial _X\big [\mathcal {R}_1^{\nu }(\sigma +\eta _1)\big ](\eta _1-\grave{\eta }_1)\Vert _{L_q^2} \le \Vert \partial _X\big [\mathcal {R}_1^{\nu }(\sigma +\eta _1)\big ]\Vert _{L^{\infty }}\Vert \eta _1-\grave{\eta }_1\Vert _{L_q^2}\\&\qquad \le C\Vert \sigma +\eta _1\Vert _{H_q^1}^2\Vert \eta _1-\grave{\eta }_1\Vert _{H_q^1} \le C\Vert \eta _1-\grave{\eta }_1\Vert _{H_q^1} \end{aligned}$$

by part (ii) of Lemma B.4 and

$$\begin{aligned}&\Vert \mathcal {R}_1^{\nu }(\sigma +\eta _1)\partial _X[\eta _1-\grave{\eta }_1]\Vert _{L_q^2} \le \Vert \mathcal {R}_1^{\nu }(\sigma +\eta _1)\Vert _{L^{\infty }}\Vert \partial _X[\eta _1-\grave{\eta }_1]\Vert _{L_q^2}\\&\qquad \le C\Vert \sigma +\eta _1\Vert _{H_q^1}^2\Vert \eta _1-\grave{\eta }_1\Vert _{H_q^1} \le C\Vert \eta _1-\grave{\eta }_1\Vert _{H_q^1}^2. \end{aligned}$$

by part (i) of Lemma B.4.

#### B.2.2. Lipschitz estimates on $\mathcal {V}_{12}^{\nu }$

We use the smoothing property of $\mathcal {M}^{(0)}$ from Lemma 3.1 to bound

$$\begin{aligned}&\Vert \mathcal {V}_{12}^{\nu }(\varvec{\eta })-\mathcal {V}_{12}^{\nu }(\grave{\varvec{\eta }})\Vert _{H_q^1} \le C\Vert \big [\big (\mathcal {R}_1^{\nu }(\eta _1)\\&\qquad -\mathcal {R}_1^0(\eta _1)\big )-\big (\mathcal {R}_1^{\nu }(\grave{\eta }_1)-\mathcal {R}_1^0(\grave{\eta }_1)\big )\big ](\sigma +\eta _1)\Vert _{L_q^2} \\&\qquad + C\Vert \big (\mathcal {R}_1^{\nu }(\grave{\eta }_1)-\mathcal {R}_1^0(\grave{\eta }_1)\big )(\eta _1-\grave{\eta }_1)\Vert _{L_q^2}. \end{aligned}$$

Call the two $L_q^2$-norm terms above *I* and $I\!I$. We estimate

$$\begin{aligned} I\le & {} \Vert \big (\mathcal {R}_1^{\nu }(\eta _1)-\mathcal {R}_1^0(\eta _1)\big )-\big (\mathcal {R}_1^{\nu }(\grave{\eta }_1)-\mathcal {R}_1^0(\grave{\eta }_1)\big )\Vert _{L^{\infty }}\Vert \sigma +\eta _1\Vert _{L_q^2} \\\le & {} C\big (\nu ^{1/2} + \Vert \eta _1\Vert _{H_q^1} + \Vert \grave{\eta }_1\Vert _{H_q^1}\big )\Vert \eta _1-\grave{\eta }_1\Vert _{H_q^1}. \end{aligned}$$

by part (vi) of Lemma B.4 and

$$\begin{aligned} I\!I\le \Vert \mathcal {R}_1^{\nu }(\grave{\eta }_1)-\mathcal {R}_1^0(\grave{\eta }_1)\Vert _{L^{\infty }}\Vert \eta _1-\grave{\eta }_1\Vert _{L_q^2} \le C\nu ^{1/2}\Vert \grave{\eta }_1\Vert _{H_q^1}\Vert \eta _1-\grave{\eta }_1\Vert _{H_q^1} \end{aligned}$$

by part (vii) of Lemma B.4.

#### B.2.3. Lipschitz estimates on $\mathcal {V}_{13}^{\nu }$

We use again the smoothing property of $\mathcal {M}^{(0)}$ to bound

$$\begin{aligned}&\Vert \mathcal {V}_{13}^{\nu }(\varvec{\eta })-\mathcal {V}_{13}^{\nu }(\grave{\varvec{\eta }})\Vert _{H_q^1} \le C\Vert \big (\mathcal {R}_1^0(\sigma +\eta _1)-\mathcal {R}_1^0(\sigma )-D\mathcal {R}_1^0(\sigma )\eta _1\big )\sigma \Vert _{L_q^2} \\&\qquad \le C\Vert \mathcal {R}_1^0(\sigma +\eta _1)-\mathcal {R}_1^0(\sigma )-D\mathcal {R}_1^0(\sigma )\eta _1\Vert _{L^{\infty }}\Vert \sigma \Vert _{L_q^2} . \end{aligned}$$

Next we will use the following ‘difference of squares’ estimate, which is proved using the fundamental theorem of calculus. We thank J. Douglas Wright for pointing out this lemma to us.

##### Lemma B.6

Let $\mathcal {X}$ and $\mathcal {Y}$ be Banach spaces with $\mathcal {Z}\subseteq \mathcal {X}$ open and convex and with $0 \in \mathcal {Z}$. Let $f \in \mathcal {C}^1(\mathcal {Z},\mathcal {Y})$ with $Df(0) = 0$, and suppose

$$\begin{aligned} {{\,\mathrm{Lip}\,}}_{\mathcal {Z}}(Df) := \sup _{\begin{array}{c} x,\grave{x} \in \mathcal {Z}\\ x \ne \grave{x} \end{array}} \frac{\Vert Df(x)-Df(\grave{x})\Vert _{\mathbf {B}(\mathcal {X},\mathcal {Y})}}{\Vert x-\grave{x}\Vert _{\mathcal {X}}} < \infty . \end{aligned}$$

Then

$$\begin{aligned} \Vert f(x)-f(\grave{x})\Vert _{\mathcal {Y}} \le \frac{1}{2}{{\,\mathrm{Lip}\,}}_{\mathcal {Z}}(Df)\big (\Vert x\Vert _{\mathcal {X}} + \Vert \grave{x}\Vert _{\mathcal {X}}\big )\Vert x-\grave{x}\Vert _{\mathcal {X}}. \end{aligned}$$

We apply this lemma to $f(\eta _1) := \mathcal {R}_1^0(\sigma )\eta _1)-\mathcal {R}_1^0(\sigma )-D\mathcal {R}_1^0(\sigma )\eta _1$, which is infinitely differentiable as a map from $H_q^1$ to $W^{1,\infty }$ by Remark 3.4, to conclude

$$\begin{aligned} \Vert \mathcal {R}_1^0(\sigma +\eta _1)-\mathcal {R}_1^0(\sigma )-D\mathcal {R}_1^0(\sigma )\eta _1\Vert _{L^{\infty }} \le C\big (\Vert \eta _1\Vert _{H_q^1} + \Vert \grave{\eta }_1\Vert _{H_q^1}\big )\Vert \eta _1-\grave{\eta }_1\Vert _{H_q^1}. \end{aligned}$$

#### B.2.4. Lipschitz estimates on $\mathcal {V}_{14}^{\nu }$

We smooth with $\mathcal {M}^{(0)}$ once more, and then we use the fundamental theorem of calculus and the smoothness of $\mathcal {R}_1^0$ to rewrite

$$\begin{aligned} \Vert \mathcal {V}_{14}^{\nu }(\varvec{\eta })-\mathcal {V}_{14}^{\nu }(\grave{\varvec{\eta }})\Vert _{H_q^1} \le C\Vert I\Vert _{L_q^2} + C\Vert I\!I\Vert _{L_q^2}, \end{aligned}$$

where

$$\begin{aligned} I := \left( \int _0^1 \big (D\mathcal {R}_1^0(\sigma +s\eta _1)-D\mathcal {R}_1^0(\sigma +s\grave{\eta }_1)\big ) \ ds\right) \eta _1^2 \end{aligned}$$

and

$$\begin{aligned} I\!I:= \left( \int _0^1 D\mathcal {R}_1^0(\sigma +s\grave{\eta }_1) \ ds\right) (\eta _1+\grave{\eta }_1)(\eta _1-\grave{\eta }_1). \end{aligned}$$

Then

$$\begin{aligned} \Vert I\Vert _{L_q^2} \le \left\| \int _0^1 \big (D\mathcal {R}_1^0(\sigma +s\eta _1)-D\mathcal {R}_1^0(\sigma +s\grave{\eta }_1)\big ) \ ds\right\| _{L^{\infty }}\Vert \eta _1\Vert _{L_q^2} \end{aligned}$$

and

$$\begin{aligned} \Vert I\!I\Vert _{L_q^2} \le \left\| \int _0^1 D\mathcal {R}_1^0(\sigma +s\grave{\eta }_1) \ ds\right\| _{L^{\infty }}\Vert \eta _1+\grave{\eta }_1\Vert _{L^{\infty }}\Vert \eta _1-\grave{\eta }_1\Vert _{L_q^2}. \end{aligned}$$

We conclude

$$\begin{aligned} \Vert I\Vert _{L_q^2} \le C\Vert \eta _1\Vert _{H_q^1}\Vert \eta _1-\grave{\eta }_1\Vert _{H_q^1} \end{aligned}$$

via a Lipschitz estimate on $D\mathcal {R}_1^0$ and

$$\begin{aligned} \Vert I\!I\Vert _{L_q^2} \le C\big (\Vert \eta _1\Vert _{H_q^1}+\Vert \grave{\eta }_1\Vert _{H_q^1}\big )\Vert \eta _1-\grave{\eta }_1\Vert _{H_q^1} \end{aligned}$$

via the boundedness of $D\mathcal {R}_1^0$.

#### B.2.5. Lipschitz estimates on $\mathcal {V}_{15}^{\nu }$

We smooth with $\mathcal {M}^{(0)}$ to estimate

$$\begin{aligned} \Vert \mathcal {V}_{15}^{\nu }(\varvec{\eta })-\mathcal {V}_{15}^{\nu }(\grave{\varvec{\eta }})\Vert _{H_q^1} \le C\nu ^{1/2}\Vert \mathcal {N}^{\nu }(\sigma +\eta _1,\zeta +\eta _2)-\mathcal {N}^{\nu }(\sigma +\grave{\eta }_1,\zeta +\grave{\eta }_2)\Vert _{L_q^2}. \end{aligned}$$

The desired estimate then follows from part (i) of Lemma B.5.

#### B.2.6. Lipschitz estimates on $\mathcal {V}_{21}^{\nu }$

This is a direct application of parts (v) and .(vi) of Lemma B.3.

#### B.2.7. Lipschitz estimates on $\mathcal {V}_{22}^{\nu }\circ \mathfrak {N}_1^{\nu }$

We have

B.5 $$\begin{aligned} \mathcal {V}_{22}^{\nu }(\mathfrak {N}_1^{\nu }(\varvec{\eta }))(X) = \mathcal {P}_1^0(\sigma +\mathfrak {N}_1^{\nu }(\varvec{\eta }))(X)-\mathcal {P}_1^0(\sigma )(X) = \frac{\alpha }{c_0}\int _X^{\infty } \mathfrak {N}_1^{\nu }(\varvec{\eta })(V) \ dV. \nonumber \\ \end{aligned}$$

The desired Lipschitz estimate on $\mathcal {V}_{22}^{\nu }$ then follows at once from the Lipschitz estimate

$$\begin{aligned} \Vert \mathfrak {N}_1^{\nu }(\varvec{\eta })-\mathfrak {N}_1^{\nu }(\grave{\varvec{\eta }})\Vert _{H_q^1} \le C\mathfrak {R}_{\star }^{\nu }(\varvec{\eta },\grave{\varvec{\eta }}), \end{aligned}$$

which we proved in Appendix B.2.1 through B.2.5. Without having substituted $\mathfrak {N}_1^{\nu }(\varvec{\eta })$ for $\eta _1$ in the process of defining $\mathfrak {N}_2^{\nu }$ in (6.6), we would have only a useless $\mathcal {O}(1)$ estimate here.

#### B.2.8. Lipschitz estimates on $\mathcal {V}_{23}^{\nu }$

This is a direct application of part (i) of Lemma B.5.

### B.3. Mapping estimates

We prove the mapping estimates that deliver part (i) of Lemma B.1 and rely mostly on the preceding Lipschitz estimates. Due to the boundedness of $\mathcal {T}^{-1}$, it suffices to show

$$\begin{aligned}&\sum _{k=1}^5 \Vert \mathcal {V}_{1k}^{\nu }(\varvec{\eta })\Vert _{H_q^1} + \Vert \mathcal {V}_{21}^{\nu }(\varvec{\eta })\Vert _{W^{1,\infty }} + \Vert \mathcal {V}_{23}^{\nu }(\varvec{\eta })\Vert _{W^{1,\infty }} \\&\qquad \le C\big (\nu ^{1/3} + \Vert \eta _1\Vert _{H_q^1}^2 + \Vert \eta _2\Vert _{W^{1,\infty }}^2\big ). \end{aligned}$$

#### B.3.1. Mapping estimates on $\mathcal {V}_{11}^{\nu }$

We estimate

$$\begin{aligned} \Vert \mathcal {V}_{11}^{\nu }(\varvec{\eta })\Vert _{H_q^1} \le \Vert \mathcal {V}_{11}^{\nu }(\varvec{\eta }) - \mathcal {V}_{11}^{\nu }(0)\Vert _{H_q^1} + \Vert \mathcal {V}_{11}^{\nu }(0)\Vert _{H_q^1}, \end{aligned}$$

where

$$\begin{aligned} \Vert \mathcal {V}_{11}^{\nu }(\varvec{\eta }) - \mathcal {V}_{11}^{\nu }(0)\Vert _{H_q^1} \le C\nu ^{1/3}\Vert \eta _1\Vert _{H_q^1}^2 \end{aligned}$$

by the Lipschitz estimates in Appendix B.2.1 and

$$\begin{aligned} \Vert \mathcal {V}_{11}^{\nu }(0)\Vert _{H_q^1} = \Vert \big (\mathcal {M}^{(\nu )}-\mathcal {M}^{(0)}\big )\big [\mathcal {R}_1^{\nu }(\sigma )\sigma \big ]\Vert _{H_q^1} \le C\nu ^{1/3} \end{aligned}$$

by Proposition 4.1.

#### B.3.2. Mapping estimates on $\mathcal {V}_{12}^{\nu }$

We estimate

$$\begin{aligned} \Vert \mathcal {V}_{12}^{\nu }(\varvec{\eta })\Vert _{L_q^2} \le \Vert \mathcal {V}_{12}^{\nu }(\varvec{\eta }) - \mathcal {V}_{12}^{\nu }(0)\Vert _{L_q^2} + \Vert \mathcal {V}_{12}^{\nu }(0)\Vert _{L_q^2}, \end{aligned}$$

where

$$\begin{aligned} \Vert \mathcal {V}_{12}^{\nu }(\varvec{\eta }) - \mathcal {V}_{12}^{\nu }(0)\Vert _{L_q^2} \le C \end{aligned}$$

by the Lipschitz estimates in Appendix B.2.1 and

$$\begin{aligned} \Vert \mathcal {V}_{12}^{\nu }(0)\Vert _{L_q^2} = \Vert \big (\mathcal {R}_1^{\nu }(\sigma )-\mathcal {R}_1^0(\sigma )\big )\sigma \Vert _{L_q^2} \le \Vert \mathcal {R}_1^{\nu }(\sigma )-\mathcal {R}_1^0(\sigma )\Vert _{L^{\infty }}\Vert \sigma \Vert _{L_q^2} \le C\nu ^{1/2} \end{aligned}$$

by part (vii) of Lemma B.4.

#### B.3.3. Mapping estimates on $\mathcal {V}_{13}^{\nu }$

Because $\mathcal {V}_{13}^{\nu }(0) = 0$, these follow from the Lipschitz estimates for $\mathcal {V}_{13}^{\nu }$ that we developed above in Appendix B.2.3.

#### B.3.4. Mapping estimates on $\mathcal {V}_{14}^{\nu }$

Because $\mathcal {V}_{14}^{\nu }(0) = 0$, these follow from the Lipschitz estimates for $\mathcal {V}_{14}^{\nu }$ that we developed above in Appendix B.2.3.

#### B.3.5. Mapping estimates on $\mathcal {V}_{15}^{\nu }$

The estimates are analogous to those in Appendix B.3.1, except now we use Lemma B.5 instead of the Lipschitz estimates in Appendix B.2.1.

#### B.3.6. Mapping estimates on $\mathcal {V}_{21}^{\nu }$

These estimates follow directly from parts (iii) and (iv) of Lemma B.3.

#### B.3.7. Mapping estimates on $\mathcal {V}_{22}^{\nu } \circ \mathfrak {N}_1^{\nu }$

We obtain these estimates by first rewriting $\mathcal {V}_{22}^{\nu } \circ \mathfrak {N}_1^{\nu }$ via the identity (B.5) and then using the mapping estimates on $\mathfrak {N}_1^{\nu }$ developed in Appendices B.3.1 through B.3.5.

#### B.3.8. Mapping estimates on $\mathcal {V}_{23}^{\nu }$

This estimate follows from part (ii) of Lemma B.5.

## Footnotes

1 For presentation purposes, the parameters *L* and *r* appearing in Merks et al. (2007) have been set to unity.

2 Here we use the abbreviation $\Vert A\Vert _{\infty } = \textstyle {\sup _{j,t}} |A_j(t)|$ and its analogues for *P* and *R*.

3 We define the width of the auxin pulse as the distance between the two points where the pulse attains 5% of its maximum value.
